# Genetic studies of plasma analytes identify novel potential biomarkers for several complex traits

**DOI:** 10.1038/srep18092

**Published:** 2016-01-04

**Authors:** Yuetiva Deming, Jian Xia, Yefei Cai, Jenny Lord, Jorge L. Del-Aguila, Maria Victoria Fernandez, David Carrell, Kathleen Black, John Budde, ShengMei Ma, Benjamin Saef, Bill Howells, Sarah Bertelsen, Matthew Bailey, Perry G. Ridge, Alzheimer’s Disease Neuroimaging Initiative (ADNI), Franz Hefti, Howard Fillit, Earl A. Zimmerman, Dzintra Celmins, Alice D. Brown, Maria Carrillo, Adam Fleisher, Stephanie Reeder, Nadira Trncic, Anna Burke, Pierre Tariot, Eric M. Reiman, Kewei Chen, Marwan N. Sabbagh, Christine M. Beiden, Sandra A. Jacobson, Sherye A. Sirrel, Rachelle S. Doody, Javier Villanueva-Meyer, Munir Chowdhury, Susan Rountree, Mimi Dang, Neil Kowall, Ronald Killiany, Andrew E. Budson, Alexander Norbash, Patricia Lynn Johnson, Robert C. Green, Gad Marshall, Keith A. Johnson, Reisa A. Sperling, Peter Snyder, Stephen Salloway, Paul Malloy, Stephen Correia, Charles Bernick, Donna Munic, Yaakov Stern, Lawrence S. Honig, Karen L. Bell, Norman Relkin, Gloria Chaing, Lisa Ravdin, Steven Paul, Laura A. Flashman, Marc Seltzer, Mary L. Hynes, Robert B. Santulli, Vernice Bates, Horacio Capote, Michelle Rainka, Karl Friedl, P. Murali Doraiswamy, Jeffrey R. Petrella, Salvador Borges-Neto, Olga James, Terence Wong, Edward Coleman, Adam Schwartz, Janet S. Cellar, Allan L. Levey, James J. Lah, Kelly Behan, Raymond Scott Turner, Kathleen Johnson, Brigid Reynolds, Godfrey D. Pearlson, Karen Blank, Karen Anderson, Thomas O. Obisesan, Saba Wolday, Joanne Allard, Alan Lerner, Paula Ogrocki, Curtis Tatsuoka, Parianne Fatica, Martin R. Farlow, Andrew J. Saykin, Tatiana M. Foroud, Li Shen, Kelly Faber, Sungeun Kim, Kwangsik Nho, Ann Marie Hake, Brandy R. Matthews, Jared R. Brosch, Scott Herring, Cynthia Hunt, Marilyn Albert, Chiadi Onyike, Daniel D’Agostino, Stephanie Kielb, Neill R Graff-Radford, Francine Parfitt, Tracy Kendall, Heather Johnson, Ronald Petersen, Clifford R. Jack, Matthew Bernstein, Bret Borowski, Jeff Gunter, Matt Senjem, Prashanthi Vemuri, David Jones, Kejal Kantarci, Chad Ward, Sara S. Mason, Colleen S. Albers, David Knopman, Kris Johnson, Howard Chertkow, Chris Hosein, Jacob Mintzer, Kenneth Spicer, David Bachman, Hillel Grossman, Effie Mitsis, Nunzio Pomara, Raymundo Hernando, Antero Sarrael, William Potter, Neil Buckholtz, John Hsiao, Smita Kittur, James E. Galvin, Brittany Cerbone, Christina A. Michel, Dana M. Pogorelec, Henry Rusinek, Mony J de Leon, Lidia Glodzik, Susan De Santi, Nancy Johnson, Chuang-Kuo, Diana Kerwin, Borna Bonakdarpour, Sandra Weintraub, Jordan Grafman, Kristine Lipowski, Marek-Marsel Mesulam, Douglas W. Scharre, Maria Kataki, Anahita Adeli, Jeffrey Kaye, Joseph Quinn, Lisa Silbert, Betty Lind, Raina Carter, Sara Dolen, Michael Borrie, T-Y Lee, Rob Bartha, Walter Martinez, Teresa Villena, Carl Sadowsky, Zaven Khachaturian, Brian R. Ott, Henry Querfurth, Geoffrey Tremont, Richard Frank, Debra Fleischman, Konstantinos Arfanakis, Raj C. Shah, Leyla deToledo-Morrell, Greg Sorensen, Elizabeth Finger, Stephen Pasternack, Irina Rachinsky, Dick Drost, John Rogers, Andrew Kertesz, Ansgar J. Furst, Stevan Chad, Jerome Yesavage, Joy L. Taylor, Barton Lane, Allyson Rosen, Jared Tinklenberg, Sandra Black, Bojana Stefanovic, Curtis Caldwell, Ging-Yuek Robin Hsiung, Benita Mudge, Michele Assaly, Nick Fox, Susan K. Schultz, Laura L. Boles Ponto, Hyungsub Shim, Karen Ekstam Smith, Jeffrey M. Burns, Russell H. Swerdlow, William M. Brooks, Daniel Marson, Randall Griffith, David Clark, David Geldmacher, John Brockington, Erik Roberson, Marissa Natelson Love, Charles DeCarli, Owen Carmichael, John Olichney, Pauline Maillard, Evan Fletcher, Dana Nguyen, Andrian Preda, Steven Potkin, Ruth A. Mulnard, Gaby Thai, Catherine McAdams-Ortiz, Susan Landau, William Jagust, Liana Apostolova, Kathleen Tingus, Ellen Woo, Daniel H.S. Silverman, Po H. Lu, George Bartzokis, Paul Thompson, Michael Donohue, Ronald G. Thomas, Sarah Walter, Devon Gessert, James Brewer, Helen Vanderswag, Tamie Sather, Gus Jiminez, Archana B. Balasubramanian, Jennifer Mason, Iris Sim, Paul Aisen, Melissa Davis, Rosemary Morrison, Danielle Harvey, Lean Thal, Laurel Beckett, Thomas Neylan, Shannon Finley, Michael W. Weiner, Jacqueline Hayes, Howard J. Rosen, Bruce L. Miller, David Perry, Dino Massoglia, Olga Brawman-Mentzer, Norbert Schuff, Charles D. Smith, Peter Hardy, Partha Sinha, Elizabeth Oates, Gary Conrad, Robert A. Koeppe, Joanne L. Lord, Judith L. Heidebrink, Steven E. Arnold, Jason H. Karlawish, David Wolk, Christopher M. Clark, John Q. Trojanowki, Leslie M. Shaw, Virginia Lee, Magdalena Korecka, Michal Figurski, Arthur W. Toga, Karen Crawford, Scott Neu, Lon S. Schneider, Sonia Pawluczyk, Mauricio Beccera, Liberty Teodoro, Bryan M. Spann, Kyle Womack, Dana Mathews, Mary Quiceno, Norm Foster, Tom Montine, J. Jay Fruehling, Sandra Harding, Sterling Johnson, Sanjay Asthana, Cynthia M. Carlsson, Eric C. Petrie, Elaine Peskind, Gail Li, Anton P. Porsteinsson, Bonnie S. Goldstein, Kim Martin, Kelly M. Makino, M. Saleem Ismail, Connie Brand, Amanda Smith, Balebail Ashok Raj, Kristin Fargher, Lew Kuller, Chet Mathis, Mary Ann Oakley, Oscar L. Lopez, Donna M. Simpson, Kaycee M. Sink, Leslie Gordineer, Jeff D. Williamson, Pradeep Garg, Franklin Watkins, Nigel J. Cairns, Marc Raichle, John C. Morris, Erin Householder, Lisa Taylor-Reinwald, David Holtzman, Beau Ances, Maria Carroll, Mary L. Creech, Erin Franklin, Mark A. Mintun, Stacy Schneider, Angela Oliver, Ranjan Duara, Daniel Varon, Maria T. Greig, Peggy Roberts, Pradeep Varma, Martha G. MacAvoy, Richard E. Carson, Christopher H. van Dyck, Peter Davies, David Holtzman, John C. Morris, Kelly Bales, Eve H. Pickering, Jin-Moo Lee, Laura Heitsch, John Kauwe, Alison Goate, Laura Piccio, Carlos Cruchaga

**Affiliations:** 1 Department of Psychiatry, Washington University School of Medicine, 660 S. Euclid Ave. B8134, St. Louis, MO 63110, USA; 2 Department of Neurology, Xiangya Hospital, Central South University, Changsha, Hunan 410008, P.R. China; 3 Human Genetics Programme, Wellcome Trust Sanger Institute, Cambridge, CB10 1SA, UK; 4 Department of Biology, Brigham Young University, Provo, UT, USA; 5 Department of Neurology, Washington University School of Medicine, 660 S. Euclid Ave., St. Louis, MO 63110, USA; 6 Department of Developmental Biology, Washington University School of Medicine, 660 S. Euclid Ave., St. Louis, MO 63110, USA; 7 Knight Alzheimer’s Disease Research Center, Washington University School of Medicine, 4488 Forest Park Ave., St Louis, MO 63108, USA; 8 Hope Center for Neurological Disorders. Washington University School of Medicine, 660 S. Euclid Ave. B8111, St. Louis, MO 63110, USA; 9 Neuroscience Research Unit, Worldwide Research and Development, Pfizer, Inc., Groton, CT, USA; 10 Acumen Pharmaceuticals; 11 AD Drug Discovery Foundation; 12 Albany Medical College; 13 Alzheimer's Association; 14 Banner Alzheimer's Institute; 15 Banner Sun Health Research Institute; 16 Baylor College of Medicine; 17 Boston University; 18 Brigham and Women's Hospital; 19 Brown University; 20 Butler Hospital; 21 Cleveland Clinic; 22 Columbia University Medical Center; 23 Cornell University; 24 Dartmouth Hitchcock Medical Center; 25 Dent Neurologic Institute; 26 Department Of Defense; 27 Duke University Medical Center; 28 Eli Lilly; 29 Emory University; 30 Georgetown University Medical Center; 31 Hartford Hospital; 32 Harvard Medical School; 33 Howard University; 34 Indiana University; 35 Johns Hopkins University; 36 Mayo Clinic, Jacksonville; 37 Mayo Clinic, Rochester; 38 McGill University; 39 Medical University South Carolina; 40 Mount Sinai School of Medicine; 41 Nathan Kline Institute; 42 National Institute of Mental Health; 43 National Institute on Aging; 44 Neurological Care of CNY; 45 New York University; 46 Northwestern University; 47 Ohio State University; 48 Oregon Health and Science University; 49 Parkwood Hospital; 50 Premiere Research Institute; 51 Prevent Alzheimer's Disease 2020 Inc.; 52 Rhode Island Hospital; 53 Richard Frank Consulting; 54 Rush University Medical Center; 55 Siemens; 56 St Joseph's Health Care Ontario; 57 Stanford University; 58 Sunnybrook Health Sciences Ontario; 59 U.B.C. Clinic for AD & Related Disorders; 60 University College London; 61 University Iowa College Medicine; 62 University Kansas Medical Center; 63 University of Alabama at Birmingham; 64 University of California, Davis - Sacramento; 65 University of California, Irvine; 66 University of California, Berkeley; 67 University of California, Los Angeles; 68 University of California, San Diego; 69 University of California, San Francisco; 70 University of Kentucky; 71 University of Michigan; 72 University of Pennsylvania; 73 University of Pennsylvania School of Medicine; 74 University of Southern California; 75 University of Texas Southwestern Medical School; 76 University of Utah; 77 University of Washington; 78 University of Wisconsin; 79 University Rochester Medical Center; 80 University South Florida; 81 University of Pittsburgh; 82 Wake Forest University; 83 Washington University, St. Louis; 84 Wien Center; 85 Yale School of Medicine; 86 Yeshiva University

## Abstract

Genome-wide association studies of 146 plasma protein levels in 818 individuals revealed 56 genome-wide significant associations (28 novel) with 47 analytes. Loci associated with plasma levels of 39 proteins tested have been previously associated with various complex traits such as heart disease, inflammatory bowel disease, Type 2 diabetes, and multiple sclerosis. These data suggest that these plasma protein levels may constitute informative endophenotypes for these complex traits. We found three potential pleiotropic genes: *ABO* for plasma SELE and ACE levels, *FUT2* for CA19-9 and CEA plasma levels, and *APOE* for ApoE and CRP levels. We also found multiple independent signals in loci associated with plasma levels of ApoH, CA19-9, FetuinA, IL6r, and LPa. Our study highlights the power of biological traits for genetic studies to identify genetic variants influencing clinically relevant traits, potential pleiotropic effects, and complex disease associations in the same locus.

Plasma proteins play important roles in numerous biological pathways, contribute to risk for many diseases, and have long been used for clinical risk assessment, diagnosis, prognosis and evaluation of treatment efficacy. Protein levels used as a quantitative trait in genome-wide association studies (GWAS) can act as an intermediate phenotype that functionally links genetic variation to disease-predisposing factors and then to complex disease end points^1^,^2^. Therefore, studies that link genetic variants with protein traits may provide a means to reveal the underlying mechanisms of the GWAS findings.

Previous case-control studies have associated many loci with various complex diseases. Unfortunately the effect sizes of genetic associations with complex disorders are generally small and the functional information on the underlying biological processes is often unclear or absent, which complicates the interpretation of the results. As a result, the focus of GWAS is now shifting increasingly away from studying associations with disease end points and toward associations with intermediate traits that are known risk factors for disease^3^,^4^,^5^.

A previous study used GWAS data and various commercially available enzyme-linked immunosorbent assay (ELISA) kits to find genetic variants associated with plasma or serum levels of 42 different proteins (such as interleukin 18, insulin, and leptin) implicated in various complex diseases (such as lupus, diabetes, and obesity)^6^. They identified several GWAS hits that could help in understanding the biology of those complex traits^6^. Recent technological developments have made possible the quantification of multiple proteins in a single analytical procedure, allowing both broader and deeper molecular profiling of large cohorts^2^,^7^,^8^,^9^,^10^. Genetic analyses of these data have discovered numerous genomic regions associated with clinically relevant proteins, with recent large-scale proteome analyses having identified many loci associated with serum and plasma concentrations of individual proteins^2^,^7^,^8^,^9^,^10^. Nevertheless, our understanding of the genetic basis and pathophysiological impact of variations in protein levels remains far from complete. Most of these studies limited analyses to *cis* variants or focused on candidate regions rather than genome-wide scans^2^,^7^,^8^,^9^. Recent research suggests the importance of investigating protein phenotypes beyond those used in traditional genetic studies^10^.

Here we present the results of an unbiased large genetic investigation of protein phenotypes in 818 unrelated individuals from the Washington University Knight Alzheimer’s Disease Research Center (KADRC) and Alzheimer’s Disease Neuroimaging Initiative (ADNI) who were analyzed for both genome-wide SNP genotypes and for 146 phenotypic measures obtained from multi-analyte panels (Human DiscoveryMAP) of human plasma samples.

## Results

Before any genetic analyses we performed extensive quality control (QC) in the genotype and phenotype data. After log transformation and standardization (see materials and methods) we confirmed that the protein levels followed a normal distribution. We also tested the correlation between the analyte values and covariates such as age, gender, and Alzheimer’s disease (AD) status (Supplementary Tables S1 and S2). Age, gender, disease status, study, and principal components factors (PCs) from population stratification were included as covariates.

We decided to perform a one-stage GWAS rather than a two-stage GWAS because 1) we have GWAS for all the samples, and 2) it has been shown that combining data from both stages of a two-stage GWAS to perform a single analysis almost always has increased power to identify genetic association than analyzing the groups separately even though a lower statistical threshold is required to determine significance^11^. So to maximize our statistical power, we combined the two datasets to perform a joint one-stage GWAS with all 818 individuals from ADNI and KADRC (characteristics shown in Table 1). To verify our results, we followed up with additional analyses stratified by study and performed meta-analyses of the results from each dataset for each analyte, and we found that the p-values from the meta-analyses were similar to the joint GWAS p-values (Supplementary Table S3). In order to avoid spurious association and consider a single nucleotide polymorphism (SNP) as a real signal, we required each genome-wide significant association from the joint analysis to meet additional criteria: 1) the SNP association had to be consistent between the two series, in the same direction and with similar effect size, which represents an internal replication (Supplementary Table S3) and 2) since we were using cohorts from AD studies, we wanted to be sure our results were not confounded by AD status. In addition to using AD status as a covariate in our initial analyses, we performed separate GWAS on cases and controls and found no difference in effect size or direction indicating the associations found in the combined GWAS were not confounded by AD status (Supplementary Table S4).

We decided to use the common threshold for genome-wide significance (p < 5.0 × 10^−8^) instead of p < 3.42 × 10^−10^ (Bonferroni multiple test correction taking into account SNPs and phenotypes) because the latter would consider that all the analytes are independent and not correlated. However there is extensive evidence that this is not the case and in a recent study we demonstrated that some analytes are highly correlated^12^. Additionally five of the associations in this study in the p = 5 × 10^−8^–3.42 × 10^−10^ range have been previously reported and others are located in receptors and genes known to regulate levels of the analyte (Table 2 and Supplementary Table S5) which indicate that these are real signals. We also found complex loci and potential pleiotropic effects that support the evidence that not all of the SNPs and analytes act independently of others. These findings suggest that a multiple test correction threshold of p < 3.42 × 10^−10^ would be too stringent. For this reason we decided to report all the loci with a p < 5.0 × 10^−8^, but we also highlight on Table 2 those that pass the p < 3.42 × 10^−10^ threshold.

### Genome-wide association study results

After performing the linear regression with each analyte as a phenotype, there were 56 genome-wide significant loci for 47 analytes (Table 2). Twenty-eight of these associations have been reported in the literature previously and 28 (50%) were novel. Thirty-two of the 56 associations (9 novel) pass the p < 3.42 × 10^−10^ threshold.

### Previously reported findings

**Figure 1:**
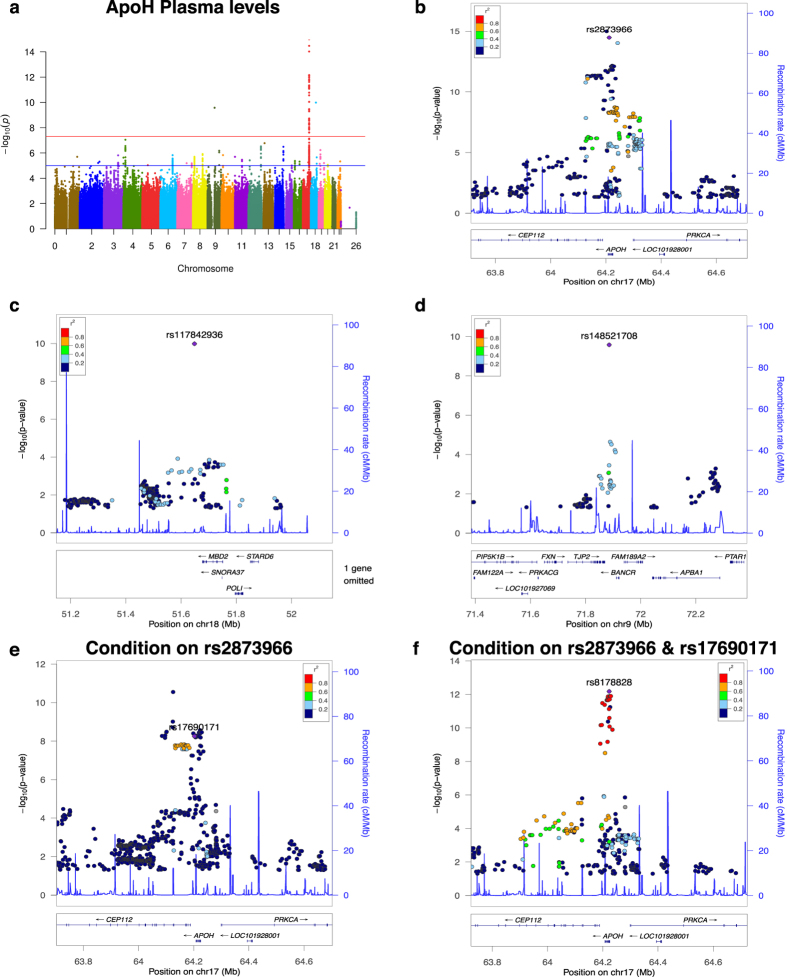
Manhattan and regional plots for associations with plasma levels of ApoH. (**a**) Manhattan plot of −log_10_ p-values for association with plasma levels of ApoH levels; (**b**) Regional plot for genome-wide significant association on chromosome 17 with ApoH plasma levels; (**c**) Regional plot for genome-wide significant association on chromosome 18 with ApoH plasma levels; (**d**) Regional plot for genome-wide significant association on chromosome 9 with ApoH plasma levels; (**e**) Regional plot for genome-wide significant association on chromosome 17 after conditioning for rs2873966; (**f**) Regional plot for genome-wide significant association on chromosome 17 after conditioning for rs2873966 and rs17690171.

Twenty-eight of our genome-wide signals replicated associations reported by 14 different genetic studies of plasma or serum protein levels in humans (Table 2 and Supplementary Table S5)^6^,^9^,^13^,^14^,^15^,^16^,^17^,^18^,^19^,^20^,^21^,^22^,^23^,^24^. Six of our most significant SNPs were the same SNP reported previously and the remaining SNPs were in linkage disequilibrium (LD) with reported SNPs (Supplementary Table S5). Fifteen of these 28 genome-wide loci had p < 3.42 × 10^−10^ in our study and five others were in the p = 5 × 10^−8^ to 3.42 × 10^−10^ range, indicating that signals in this range in our study constitute strong associations. Twenty-three of these previously reported loci are in *cis* (within 1MB of the gene that encodes the protein) and five are in *trans* (Table 2, Fig. 1, and Supplementary Figs S1–26). Twelve (52%) of the *cis* effects are coding variants (nine missense) and four of the *trans* effects are coding variants (three missense; Table 2). None of the *trans* effects are located in untranslated regions (UTR) but two of the analytes had *cis* effects that are in the UTR (CD40: 5′ UTR of *CD40* and HCC4: 3′ UTR of *CCL16*). All of the *trans* effects are located within genes (four coding, one intronic) that have interactions with the analyte that are not known or well understood (Table 2). However our results and the previous published studies suggest that these loci in *trans* proteins play an important role in regulating the levels of CA19-9, CEA, CRP, SELE, and ACE in plasma^15^,^17^,^21^,^24^. More interestingly, some of these loci, like *ABO* or *FUT2*, are genome-wide for more than one analyte, which also indicates that these may constitute master regulatory signals (see pleiotropic section).

### Novel findings

We found 28 loci associated with 25 analytes that have not been reported previously (Table 2, Fig. 1, and Supplementary Figs S8, S12, S21, S22, S27–36). Of these novel associations nine pass the p < 3.42 × 10^−10^ threshold (Table 2). All of the associations were highly consistent (same effect size or beta) between the two datasets which represents an internal replication (Supplementary Table S3) and were not confounded by AD status (Supplementary Table S4).

Five of our 28 novel findings were *cis* effects (one coding variant and four intergenic; Table 2): 1) rs926144 which is 29.7 KB from *SERPINA1* was significantly associated with plasma levels of AAT (p = 4.71 × 10^−12^; Supplementary Fig. S27), 2) rs2015086 within 2 KB upstream of *CCL18* was significantly associated with MIP1a levels in plasma (p = 2.56 × 10^−15^; Supplementary Fig. S38), 3) a missense variant in *AGER* (rs2070600) was associated with plasma RAGE levels (p = 1.86 × 10^−11^; Supplementary S40), 4) rs646776 which is located 33.7 KB from *SORT1* was significantly associated with Sortilin plasma levels (p = 2.20 × 10^−9^; Supplementary Fig. S41), and 5) rs409336 located 3.7 KB from *CXCL5* was significantly associated with ENA78 plasma levels (p = 1.11 × 10^−8^; Supplementary Fig. S30).

Twenty-three of our 28 novel findings were *trans* effects. Twelve analytes were associated with loci that contained only intergenic SNPs and eleven analytes (ANG2, BLC, CEA, F7, FGF4, GROa, MIP1b, MMP7, RAGE, THPO, TNC) were associated with SNPs on intronic regions in gene-rich areas. Interestingly some of these loci contain intronic SNPs that are likely to be regulatory based on RegulomeDB^25^: *SCARA5* (associated with TNC levels) and *PARVG* (associated with ANG2 levels) contain SNPs with RegulomeDB^25^ scores lower than 3 (Supplementary Table S6). Plasma MIP1b levels were also associated with a locus that contains SNPs that are likely to be regulatory. We found that rs145617407, located in the intron of *CCR3*, was significantly associated with MIP1b levels in plasma (p = 2.58 × 10^−10^) and this SNP is located less than 119 KB from *CCR5* which is the receptor for CCL4/MIP1b (Supplementary Fig. S21).

### GWAS Conditional on top hits revealed additional signals within same loci

We then performed conditional analyses to determine whether more than one signal in the same loci exists. When we added the most significant SNP to the linear regression model, five analytes (ApoH, CA19-9, FetuinA, IL6r, and LPa) still showed independent and genome-wide significant SNPs at the same locus (Fig. 1, Table 3 and Supplementary Fig. S5, S13, S17 and S19). It is interesting to note that three of four of the complex loci we found were in *cis* with the respective protein whereas the *FUT2/FUT6/FUT3* locus was associated with CA19-9 plasma levels. Since we decided to use the traditional genome-wide p-value threshold (p < 5 × 10^−8^) for the conditional analyses, we may be missing some additional independent signals.

After conditioning on rs2070633, located in an *AHSG* intron, we found that rs4917, a missense variant also located in *AHSG*, was still significantly associated with plasma levels of FetuinA (p = 7.27 × 10^−9^, original p = 2.61 × 10^−42^; Table 3 and Supplementary Fig. S13). After conditioning on both SNPs no additional signals were found. An intronic variant in *IL6R*, rs7526131, was still significantly associated with IL6r plasma levels after conditioning on rs12126142, also located in an intron of *IL6R* (p = 1.43 × 10^−10^, original p = 4.47 × 10^−72^; Table 3 and Supplementary Fig. S17). Plasma levels of LPa were significantly associated with rs783147, located in an intron of *PLG* 506 KB from *LPA*, and after conditioning on this SNP an intronic variant of *SLC22A1* approximately 0.4 MB from *LPA* (rs783147), was still significantly associated with LPa levels (p = 1.64 × 10^−9^, original p = 9.86 × 10^−9^; Table 3 and Supplementary Fig. S19).

We found two analytes (ApoH and CA19-9) that the genome-wide locus contained up to three independent signals (Fig. 1, Table 3 and Supplementary Fig. S5). All three signals in the ApoH analyses contained missense variants located in *APOH* (rs52797880: I141T, p = 1.57 × 10^−12^; rs1801690: W335S, p = 5.15 × 10^−9^, original p = 2.77 × 10^−11^; rs8178847: R154H, p = 2.20 × 10^−12^, original p = 1.57 × 10^−12^; Fig. 1). As reported above, the initial signal in the CA19-9 GWAS contained a missense variant in *FUT2*. After conditioning on the most significant SNP (rs485073, p = 2.12 × 10^−23^) from the CA19-9 GWAS, the new signal contained a synonymous variant located in *FUT6* (rs112313064, p = 3.79 × 10^−26^, original p = 7.46 × 10^−23^), and conditioning on the two SNPs resulted in a separate signal upstream of *FUT3* (rs2306969, p = 2.78 × 10^−9^, original p = 6.11 × 10^−23^; Supplementary Fig. S5). All of these results indicate these protein levels are highly regulated and that different and independent regulation mechanisms, even at the same locus, are in place: some mechanisms may act by affecting cleavage or receptor binding (non-synonymous variants) and others by regulating gene expression (non-coding variants).

### Potential pleiotropy

**Figure 2:**
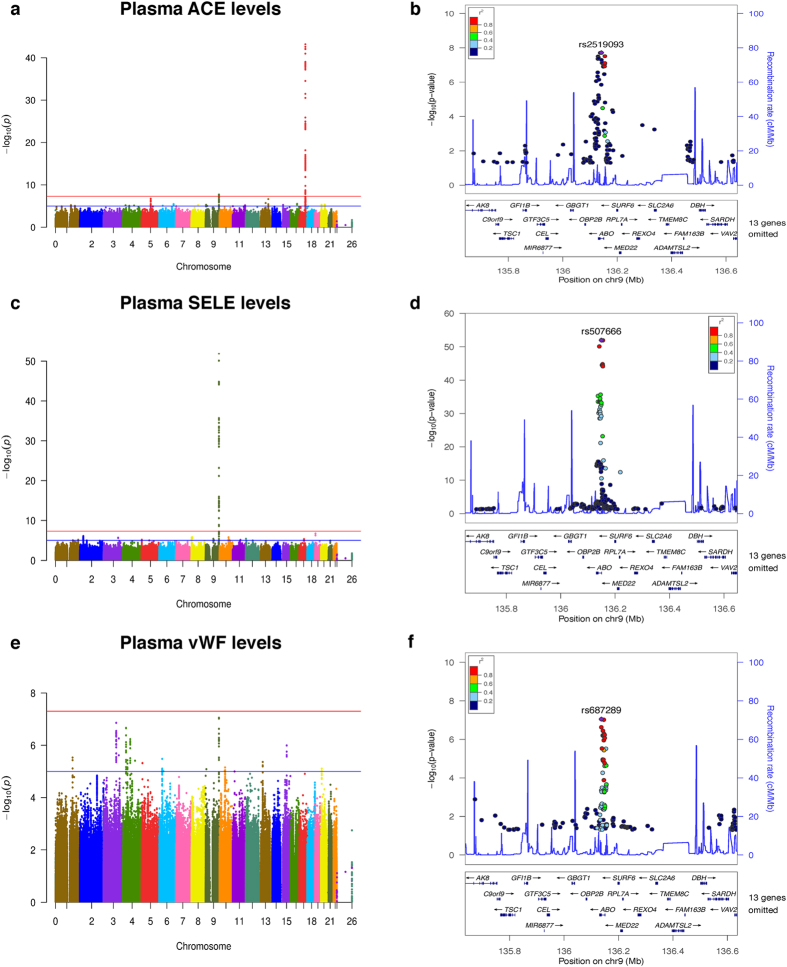
Manhattan and regional plots for pleiotropic *ABO* variant associations with plasma levels of ACE, SELE, and vWF. (**a**) Manhattan plot of −log_10_ p-values for association with plasma levels of ACE; (**b**) Regional plot for genome-wide significant associations in *ABO* locus with ACE plasma levels; (**c**) Manhattan plot of −log_10_ p-values for association with plasma levels of SELE; (**d**) Regional plot for genome-wide significant associations in *ABO* locus with SELE plasma levels; (**e**) Manhattan plot of -log_10_ p-values for association with plasma levels of vWF; (**f**) Regional plot for associations in *ABO* locus with vWF plasma levels, rs687289 was close to genome-wide significance (p = 8.87 × 10^−8^).

In addition to finding that some proteins have complex regulation within the structural gene (or a different gene in the case of CA19-9), we also found potentially pleiotropic effects with one gene affecting more than one protein. Potential pleiotropic effects were found for three groups of analyte/associations even though the analyte levels were not correlated: *ABO* associated with plasma levels of SELE, ACE, and vWF (p = 1.01 × 10^−52^, beta = –0.882; p = 1.90 × 10^−8^, beta = –0.352; p = 8.87 × 10^−8^, beta = 0.253 respectively; Table 4 and Fig. 2). *ABO* has been previously reported to be associated with ACE activity^26^ and SELE plasma and serum levels^15^,^17^. *ABO* has also been associated with vWF plasma levels, and although the locus did not reach genome-wide significance in our analysis it was very close^27^.

*FUT2* was associated with plasma levels of CA19-9 and CEA (p = 2.12 × 10^−23^, beta = −0.509; p = 4.07 × 10^−16^, beta = −0.406 respectively; Table 4 and Supplementary Fig. S5, S8); and the *APOE* region was associated not only with plasma levels of ApoE but also CRP (p = 2.76 × 10^−26^, beta = −0.594; p = 6.69 × 10^−9^, beta = −0.354 respectively; Table 4 and Supplementary Fig. S4, S10).

Interestingly none of these analyte pairs or trios are highly correlated (r < 0.25; Table 4), which again supports the idea that these loci (*ABO*, *FUT2*, and *APOE-TOMM40* region) are truly master-regulatory regions, that protein levels are highly and complexly regulated, and that studying the genetic architecture of biological traits can lead to a deeper knowledge of the biological processes.

### Impact of these findings with complex diseases

Of the 56 loci that we found associated with plasma protein levels, 46 loci have also been reported to be associated with complex traits and diseases including coronary artery disease (ACE and SELE), stroke (ACE and SELE), various cancers (ACE, CA19-9, CEA, RAGE, and SELE), age-related macular degeneration (ApoE, CFHR1, and CRP), periodontitis (ApoH), multiple sclerosis (BLC and CD40), inflammatory bowel disease (CD40 and ENA78), and Type 2 diabetes (IL13, MCSF, and RAGE) (Table 5; see supplementary results for a complete description). As an example, the *AGER* variant rs2070600, which in our study was associated with plasma RAGE levels (p = 1.86 × 10^−11^) has been reported to be associated with pulmonary function^28^. A recent study of RAGE plasma levels suggests they are a promising biomarker for acute respiratory distress syndrome, supporting our hypothesis^29^.

Similarly our genetic analysis for BLC revealed a significant association with SNPs located in *DDAH1* (rs7541151, p = 6.44 × 10^−9^; Table 2), a gene that has been associated with multiple sclerosis (MS). Interestingly BLC levels have recently been reported to be different between patients with MS and controls^30^, which further supports BLC as a potential biomarker.

**Figure 3:**
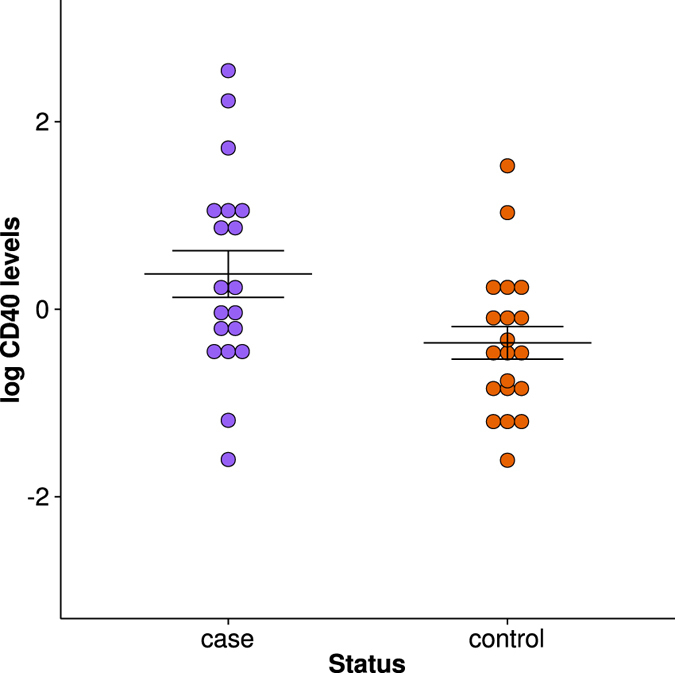
Plasma levels of CD40 in MS cases versus controls.

Since levels of CD40 in plasma were associated with the *CD40* locus and *CD40* variants have been associated with MS in three independent GWAS^30^,^31^,^32^, we hypothesized that plasma levels of CD40 may also be associated with MS status. As a proof of concept, we used a Quantikine sandwich ELISA kit (R&D Systems cat #DCCD40) to measure plasma levels of CD40 in 20 individuals with relapsing remitting MS in remission at time of plasma collection (8 male, 12 female; mean age = 44.45 ± 15.51 years) and 20 healthy controls (8 male, 12 female; mean age = 41.84 ± 11.52 years; Supplementary Table S7). We used linear regression to determine if log values of plasma CD40 levels were significantly different between MS cases and controls, with age and gender as covariates. We found plasma levels of CD40 were significantly higher in MS cases (753.26 ± 235.71 pg/mL) than controls (603.02 ± 139.01 pg/mL; p = 0.041, beta = −1.837; Fig. 3), supporting our hypothesis.

More than half of the loci associated with plasma protein levels in our study have been previously reported to be associated with various complex diseases. Based on the current knowledge for RAGE and BLC, and in the concept of Mendelian randomization, we hypothesize that these protein levels constitute informative biomarkers for these complex traits although additional studies would be necessary to validate this hypothesis. More detailed information about potential novel biomarkers for complex traits is included in Supplementary Results and analyte abbreviations with full names are in Supplementary Table S8.

## Discussion

GWAS of complex traits have been very successful in identifying novel loci associated with those traits, but these studies require extremely large sample sizes, and in some cases it is difficult to interpret the results because the associations are with surrogate tag SNPs which may not be the causal SNPs. Many loci contain multiple genes which also makes it difficult to determine the causal gene or variant. Additionally some loci are located in non-protein coding regions where functional effects are poorly understood. Genetic analyses of biological traits may provide more power than traditional GWAS and may be more informative about the biological effects for specific loci. Using a more unbiased approach than previous genetic studies, we were able to replicate many previously reported associations with various plasma protein levels and uncover several novel associations that could warrant further research. The results from our careful analyses suggest that even though we utilized two datasets from Alzheimer’s disease studies there was no confounding effect due to disease status or dataset. Combining datasets from high-throughput technologies that deliver genome-wide genetic data and quantification of protein levels in a single procedure provides a great deal of power to analyses that may help researchers understand the biology of complex traits including the complex loci involved and pleiotropic effects.

Our results clearly indicate that the protein levels are highly and complexly regulated. We found master regulatory regions (pleiotropic; Table 4, Fig. 2, and Supplementary Fig. S4, S10) as well as several independent regulatory elements in the same locus for the same proteins (Table 3, Fig. 1, and Supplementary Fig. S5, S13, S17 and S19). We found protein levels associated with variants in or near the gene coding that protein (*cis* effects) as well as variants located elsewhere in the genome (*trans* effects) demonstrating that protein levels are not only affected by the genes that encode the protein but also by interaction with other proteins as in the case of *ABO* or *FUT2* (Table 4).

Interestingly, we found that for almost half of the *cis* effects (13 out of 28), the association could be explained by a coding variant but for the *trans* effects most of the loci (24 out of 28) only contain regulatory variants (Table 2). Although these non-coding signals could be synthetic association and are being driven by low frequency variants, our results and those recently published by ENCODE and the GTEx consortium would suggest that those associations are likely to affect gene expression^33^,^34^. For this same reason, it is more likely that the association in *cis* (more frequently due to a non-synonymous variant) will present a higher effect size and are easier to identify in a genetic study than a *trans* signal, which is more likely to affect gene expression through regulation.

Table 2 shows that most of the *trans* effects associated with plasma protein levels had less significant p-values and lower betas than most of the *cis* effects. This could explain why only three of the *trans* effects we found were previously reported while the other 24 were novel. It is of vital importance to identify *trans* effects because that will help us to identify novel biological interactions and pathways. Of the 28 *trans* effects we found in our study, only one corresponded to a protein that constituted the receptor of the studied analyte or a gene known to interact directly with the analyte (rs145617407 located less than 119 KB from *CCR5* which is the receptor for CCL4/MIP1b)^35^. However, the fact that the associations of SELE, ACE, and vWF with the *ABO* locus or CA19-9 and CEA with *FUT2* have been identified in other studies, indicates that these signals are real and some of these novel loci may be implicated in regulating the levels of one or more proteins. Additional work is needed because currently it is not clear how *ABO* regulates plasma levels of SELE, ACE, and vWF or how *FUT2* regulates CEA and CA19-9 levels. For the novel loci this can be more complicated because several signals are located in very gene-rich regions and several genes could drive the association (Fig. 1 and Supplementary Fig. S1, S6, S8, S10, S21, S24, S28, 29, S33, S36-S37, S42, S44, 46).

Another important finding related to this study is its implication on complex traits. Proteins play a key role in many complex traits, so understanding the genetic variations associated with protein levels is important in understanding the biological basis of these traits. We used the concepts of Mendelian randomization, our data, and the data from the NHGRI GWAS catalog to identify genetic regions that are genome-wide significant for various analyte levels as well as previously associated with complex traits. While most of these loci have been associated with complex traits, the associations of most of the plasma analytes with the complex traits have not been previously reported. Our results suggest that some of these plasma protein levels could be novel biomarkers or even endophenotypes for these complex traits.

As an example of our approach providing information useful for understanding potential pleiotropic effects in promising biomarkers for complex diseases that has been supported by previous research: rs485073 in *FUT2* was associated in our study with plasma levels of both CEA and CA19-9, which are only weakly correlated in plasma (r = 0.166, p = 2.98 × 10^−6^). This potential pleiotropy strongly suggests that rs485073 is part of a master regulatory region. In this case this means that plasma levels of CEA and CA19-9 could be important for understanding gastric cancer because *FUT2* variants have also been associated with gastric cancer risk^36^. This is further supported by the fact that both CEA and CA19-9 have been reported as FDA approved biomarkers for other types of cancer^37^.

We found several promising plasma biomarkers for complex traits including IL13, ENA78, BLC, and CD40. Based on our results, plasma levels of IL13 may be informative in Type 2 diabetes research. We found rs7433647, located near *UBE2E2*, was associated with IL13 plasma levels (p = 1.21 × 10^−8^). *UBE2E2* has previously been associated with Type 2 diabetes in a large GWAS meta-analysis of more than 26,000 cases and 83,000 controls with varied ancestry^38^. A recent study using a mouse model for Type 2 diabetes suggests that expression of IL13 plays a key role in adipose tissue inflammation and insulin resistance, further supporting the idea that IL13 levels may be important in studying Type 2 diabetes^39^. ENA78/CXCL5 expression is elevated in the inflamed tissues of patients with rheumatoid arthritis, ulcerative colitis and Crohn’s disease^40^,^41^. Several studies have reported association of *CXCL5* variants with inflammatory bowel disease and metabolite levels^42^,^43^. In our study rs409336, near the *CXCL5* gene, showed the strongest effect on plasma ENA78/CXCL5 levels. Because of the similarity in genetic influences on ENA78/CXCL5 levels and inflammatory bowel disease, it is possible that these traits share a common pathophysiological pathway and our findings support further investigation of the involvement of ENA78/CXCL5 in the etiology of inflammatory bowel disease.

We found two promising plasma protein biomarkers for MS: BLC and CD40. In our study rs7541151 in *DDAH1* was associated with plasma BLC levels. *DDAH1* is responsible for the degradation of ADMA into citrulline and dimethylamine, and previous studies showed an association of *DDAH1* variants with MS and ADMA levels^30^,^44^. Previous studies indicate that CSF levels of BLC/CXCL13 may be an informative biomarker for studying treatment effects in MS^45^,^46^,^47^. Our results indicate plasma BLC/CXCL13 levels may be informative as well. The *CD40* locus has been associated with MS^30^,^31^,^32^ but our study appears to be the first to associate CD40 plasma levels with *CD40* variants. Plasma levels of CD40 have not been reported as a potential biomarker for MS, but our preliminary data suggests they may be a biomarker for MS. Although we did find a significant difference in CD40 levels in plasma between MS cases and controls, our sample size was small and only contained patients in remission so it would be prudent to evaluate a larger, more varied cohort to determine the possible utility of plasma levels of CD40 as an MS biomarker.

## Methods

### Ethics Statement

The Institutional Review Board (IRB) at the Washington University School of Medicine in Saint Louis approved the study. Research was carried out in accordance with the approved protocol. A written informed consent was obtained from participants and their family members by the Clinical Core of the Charles F. and Joanne Knight Alzheimer’s Disease Research Center (Knight-ADRC). The approval number for the Knight-ADRC Genetics Core family studies is 93-0006. The MS and control patients have signed the consent for the MS repository, approval number 201104379.

### Cohort descriptions

Demographics of the samples included in this manuscript are reported in Table 1.

### Washington University Knight Alzheimer’s Disease Research Center (KADRC) cohort

The KADRC sample included 124 AD cases and 188 cognitively normal controls. These individuals were evaluated by Clinical Core personnel of Washington University. Cases received a clinical diagnosis of Alzheimer’s disease in accordance with standard criteria and dementia severity was determined using the Clinical Dementia Rating (CDR)^48^. Plasma from all KADRC samples was collected in the morning after an overnight fast, immediately centrifuged, and stored at −80°C until assayed according to standard procedures^49^.

### Alzheimer’s Disease Neuroimaging Initiative (ADNI) cohort

The ADNI sample included 434 AD cases and 72 cognitively normal controls. Data used in the preparation of this article were obtained from the ADNI database (http://adni.loni.usc.edu/). See Supplementary Methods for further information about ADNI’s methods and for up-to-date information see http://www.adni-info.org/. Plasma was collected in the morning after an overnight fast, immediately centrifuged, and stored at −80°C until assayed as described previously^9^. Genetic and phenotypic data for 506 samples was available for this study.

### Genotyping and Quality Control

The ADNI protocol for collecting genomic DNA samples has been previously described^50^. All ADNI samples were genotyped using the Illumina Human610-Quad BeadChip, which contains over 600,000 SNP markers. KADRC samples were genotyped with the Human610-Quad BeadChip or the Omniexpress chip^51^. Prior to association analysis, all samples and genotypes underwent stringent QC. Genotype data was cleaned using PLINK v1.07 (http://pngu.mgh.harvard.edu/purcell/plink/)^52^ by applying a minimum call rate for SNPs and individuals (98%) and minimum minor allele frequencies (MAF = 0.02). SNPs not in Hardy-Weinberg equilibrium (P < 1 × 10^−6^) were excluded. Gender identification was verified by analysis of X-chromosome SNPs. We tested for unanticipated duplicates and cryptic relatedness (Pihat ≥ 0.5) using pairwise genome-wide estimates of proportion identity-by-descent using PLINK v1.07 (http://pngu.mgh.harvard.edu/purcell/plink/)^52^. When a pair of identical samples or a pair of samples with cryptic relatedness was identified, the sample with a higher number of SNPs that passed QC was prioritized. EIGENSTRAT^53^ was used for each cohort separately to calculate principal component factors for each sample and confirm the ethnicity of the samples. The 1000 genomes data (June 2011 release) and BEAGLE v3.3.1^54^ were used to impute up to 6 million SNPs. SNPs with a BEAGLE R^2^ < 0.3, a minor allele frequency (MAF) <0.025, a call rate lower than 95%, a Gprobs score lower than 0.90 and those out of Hardy-Weinberg equilibrium (p < 1 × 10^−5^) were removed. After imputation, 5,815,690 SNPs passed the QC process.

### Assessment of Analyte Profiles and Quality Control

A set of 0.5 mL EDTA plasma samples from ADNI and KADRC participants was selected and shipped to Myriad Rules Based Medicine, Inc. (Myriad RBM, Austin, TX). A set of 190 protein levels from plasma for each selected individual was measured by multiplex immunoassay on the Human DiscoveryMAP panel v1.0 (https://rbm.myriad.com/products-services/humanmap-services/human-discoverymap/) using the Luminex100 platform by RBM. Samples with more than 10% of missing data across analytes were removed, then analytes were excluded if they had missing data for 10% of the samples or values were below the detection limit, in either of the studies. After the QC step, a total of 146 metabolites were included in each dataset of the present study.

### Statistical analyses

For each study, prior to the analyses, all analyte values were log-transformed, standardized so the mean for each analyte was equal to zero, and outliers were removed as previously described^12^,^51^,^55^,^56^,^57^,^58^,^59^. Log-transformed, standardized values were tested for significant deviations from a normal distribution using the Shapiro-Wilk test. We performed a single variant analysis for each analyte using PLINK v1.9 (http://pngu.mgh.harvard.edu/purcell/plink/)^52^, including age, gender, AD status, and the first 2 principal components as covariates. The significance threshold for the joint analyses was defined as p < 5.0 × 10^−8^ based on the commonly used threshold thought to be appropriate for the likely number of independent tests with Bonferroni correction. To approximate an internal replication, all SNPs that passed the genome-wide significance threshold had to pass the threshold p < 0.05 in single variant analyses of the individual datasets and had to have similar effect sizes in the same direction. To ensure that results were not confounded by AD status, single variant analyses were performed on all of the AD cases from both datasets separately from all of the controls from both datasets. All genome-wide significant SNPs from the joint analyses also had to have similar effect sizes in the same direction in the case-control stratified analyses. QQ plots were generated for each analysis to illustrate the distribution of the observed and expected p-values for all eligible SNPs^60^. Regional plots showing LD and the location of nearby genes were generated for the top ranking SNPs for each metabolite using LocusZoom v1.1, build hg19/1000 Genomes Mar 2012 EUR (http://csg.sph.umich.edu/locuszoom/)^61^. If more than one significant SNP clustered at a locus, the SNP with the smallest p-value was reported as the sentinel marker. All analyses were performed using BEAGLE v3.3.1^54^, EIGENSTRAT^53^, SAS v9.2 for Linux (copyright © 2008 by SAS Institute Inc) and PLINK v1.07 and v1.9 (http://pngu.mgh.harvard.edu/purcell/plink/)^52^ software.

### Meta-analyses

We performed the single variant analyses as described above for ADNI and KADRC separately. We used METAL (version released 2011-03-25, http://www.sph.umich.edu/csg/abecasis/Metal/index.html)^62^ to perform meta-analyses of the two datasets for each analyte by combining p-values across studies, weighting each study by its sample size.

### Conditional analyses

To identify additional independent signals in a locus we conducted conditional analyses. We performed a series of sequential conditional analyses by adding the most strongly associated SNP into the regression model as a covariate and testing all remaining regional SNPs for association. This approach was used to determine additional secondary signals and was performed by adding SNPs one at a time until no significance was seen. Consistent with the locus-specific analysis statistical significance for the conditional analysis was defined at p < 5.0 × 10^−8^.

### Annotation of GWAS hits

All significant GWAS SNPs were taken forward for functional annotation. We used SNPnexus (http://www.snp-nexus.org), build GRCh37/hg19^63^ and ANNOVAR version 2015-03-22^64^ to perform SNP annotation and to identify the putative functional SNPs. All significant GWAS SNPs were also examined for potential regulatory functions using RegulomeDB (http://regulome.stanford.edu/)^25^. We searched the National Human Genome Research Institute’s (NHGRI) catalog of genome-wide association studies to identify SNP trait associations for selected analytes.

## Additional Information

**How to cite this article**: Deming, Y. *et al.* Genetic studies of plasma analytes identify novel potential biomarkers for several complex traits. *Sci. Rep.* **6**, 18092; doi: 10.1038/srep18092 (2016).

## Supplementary Material

Supplementary Information

## Figures and Tables

**Table 1:** Characteristics of ADNI and KADRC cohorts.

	Joint	ADNI	KADRC
Samples	818	506	312
Age (y)	76.36 ± 7.66	78.30 ± 7.39	73.22 ± 7.02
Gender (M/F)	438/380	317/189	121/191
case/control	558/260	434/72	124/188
APOE4 (%)	46.69	53.16	36.22

Levels of CD40 in plasma was significantly higher in MS cases than controls (p = 0.041, beta = −1.837).

**Table 2:** Genome-wide significant results (*cis* = within 1MB of gene encoding protein).

Analyte	Chr	Position	SNP	*Gene*	Effect	Potential	MAF	Joint	beta	Previously	Reference
						function*		p-value		reported	
CFHR1	1	196698945	rs12144939	*CFH*	*cis*	missense	0.357	8.99E-143	−1.108	known	9
IL6r	1	154425456	rs12126142	*IL6R*	*cis*	missense	0.392	1.81E-106	0.850	known	6
ApoA4	11	116677723	rs1263167	*APOA4*	*cis*	intergenic	0.197	2.64E-54	−0.919	known	9
SELE	9	136149399	rs507666	*ABO*	*trans*	intronic	0.191	1.01E-52	−0.882	known	15,17
FetuinA	3	186335941	rs2070633	*AHSG*	*cis*	missense	0.324	2.88E-44	−0.629	known	9
ACE	17	61566031	rs4343	*ACE*	*cis*	synonymous	0.492	6.66E-44	0.493	known	9
THP	16	20357281	rs12934455	*UMOD*	*cis*	intronic	0.158	2.80E-42	−0.871	known	9
AGT	1	230869025	rs35837081	*AGT*	*cis*	missense	0.126	4.45E-34	0.890	known	9
IL16	15	81598269	rs11556218	*IL16*	*cis*	missense	0.076	6.02E-32	−1.064	known	9
HCC4	17	34303312	rs80329614	*CCL16*	*cis*	3′ UTR	0.074	1.16E-27	−0.958	known	9
F7	13	113752831	rs10665	*F7*	*cis*	missense	0.107	1.44E-26	−0.598	known	13
ApoE	19	45410002	rs769449	*APOE*	*cis*	synonymous	0.228	2.76E-26	−0.594	known	18
CA19-9	19	49207255	rs485073	*FUT2*	*trans*	missense	0.485	2.12E-23	−0.509	known	21
CD40	20	44730245	rs6032660	*CD40*	*cis*	5′ UTR	0.248	9.81E-21	−0.463	known	9
CEA	19	49207651	rs570794	*FUT2*	*trans*	missense	0.485	2.84E-17	−0.424	known	21,22
MIP1a	17	34391617	rs2015086	*CCL18*	*cis*	intergenic	0.152	2.56E-15	0.490	novel	
ApoH	17	64211973	rs2873966	*APOH*	*cis*	missense	0.064	3.37E-15	0.428	known	9
TF	3	133478557	rs6762415	*TF*	*cis*	intronic	0.464	1.17E-14	−0.390	known	16
MPIF1	17	34346198	rs861273	*CCL23*	*cis*	missense	0.229	6.37E-14	−0.455	known	9
HP	16	72066102	rs72787038	*HP*	*cis*	intergenic	0.186	9.69E-14	0.468	known	19
MCP2	17	32647831	rs1133763	*CCL8*	*cis*	missense	0.147	3.54E-13	−0.419	known	9
AAT	14	94813402	rs926144	*SERPINA1*	*cis*	intergenic	0.197	4.71E-12	−0.405	novel	
RAGE	6	32151443	rs2070600	*AGER*	*cis*	missense	0.036	1.86E-11	−0.814	novel	
TNC	8	27805498	rs2685421	*SCARA5*	*trans*	intronic	0.283	5.27E-11	0.358	novel	
GSTa	6	52679690	rs9395826	*GSTA1*	*cis*	intergenic	0.489	8.19E-11	−0.396	known	9
MMP7	20	14197364	rs9753755	*MACROD2*	*trans*	intronic	0.034	8.87E-11	0.913	novel	
CRP	19	45387459	rs12972156	*TOMM40*	*trans*	synonymous	0.228	9.93E-11	−0.383	known	24
ApoH	18	51648690	rs117842936	*MBD2*	*trans*	intergenic	0.055	1.03E-10	−0.727	novel	
MIP1b	3	46293070	rs145617407	*CCR3*	*trans*	intronic	0.133	2.58E-10	0.348	novel	
ApoH	9	71885730	rs148521708	*TJP2*	*trans*	intergenic	0.051	2.64E-10	−0.742	novel	
NrCAM	7	107992582	rs10487851	*NRCAM*	*cis*	intronic	0.310	3.01E-10	0.242	known	9
FGF4	4	173209932	rs13117858	*GALNTL6*	*trans*	intronic	0.141	3.12E-10	0.410	novel	
SNPs below this line have p < 5E-8 and > 3.42E-10											
CD5L	1	157804648	rs2765501	*CD5L*	*cis*	intronic	0.395	9.97E-10	0.309	known	9
LPa	6	161137990	rs783147	*LPA*	*cis*	splice donor	0.025	1.96E-09	−0.290	known	20
Sortilin	1	109818530	rs646776	*CELSR2*	*cis*	3′ UTR	0.231	2.20E-09	0.331	novel	
BLC	1	85948672	rs7541151	*DDAH1*	*trans*	intronic	0.028	6.44E-09	−0.720	novel	
TFF3	3	5791627	rs2444229	*MIR4790*	*trans*	intergenic	0.247	8.33E-09	0.311	novel	
Leptin	10	131923448	rs2031468	*GLRX3*	*trans*	intergenic	0.399	1.04E-08	−0.250	novel	
ENA78	4	74857658	rs409336	*CXCL5*	*cis*	intergenic	0.143	1.11E-08	0.405	novel	
MIP1b	17	34819191	rs4796217	*CCL4L2*	*cis*	intergenic	0.324	1.19E-08	−0.234	known	6
IL13	3	23128588	rs7433647	*UBE2E2*	*trans*	intergenic	0.125	1.21E-08	−0.438	novel	
CystC	20	23633755	rs13039144	*CST3*	*cis*	intergenic	0.147	1.23E-08	−0.347	known	9,14,23
MPIF1	5	30117148	rs72752381	*CDH6*	*trans*	intergenic	0.031	1.37E-08	0.767	novel	
RAGE	2	49180132	rs4953649	*FSHR*	*trans*	intronic	0.347	1.66E-08	0.288	novel	
ACE	9	136141870	rs2519093	*ABO*	*trans*	missense	0.072	1.90E-08	−0.352	**known	**^26^
MCSF	5	10906122	rs73741236	*CTNND2*	*trans*	intergenic	0.028	2.08E-08	−0.841	novel	
GROa	7	157469995	rs1263549	*PTPRN2*	*trans*	intronic	0.039	2.16E-08	0.685	novel	
ANG2	22	44588459	rs3747214	*PARVG*	*trans*	intronic	0.411	2.21E-08	0.277	novel	
TECK	18	55016922	rs72927542	*ST8SIA3*	*trans*	intergenic	0.038	2.26E-08	0.679	novel	
MCSF	8	21133875	rs111494896	*GFRA2*	*trans*	intergenic	0.041	2.77E-08	−0.695	novel	
IL18	13	61673417	rs146245376	*MIR3169*	*trans*	intergenic	0.062	3.06E-08	−0.559	novel	
F7	10	122564938	rs11594693	*WDR11*	*trans*	intronic	0.265	3.72E-08	0.239	novel	
THPO	17	41570427	rs2279191	*DHX8*	*trans*	intronic	0.242	3.83E-08	0.239	novel	
CEA	2	100278651	rs12468845	*AFF3*	*trans*	intronic	0.036	3.88E-08	−0.834	novel	
VCAM1	2	238834521	rs13027473	*RAMP1*	*trans*	intergenic	0.149	4.53E-08	−0.345	novel	
IL8	2	9893150	rs11889675	*TAF1B*	*trans*	intergenic	0.152	4.60E-08	0.364	novel	

Chr = chromosome. *Potential function of an associated variant within the locus, not necessarily the marker SNP. **Previously reported to be associated with ACE activity.

**Table 3:** Plasma analyte levels associated with multiple loci.

Analyte	Chr	Position	SNP	*Gene*	Conditioned	Original		Conditional		LD with reference	
					SNP	p-value	beta	p-value	beta	r^2^	D'
ApoH	17	64211973	rs2873966	*APOH*	NA - reference SNP	3.37E-15	0.428		reference SNP	1.000	1.000
		64202857	rs17690171	*APOH*	rs2873966	1.02E-15	−0.511	5.53E-09	−0.389	0.104	0.981
		64223183	rs8178828	*APOH*	rs2873966, rs17690171	6.84E-13	−0.714	6.64E-13	−0.700	0.030	1.000
CA19-9	19	49207255	rs485073	*FUT2*	NA - reference SNP	2.12E-23	−0.509	reference SNP		1.000	1.000
		5831724	rs112313064	*FUT6*	rs485073	7.46E-23	−0.507	3.79E-26	−0.509	0.000	0.012
		5851801	rs2306969	*FUT3*	rs485073, rs112313064	6.11E-23	−0.575	2.78E-09	−0.378	0.000	0.020
FetuinA	3	186335941	rs2070633	*AHSG*	NA - reference SNP	2.88E-44	−0.629	reference SNP		1.000	1.000
		186332571	rs2593813	*AHSG*	rs2070633	6.74E-44	−0.698	2.94E-10	−0.408	0.471	0.918
IL6r	1	154425456	rs12126142	*IL6R*	NA - reference SNP	1.81E-106	0.850	reference SNP		1.000	1.000
		154425135	rs7526131	*IL6R*	rs12126142	4.47E-72	−0.711	1.43E-10	−0.276	0.452	0.971
LPa	6	161137990	rs783147	*PLG*	NA - reference SNP	1.96E-09	−0.290	reference SNP		1.000	1.000
		160551093	rs4646272	*SLC22A1*	rs783147	9.86E-09	0.590	1.64E-09	0.607	0.002	0.149

Chr = chromosome, LD = linkage disequilibrium.

**Table 4:** Potential pleiotropic associations.

Analyte	SNP	Gene	Chr	Position	Joint p-value	Correlation of analyte levels
SELE	rs2519093	*ABO*	9	136141870	7.62E-51	ACE/SELE: r = 0.118, p = 7.17E-4
ACE	rs2519093	*ABO*	9	136141870	1.90E-08	vWF/ACE: r = –0.059, p = 0.09
vWF	rs687289	*ABO*	9	136137106	8.87E-08	SELE/vWF: r = –0.097, p = 5.61E-3
CA19-9	rs485073	*FUT2*	19	49207255	2.12E-23	r = 0.166, p = 2.98E-6
CEA	rs485073	*FUT2*	19	49207255	4.07E-16	
ApoE	rs769449	*APOE*	19	45410002	2.76E-26	r = 0.204, p = 4.82E-9
CRP	rs769449	*APOE*	19	45410002	6.69E-09	

Chr = chromosome.

**Table 5:** Joint GWAS top SNPs/genes related to disease based on NHGRI catalog.

Analyte	SNP	Gene	Predicted	Joint	Other traits associated with loci or gene
			function	p-value	(based on NHGRI catalog)
AAT	rs926144	*SERPINA6*	intergenic	4.71E-12	Breast size
ACE	rs4343	*ACE*	coding	6.66E-44	Metabolite levels; Metabolic traits; Angiotensin-converting enzyme activity
ACE	rs2519093	*ABO*	intronic	1.90E-08	Lipid traits; Coronary artery disease; Ischemic stroke; Large artery stroke; Serum alkaline phosphatase levels; Malaria; Venous thromboembolism; Graves’ disease; Thyroid hormone levels; Tumor biomarkers; End-stage coagulation; Coagulation factor levels; Red blood cell traits; Obesity-related traits; Activated partial thromboplastin time; Duodenal ulcer; Inflammatory biomarkers; Liver enzyme levels; Metabolic traits; Soluble ICAM-1; D-dimer levels; Phytosterol levels; E-selectin levels; Soluble levels of adhesion molecules; Hematological and biochemical traits; mean corpuscular hemoglobin concentration; Angiotensin-converting enzyme activity; Pancreatic cancer; vWF and FVIII levels
AGT	rs35837081	*AGT*	intergenic	4.45E-34	AGT levels
ANG2	rs3747214	*PARVG*	intronic	2.21E-08	—
ApoA4	rs1263167	*APOA4*	intergenic	2.64E-54	Hypertriglyceridemia; Coronary heart disease; HDL cholesterol; LDL cholesterol; Triglycerides; total cholesterol; Hematological and biochemical traits
ApoE	rs769449	*APOE*	intronic	2.76E-26	LDL cholesterol; Alzheimer’s disease; HDL cholesterol; C-reactive protein; Age-related macular degeneration; Cholesterol, total; Alzheimer’s disease biomarkers; Brain imaging; Triglycerides; Quantitative traits; Apolipoprotein Levels; Metabolite levels; Cardiovascular disease risk factors; Lipid traits; Response to statin therapy (LDL-C); Lipid metabolism phenotypes
ApoH	rs2873966	*APOH*	intronic	3.37E-15	Blood pressure measurement (high sodium and potassium intervention); LDL cholesterol; B2-Glycoprotein I plasma levels
ApoH	rs17690171	*APOH*	intergenic	1.02E-15	Blood pressure measurement (high sodium and potassium intervention); LDL cholesterol; B2-Glycoprotein I plasma levels
ApoH	rs8178828	*APOH*	intronic	6.84E-13	Blood pressure measurement (high sodium and potassium intervention); LDL cholesterol; B2-Glycoprotein I plasma levels
ApoH	rs117842936	*MBD2*	intergenic	1.03E-10	Periodontitis
ApoH	rs148521708	*TJP2*	intergenic	2.64E-10	Refractive error; Renal sinus fat
BLC	rs7541151	*DDAH1*	intronic	6.44E-09	Serum dimethylarginine levels; multiple sclerosis
CA19-9	rs485073	*FUT2*	3′ UTR	2.12E-23	Tumor biomarkers; Vitamin B12 levels; Bipolar disorder; Retinal vascular caliber; Liver enzyme levels (alkaline phosphatase); Crohn’s disease; Cholesterol, total; Metabolic traits; Obesity-related traits; Liver enzyme levels (gamma-glutamyl transferase); Folate pathway vitamin levels; Homocysteine levels
CA19-9	rs112313064	*FUT6*	coding	7.46E-23	Tumor biomarkers; Vitamin B12 levels; N-glycan levels
CA19-9	rs2306969	*FUT3*	upstream 2KB	6.11E-23	Elevated serum carcinoembryonic antigen levels; N-glycan levels
CD40	rs6032660	*CD40*	intergenic	9.81E-21	Inflammatory bowel disease; Kawasaki disease; Rheumatoid arthritis; multiple sclerosis
CD5L	rs2765501	*CD5L*	intronic	9.97E-10	CD6; CD5; PTGDR2
CEA	rs570794	*FUT2*	3′ UTR	2.84E-17	Tumor biomarkers; Vitamin B12 levels; Bipolar disorder; Retinal vascular caliber; Liver enzyme levels (alkaline phosphatase); Crohn’s disease; Cholesterol, total; Metabolic traits; Obesity-related traits; Liver enzyme levels (gamma-glutamyl transferase); Folate pathway vitamin levels; Homocysteine levels
CEA	rs12468845	*AFF3*	intronic	3.88E-08	—
CFHR1	rs12144939	*CFH*	intronic	8.99E-143	Age-related macular degeneration; Circulating myeloperoxidase levels (serum); Complement C3 and C4 levels; Nephropathy Meningococcal disease
CRP	rs12972156	*PVRL2*	intronic	9.93E-11	Alzheimer’s disease biomarkers; Age-related macular degeneration; HDL cholesterol; Alzheimer’s disease
CystC	rs13039144	*CST3*	intergenic	1.23E-08	Chronic kidney disease; Cystatin C
ENA78	rs409336	*CXCL5*	intergenic	1.11E-08	Inflammatory bowel disease; Metabolite levels
F7	rs10665	*MCF2L*	3′ UTR	1.44E-26	Osteoarthritis; Factor VII
F7	rs11594693	*WDR11*	intronic	3.72E-08	—
FetuinA	rs2070633	*AHSG*	intronic	2.88E-44	Fetuin-A levels; Activated partial thromboplastin time
FetuinA	rs2593813	*AHSG*	intronic	6.74E-44	Fetuin-A levels; Activated partial thromboplastin time
FGF4	rs13117858	*GALNTL6*	intronic	3.12E-10	—
GROa	rs1263549	*PTPRN2*	intronic	2.16E-08	Myopia (pathological); Response to amphetamines; Bipolar disorder and schizophrenia; Obesity-related traits
GSTa	rs9395826	*GSTA1*	intergenic	8.19E-11	GSTa levels
HCC4	rs80329614	*CCL16*	downstream 500B	1.16E-27	HCC4 levels
HP	rs72787038	*DHODH*	intergenic	9.69E-14	Attention deficit hyperactivity disorder and conduct disorder
IL13	rs7433647	*UBE2E2*	intergenic	1.21E-08	Psychosis (atypical); Type 2 diabetes
IL16	rs11556218	*IL16*	missense	6.02E-32	Inattentive symptoms
IL18	rs146245376	*MIR3169*	intergenic	3.06E-08	—
IL6r	rs12126142	*IL6R*	intronic	1.81E-106	IL6r levels; Asthma; C-reactive protein; Protein quantitative trait loci; Pulmonary function; Fibrinogen
IL6r	rs7526131	*IL6R*	intronic	4.47E-72	IL6r levels; Asthma; C-reactive protein; Protein quantitative trait loci; Pulmonary function; Fibrinogen
IL8	rs11889675	*TAF1B*	intergenic	4.60E-08	—
Leptin	rs2031468	*GLRX3*	intergenic	1.04E-08	HIV-1 susceptibility
LPa	rs783147	*PLG*	intronic	1.96E-09	Aging; Lp(a) levels
LPa	rs4646272	*SLC22A1*	intronic	9.86E-09	—
MCP2	rs1133763	*CCL8*	missense	3.54E-13	—
MCSF	rs73741236	*CTNND2*	intergenic	2.08E-08	Amyotrophic lateral sclerosis (sporadic); Myopia (pathological)
MCSF	rs111494896	*GFRA2*	intergenic	2.77E-08	Neuropathic pain in type 2 diabetes; Migraine with aura
MIP1a	rs2015086	*CCL18*	upstream 2KB	2.56E-15	Higher macrophage expression of CCL18 in human carotid atherosclerotic plaques
MIP1b	rs145617407	*CCR3*	intronic	2.58E-10	MCP1 levels; Obesity-related traits; Monocyte chemoattractant protein-1; Celiac disease
MIP1b	rs4796217	*CCL4L2*	intergenic	1.19E-08	MIP1b levels
MMP7	rs9753755	*MACROD2*	intronic	8.87E-11	Eating disorders; Brain connectivity; Presence of antiphospholipid antibodies; Hypertension; Obesity-related traits; Non-alcoholic fatty liver disease histology (other); Autism
MPIF1	rs861273	*CCL23*	upstream 2KB	6.37E-14	MPIF1 levels; Pulmonary function
MPIF1	rs72752381	*CDH6*	intergenic	1.37E-08	Response to methotrexate in juvenile idiopathic arthritis; Liver enzyme levels (gamma-glutamyl transferase); Emphysema-related traits
NrCAM	rs10487851	*NRCAM*	intronic	3.01E-10	NRCAM levels; Femoral neck bone geometry and menarche (age at onset); Coffee consumption
RAGE	rs2070600	*AGER*	missense	1.86E-11	Normal glucose metabolism; Impaired glucose metabolism; Type 2 diabetes mellitus; Chronic obstructive pulmonary disease; Prostate cancer; Pulmonary function
RAGE	rs4953649	*FSHR*	intronic	1.66E-08	Response to anti-retroviral therapy (ddI/d4T) in HIV-1 infection (Grade 3 peripheral neuropathy); Adverse response to chemotherapy (neutropenia/leucopenia) (etoposide); Polycystic ovary syndrome; Erectile dysfunction and prostate cancer treatment, Radiation response
SELE	rs507666	*ABO*	intronic	1.01E-52	Lipid traits; Coronary artery disease; Ischemic stroke; Large artery stroke; Serum alkaline phosphatase levels; Malaria; Venous thromboembolism; Graves’ disease; Thyroid hormone levels; Tumor biomarkers; End-stage coagulation; Coagulation factor levels; Red blood cell traits; Obesity-related traits; Activated partial thromboplastin time; Duodenal ulcer; Inflammatory biomarkers; Liver enzyme levels; Metabolic traits; Soluble ICAM-1; D-dimer levels; Phytosterol levels; E-selectin levels; Soluble levels of adhesion molecules; Hematological and biochemical traits; mean corpuscular hemoglobin concentration; Angiotensin-converting enzyme activity; Pancreatic cancer; vWF and FVIII levels
Sortilin	rs646776	*CELSR2*	downstream 500B	2.20E-09	Coronary heart disease; LDL cholesterol; Metabolite levels; Cholesterol, total; Progranulin levels; Response to statin therapy; Lipid metabolism phenotypes; Myocardial infarction (early onset); Cardiovascular disease risk factors; Lipoprotein-associated phospholipase A2 activity and mass
TECK	rs72927542	*ST8SIA3*	intergenic	2.26E-08	—
TF	rs6762415	*TF*	intronic	1.17E-14	Transferrin levels; Alcohol consumption (transferrin glycosylation); Iron status biomarkers; Hepcidin levels; Iron levels
TFF3	rs2444229	*MIR4790*	intergenic	8.33E-09	—
THP	rs12934455	*UMOD*	intronic	2.80E-42	Femoral neck bone geometry; Hypertension; Chronic kidney disease and serum creatinine levels; Chronic kidney disease; Renal function and chronic kidney disease
THPO	rs2279191	*DHX8*	intronic	3.83E-08	—
TNC	rs2685421	*SCARA5*	intronic	5.27E-11	Adverse response to chemotherapy (neutropenia/leucopenia) (cisplatin)
VCAM1	rs13027473	*RAMP1*	intergenic	4.53E-08	Obesity-related traits
