# Supplementary material for: Genetic studies of plasma analytes identify novel potential biomarkers for several complex traits
Source: Sci Rep. 2016 Jan 4;6:18092. doi: 10.1038/srep18092 (PMC4698720; doi:10.1038/srep18092)
Supplement: Supplementary Information [file srep18092-s1.pdf]

**Genetic studies of plasma analytes identify novel potential biomarkers for several complex traits**

Yuetiva Deming[1,†], Jian Xia[1,2,†], Yefei Cai[1], Jenny Lord[1,3], Jorge L. Del-Aguila[1], Victoria Fernandez[1], David Carrell[1], Kathleen Black[1], John Budde[1], ShengMei Ma[1], Benjamin Saef[1], Bill Howells[1], Sarah Bertelsen[1], Alzheimer's Disease Neuroimaging Initiative (ADNI[¶]), Matthew Bailey[4], Perry G. Ridge[4], David Holtzman[5,6,7,8], John C. Morris[5,6,7,8], Kelly Bales[9], Eve H. Pickering[9], Jin-Moo Lee[5], Laura Heitsch[5], John Kauwe[4], Alison Goate[1,7,8], Laura Piccio[5], and Carlos Cruchaga[1,8,*]

1. Department of Psychiatry, Washington University School of Medicine, 660 S. Euclid Ave. B8134, St. Louis, MO 63110, USA.
2. Department of Neurology, Xiangya Hospital, Central South University, Changsha, Hunan 410008, P.R. China
3. Human Genetics Programme, Wellcome Trust Sanger Institute, Cambridge, CB10 1SA, UK
4. Department of Biology, Brigham Young University, Provo, UT, USA,
5. Department of Neurology, Washington University School of Medicine, 660 S. Euclid Ave., St. Louis, MO 63110, USA
6. Department of Developmental Biology, Washington University School of Medicine, 660 S. Euclid Ave., St. Louis, MO 63110, USA
7. Knight Alzheimer's Disease Research Center, Washington University School of Medicine, 4488 Forest Park Ave., St Louis, MO 63108, USA
8. Hope Center for Neurological Disorders. Washington University School of Medicine, 660 S. Euclid Ave. B8111, St. Louis, MO 63110, USA.
9. Neuroscience Research Unit, Worldwide Research and Development, Pfizer, Inc., Groton, CT, USA

[†]These authors equally contributed to this work.

[*]To whom correspondence should be addressed at: Department of Psychiatry, Washington University School of Medicine, 660 South Euclid Avenue B8134, St. Louis, MO 63110. E-mail: cruchagc@psychiatry.wustl.edu, tel. 314-286-0546, fax. 314-362-2244

[¶]Data used in preparation of this article were obtained from the Alzheimer's Disease Neuroimaging Initiative (ADNI) database (adni.loni.usc.edu). As such, the investigators within the ADNI contributed to the design and implementation of ADNI and/or provided data but did not participate in analysis or writing of this report. A complete listing of ADNI investigators can be found at: http://adni.loni.usc.edu/wp-content/uploads/how_to_apply/ADNI_Acknowledgement_List.pdf

# Supplementary Materials (Figures and Tables)

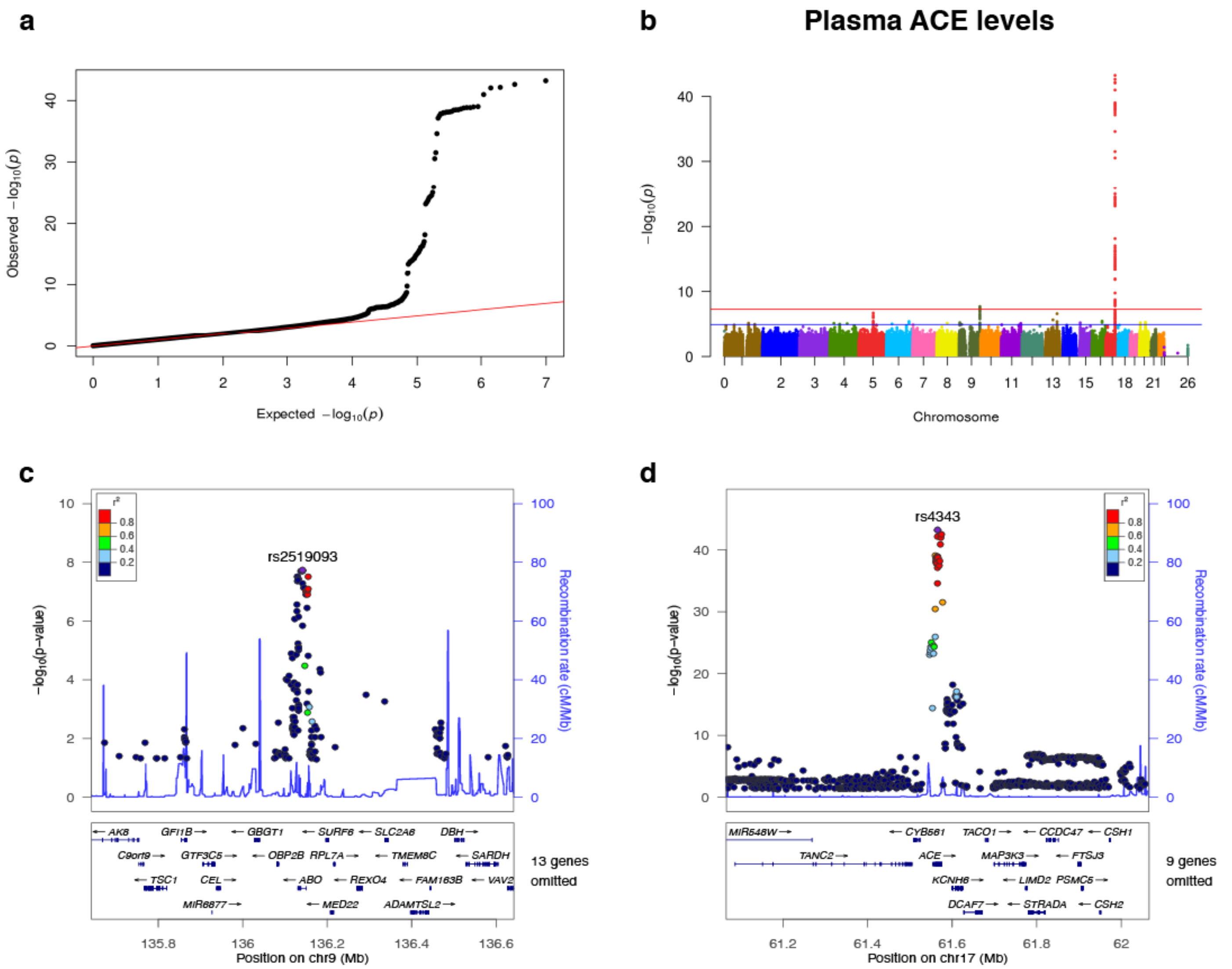


**Supplementary Figure S1. QQ, Manhattan, and regional plots for associations with plasma levels of ACE.** a) Q-Q plot of -$\log_{10}$ p-values, expected versus observed, from joint GWAS of plasma ACE levels; b) Manhattan plot of -$\log_{10}$ p-values for association with plasma levels of ACE; c) Regional plot for genome-wide significant association on chromosome 9 with ACE plasma levels; d) Regional plot for genome-wide significant association on chromosome 17 with ACE plasma levels.

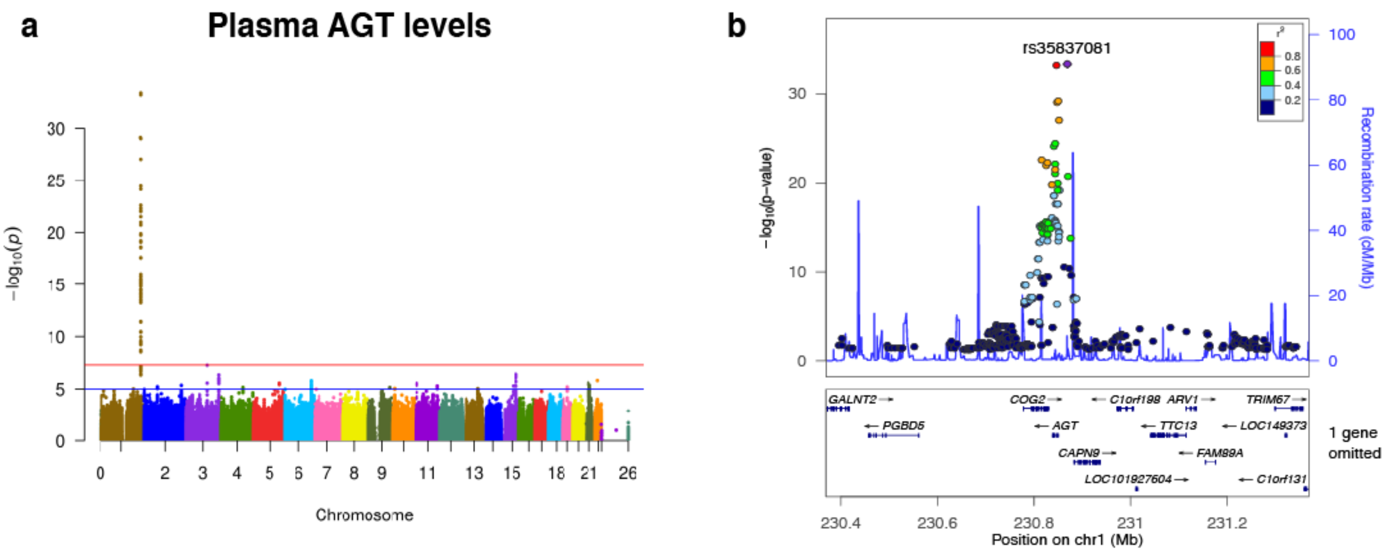


**Supplementary Figure S2. Manhattan and regional plots for associations with plasma levels of AGT.** a) Manhattan plot of $-\log_{10}$ p-values for association with plasma levels of AGT; b) Regional plot for genome-wide significant association with AGT plasma levels.

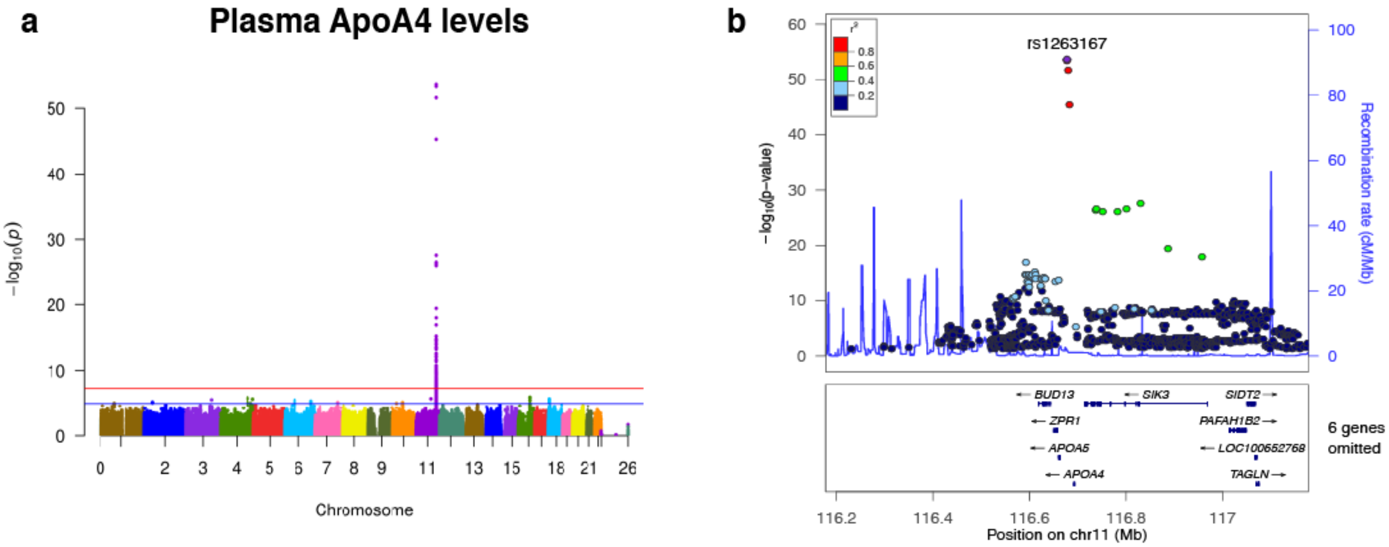


**Supplementary Figure S3. Manhattan and regional plots for associations with plasma levels of ApoA4.** a) Manhattan plot of -$\log_{10}$ p-values for association with plasma levels of ApoA4; b) Regional plot for genome-wide significant association with ApoA4 plasma levels.

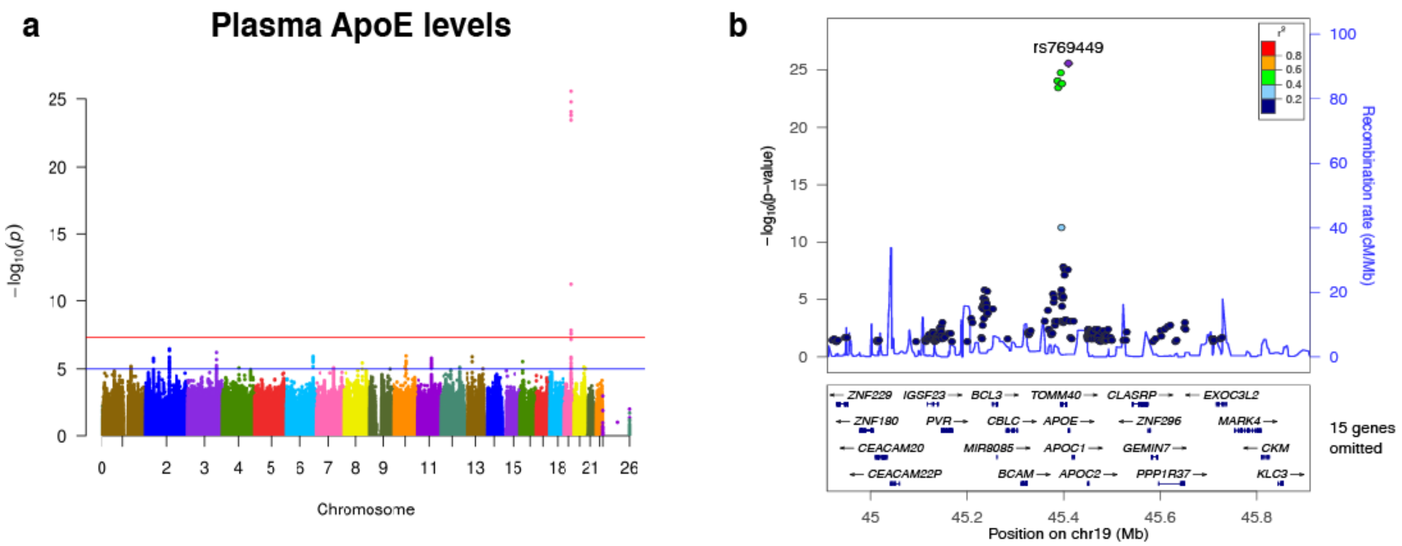


**Supplementary Figure S4. Manhattan and regional plots for associations with plasma levels of ApoE.** a) Manhattan plot of -log$_{10}$ p-values for association with plasma levels of ApoE; b) Regional plot for genome-wide significant association with ApoE plasma levels.

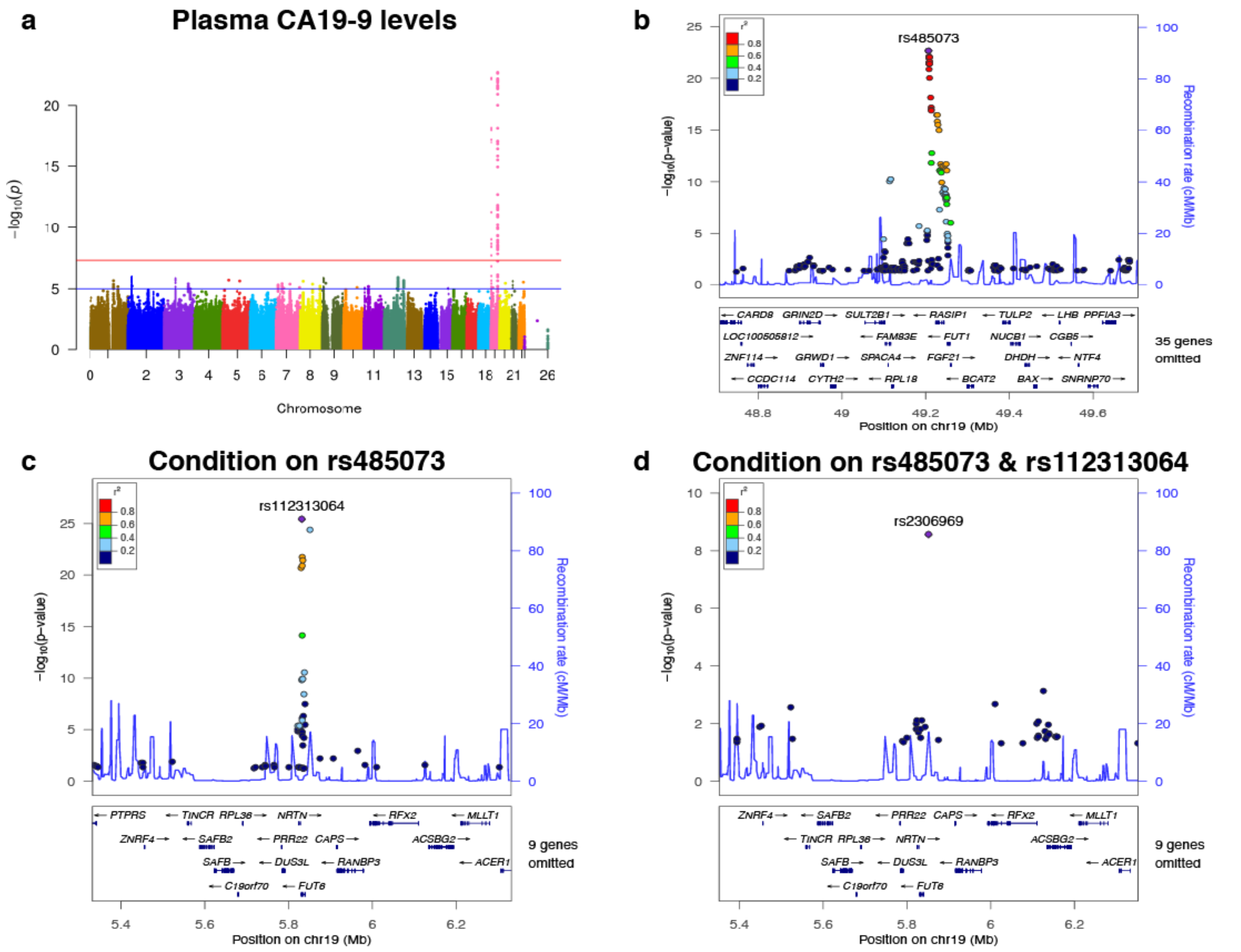


**Supplementary Figure S5. Manhattan and regional plots for associations with plasma levels of CA19-9.** a) Manhattan plot of -log$_{10}$ p-values for association with plasma levels of CA19-9; b) Regional plot for genome-wide significant association with plasma levels of CA19-9; c) Regional plot for genome-wide significant association with CA19-9 plasma levels after conditioning for rs485073; d) Regional plot for genome-wide significant association with CA19-9 plasma levels after conditioning for rs485073 and rs112313064.

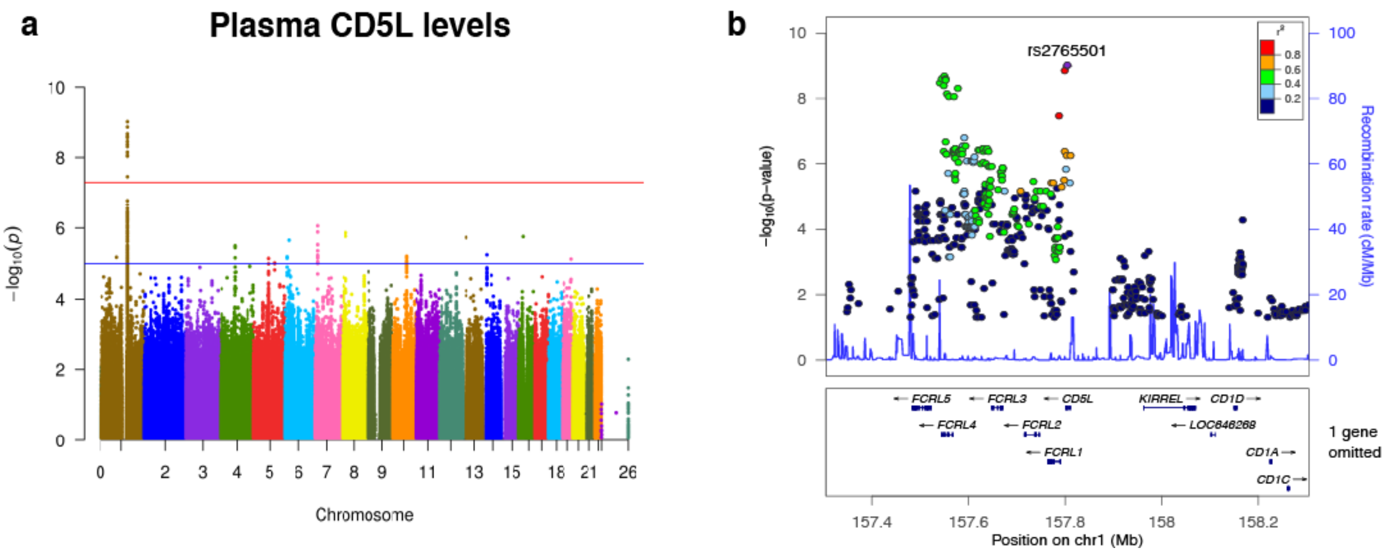


**Supplementary Figure S6. Manhattan and regional plots for associations with plasma levels of CD5L.** a) Manhattan plot of $-\log_{10}$ p-values for association with plasma levels of CD5L; b) Regional plot for genome-wide significant association with CD5L plasma levels.

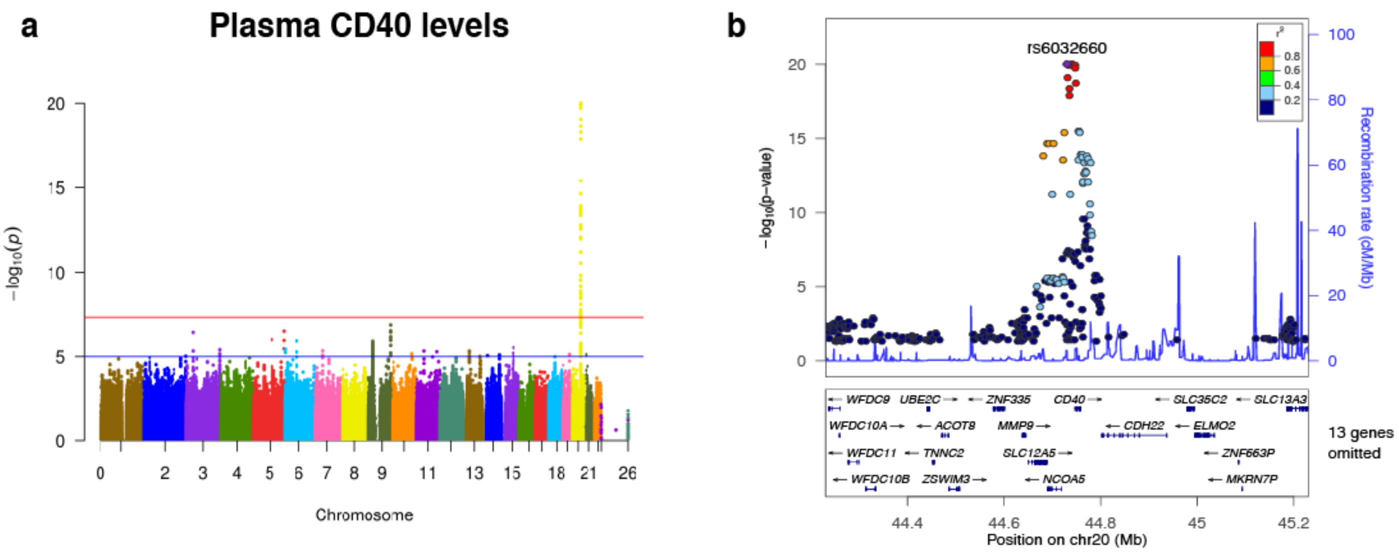


**Supplementary Figure S7. Manhattan and regional plots for associations with plasma levels of CD40.** a) Manhattan plot of -$\log_{10}$ p-values for association with plasma levels of CD40; b) Regional plot for genome-wide significant association with CD40 plasma levels.

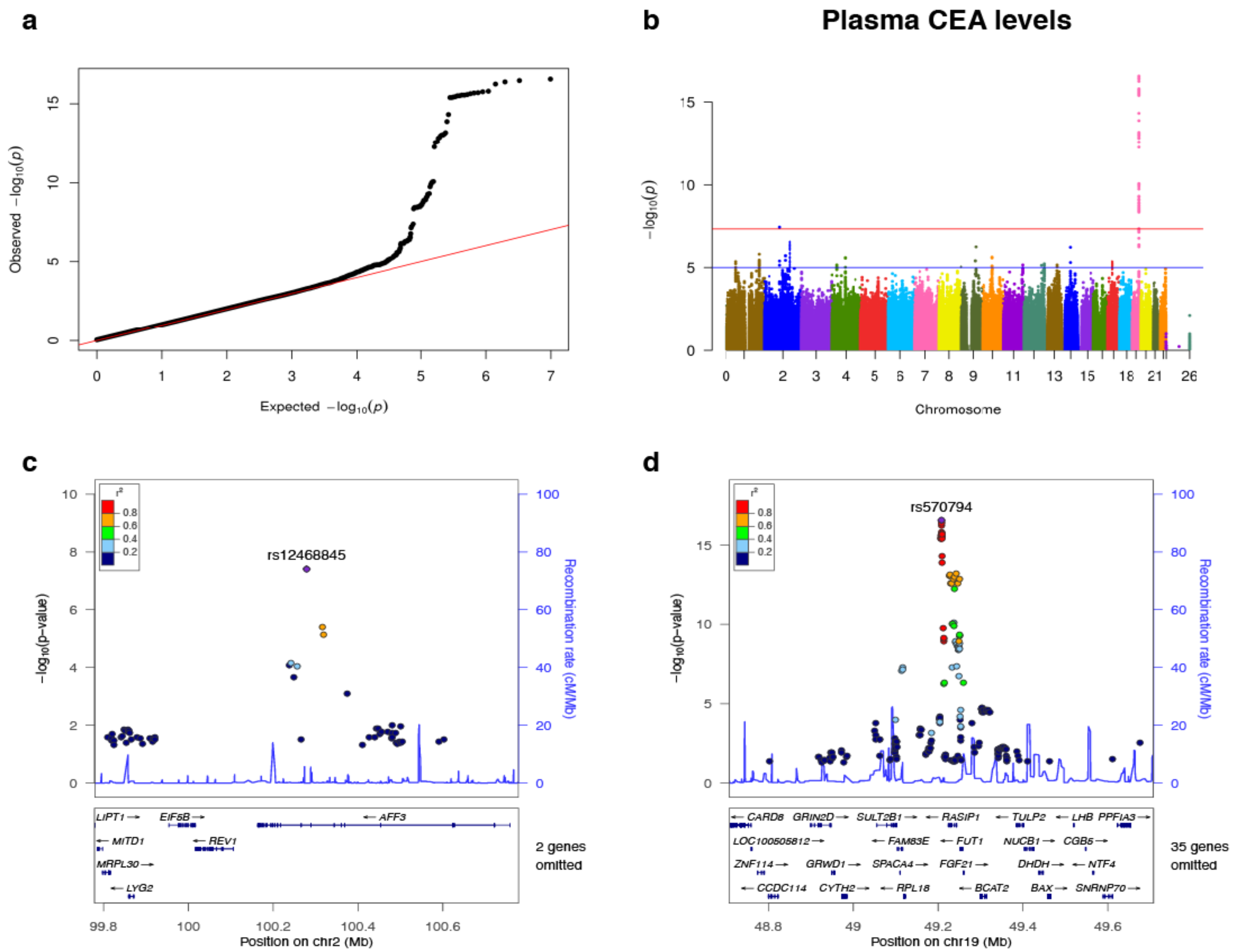


**Supplementary Figure S8. Q-Q, Manhattan and regional plots for associations with plasma levels of CEA.** a) Q-Q plot of -$\log_{10}$ p-values, expected versus observed, from joint GWAS of plasma CEA levels; b) Manhattan plot of -$\log_{10}$ p-values for association with plasma levels of CEA; c) Regional plot for genome-wide significant association on chromosome 2 with CEA plasma levels; d) Regional plot for genome-wide significant association on chromosome 19 with CEA plasma levels.

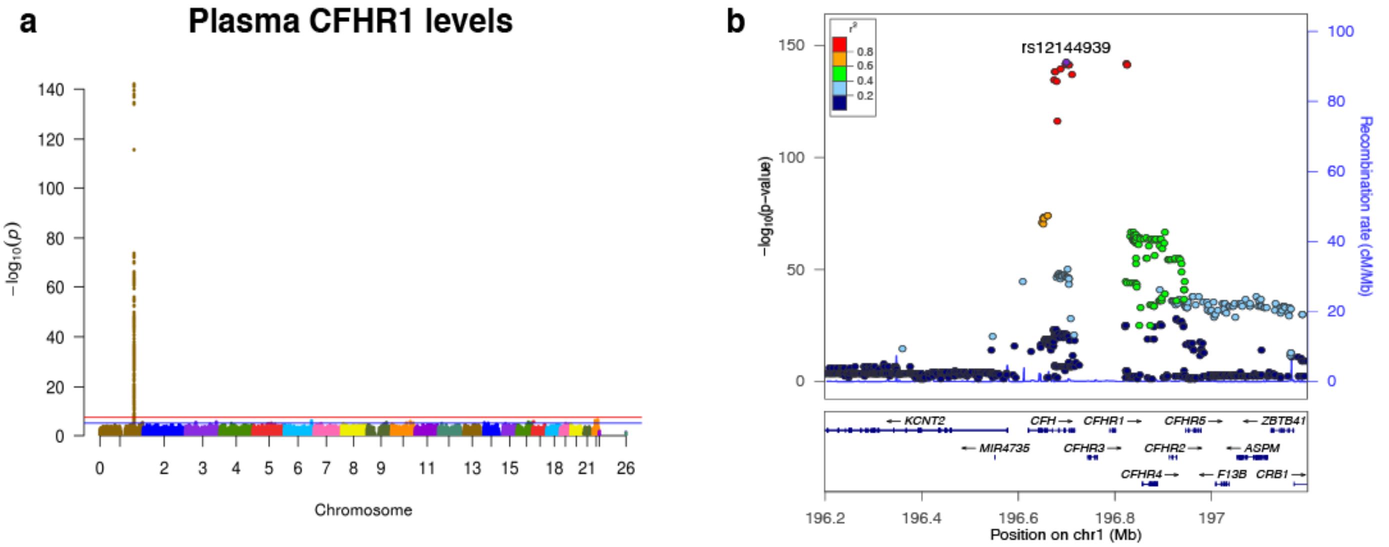


**Supplementary Figure S9. Manhattan and regional plots for associations with plasma levels of CFHR1.** a) Manhattan plot of -$\log_{10}$ p-values for association with plasma levels of CFHR1; b) Regional plot for genome-wide significant association with CFHR1 plasma levels.

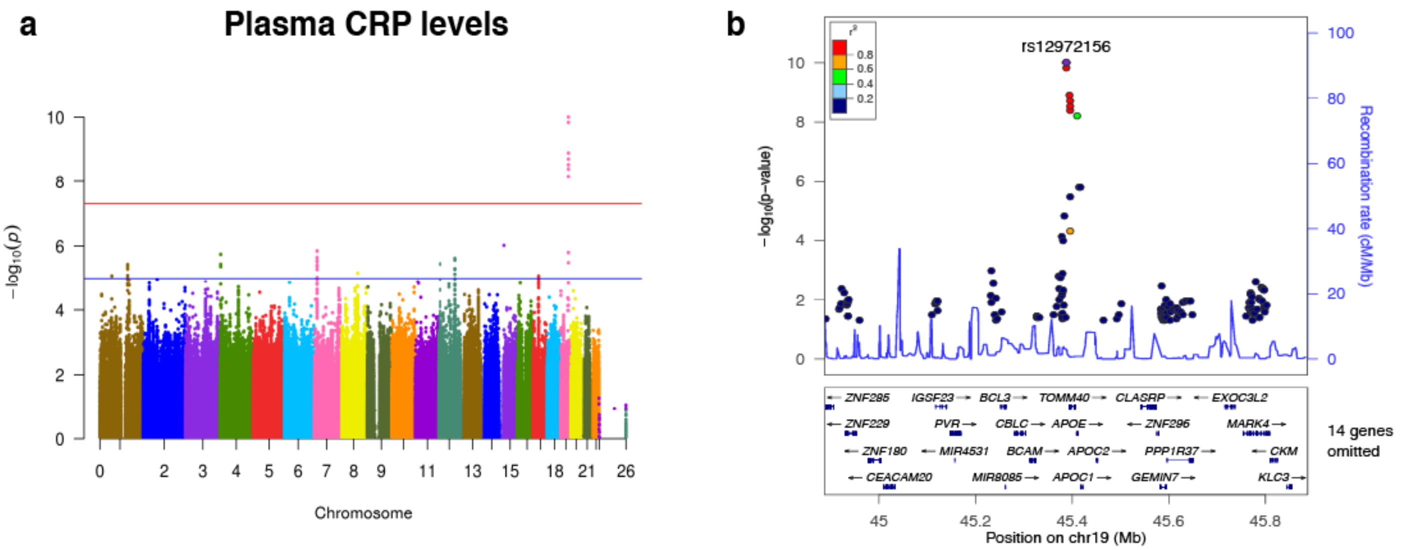


**Supplementary Figure S10. Manhattan and regional plots for associations with plasma levels of CRP.** a) Manhattan plot of -$\log_{10}$ p-values for association with plasma levels of CRP; b) Regional plot for genome-wide significant association with CRP plasma levels.

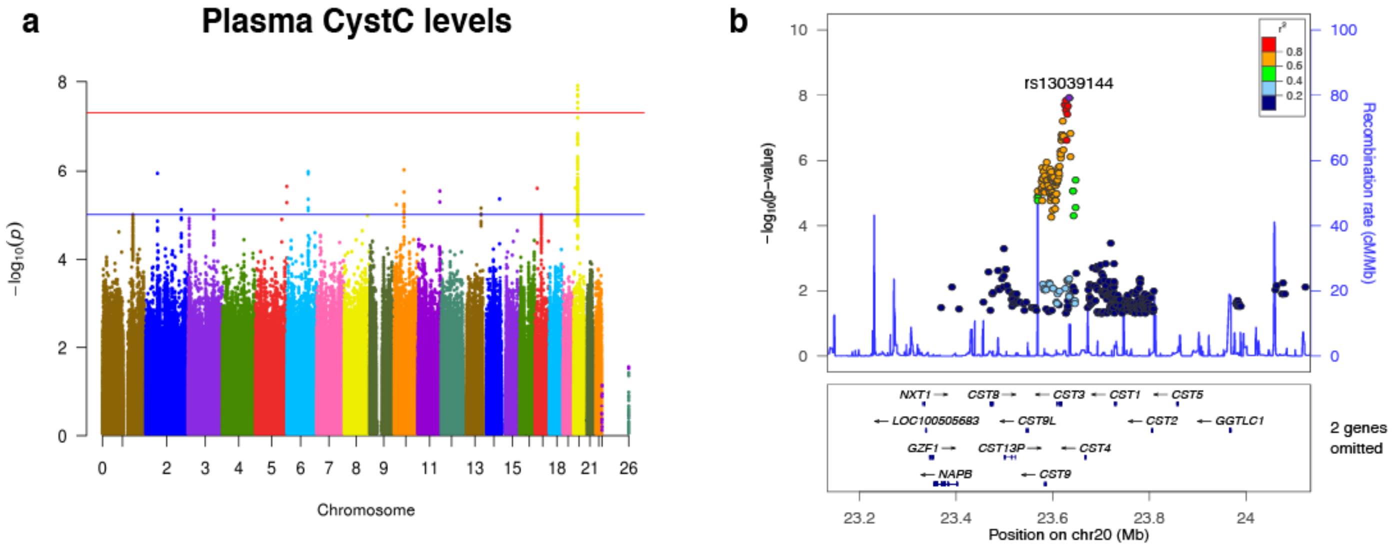


**Supplementary Figure S11. Manhattan and regional plots for associations with plasma levels of CystC.** a) Manhattan plot of -$\log_{10}$ p-values for association with plasma levels of CystC; b) Regional plot for genome-wide significant association with CystC plasma levels.

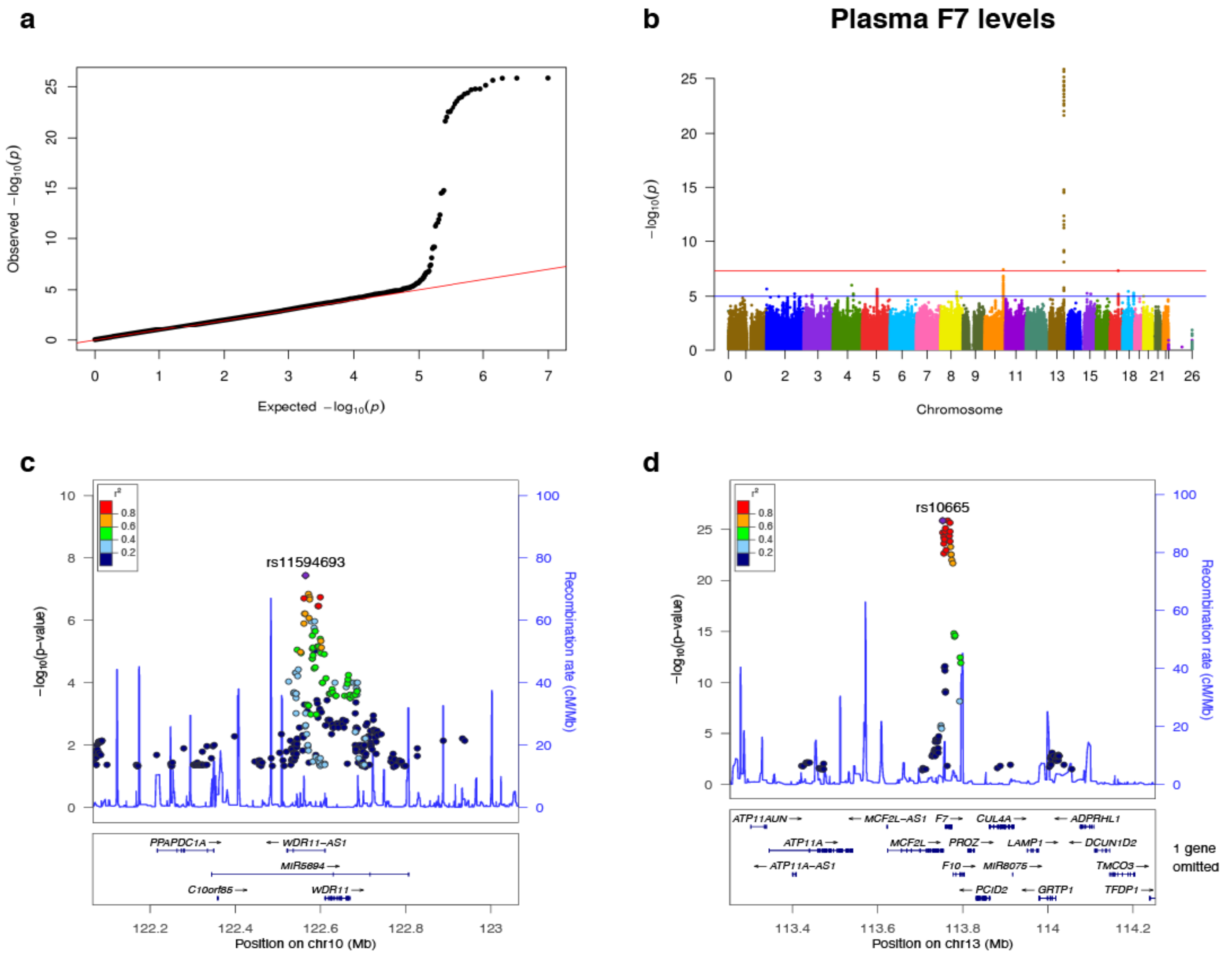


**Supplementary Figure S12. QQ, Manhattan and regional plots for associations with plasma levels of F7.** a) Q-Q plot of -$\log_{10}$ p-values, expected versus observed, from joint GWAS of plasma F7 levels; b) Manhattan plot of -$\log_{10}$ p-values for association with plasma levels of F7; c) Regional plot for genome-wide significant association on chromosome 10 with F7 plasma levels; d) Regional plot for genome-wide significant association on chromosome 13 with F7 plasma levels.

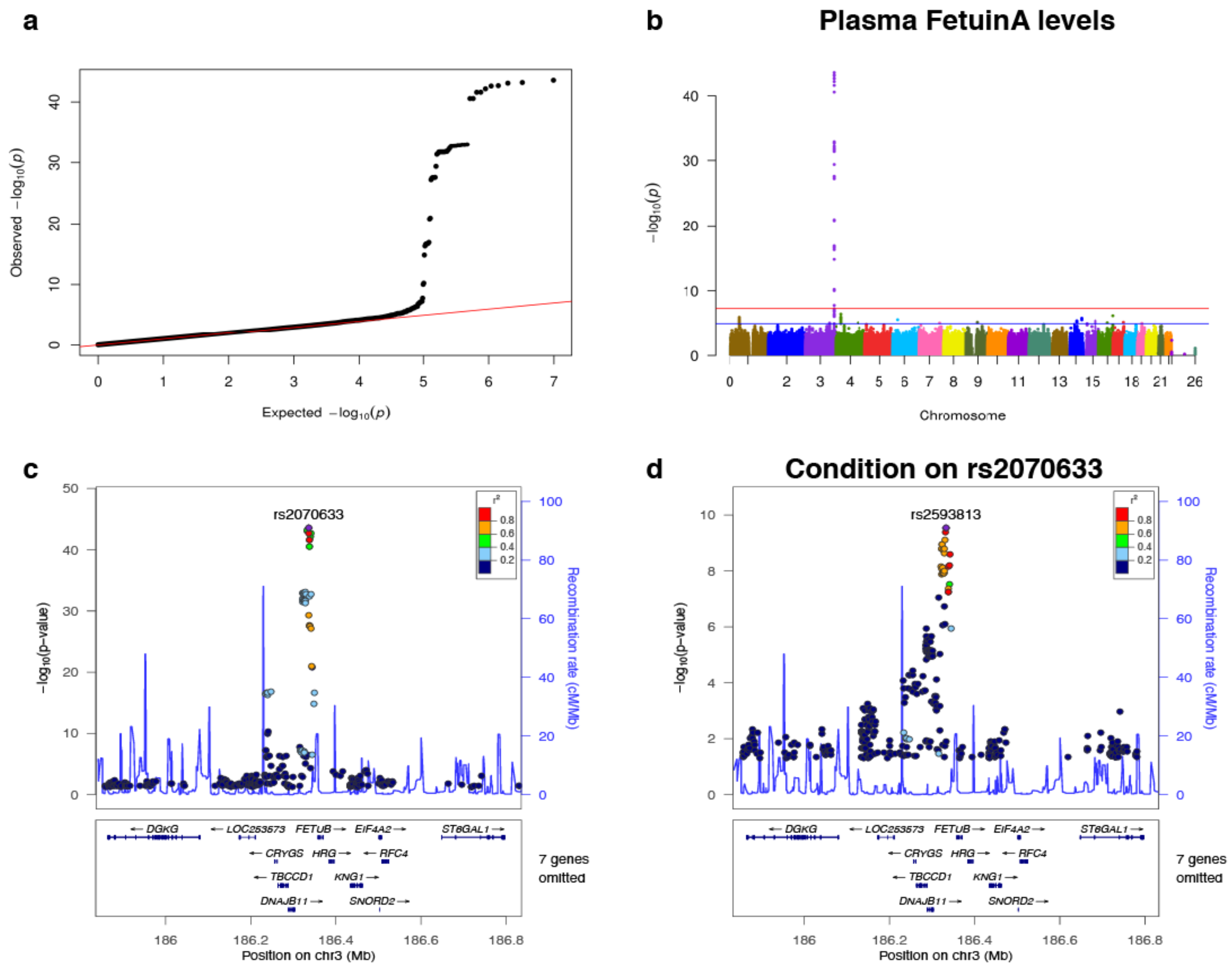


**Supplementary Figure S13. Q-Q, Manhattan and regional plots for associations with plasma levels of FetuinA.** a) Q-Q plot of -log$_{10}$ p-values, expected versus observed, from joint GWAS of plasma FetuinA levels; b) Manhattan plot of -log$_{10}$ p-values for association with plasma levels of FetuinA; c) Regional plot for genome-wide significant association with FetuinA plasma levels; d) Regional plot for genome-wide significant association with FetuinA plasma levels after conditioning on rs2070633.

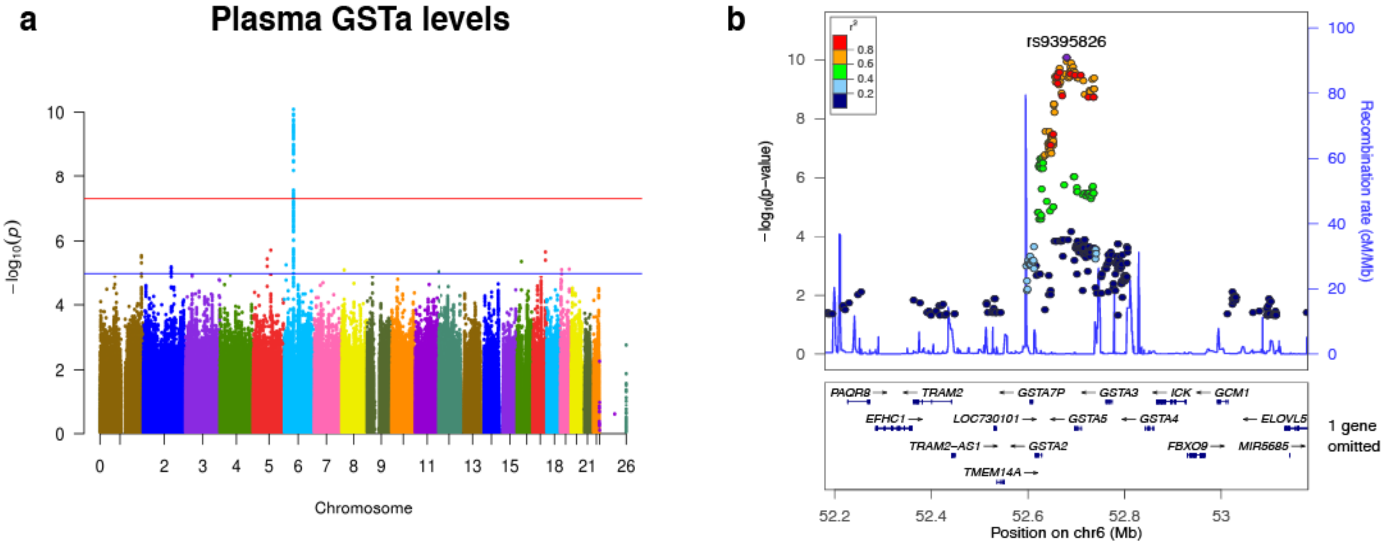


**Supplementary Figure S14. Manhattan and regional plots for associations with plasma levels of GSTa.** a) Manhattan plot of $-\log_{10}$ p-values for association with plasma levels of GSTa; b) Regional plot for genome-wide significant association with GSTa plasma levels.

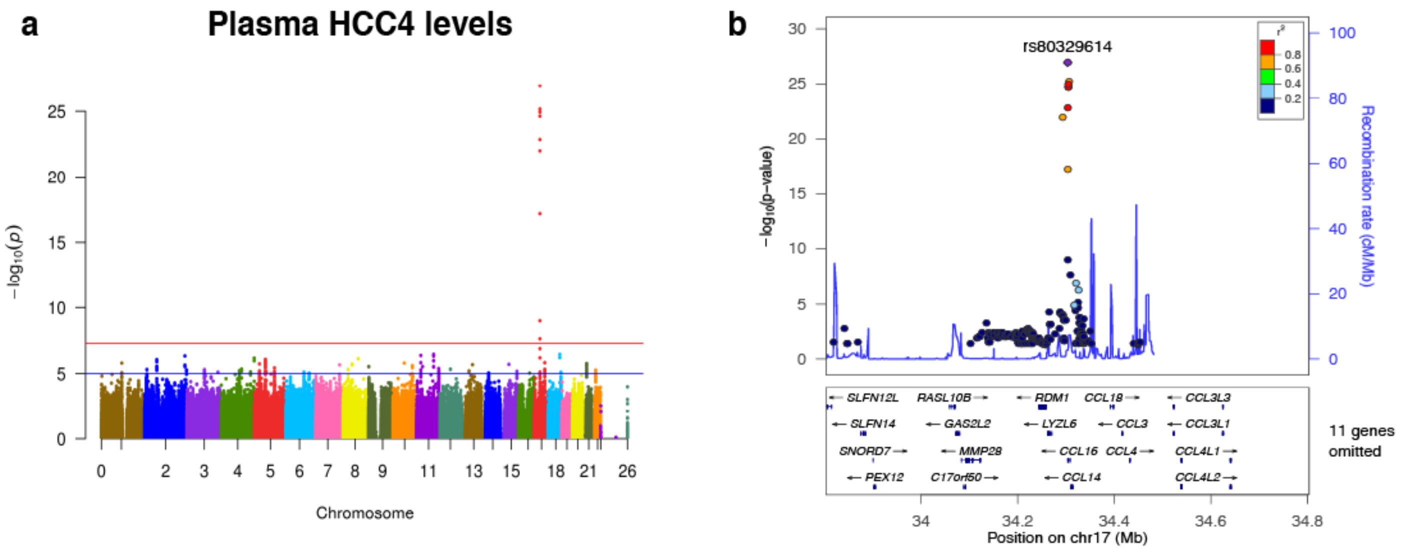


**Supplementary Figure S15. Manhattan and regional plots for associations with plasma levels of HCC4.** a) Manhattan plot of -$\log_{10}$ p-values for association with plasma levels of HCC4; b) Regional plot for genome-wide significant association with HCC4 plasma levels.

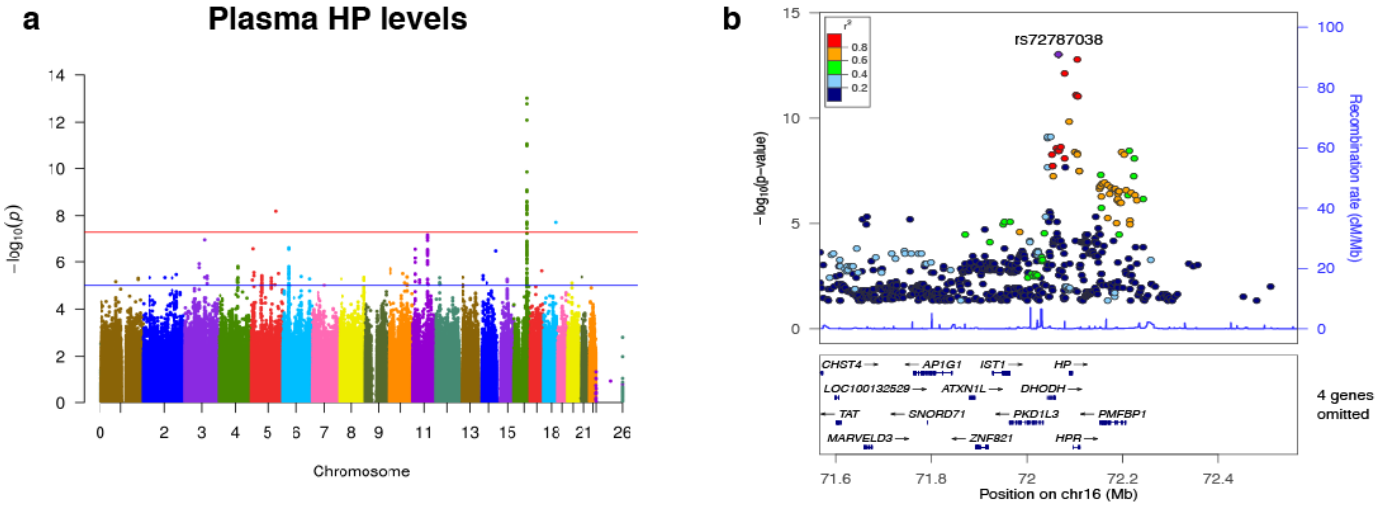


**Supplementary Figure S16. Manhattan and regional plots for associations with plasma levels of HP.** a) Manhattan plot of -$\log_{10}$ p-values for association with plasma levels of HP; b) Regional plot for genome-wide significant association with HP plasma levels.

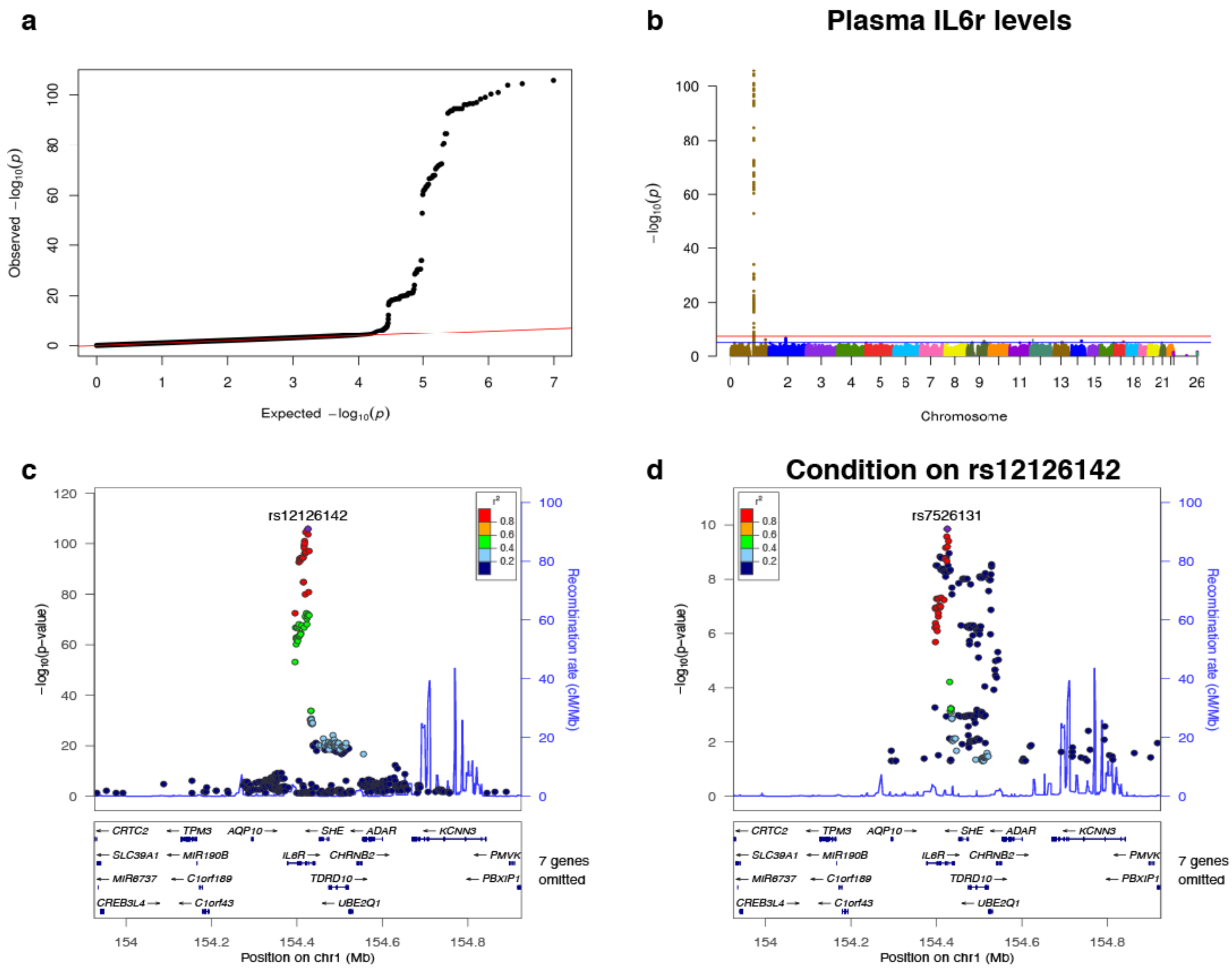


**Supplementary Figure S17. Q-Q, Manhattan and regional plots for associations with plasma levels of IL6r.** a) Q-Q plot of -log$_{10}$ p-values, expected versus observed, from joint GWAS of plasma IL6r levels; b) Manhattan plot of -log$_{10}$ p-values for association with plasma levels of IL6r; c) Regional plot for genome-wide significant association with IL6r plasma levels; d) Regional plot for genome-wide significant association with Il6r plasma levels after conditioning on rs12126142.

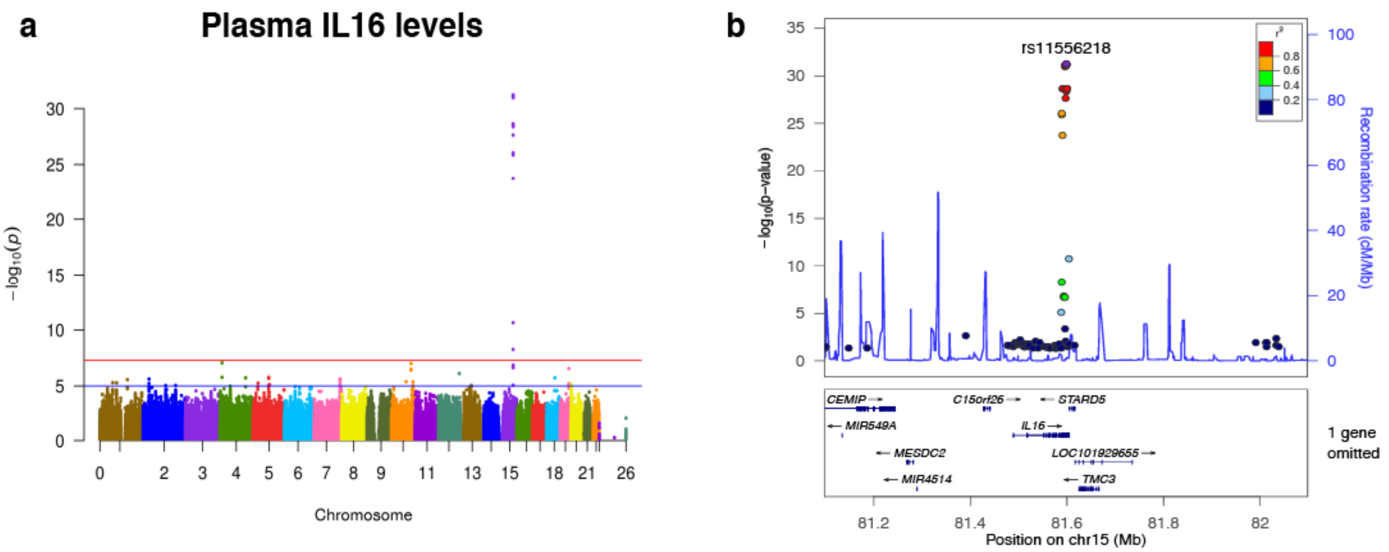


**Supplementary Figure S18. Manhattan and regional plots for associations with plasma levels of IL16.** a) Manhattan plot of -$\log_{10}$ p-values for association with plasma levels of IL16; b) Regional plot for genome-wide significant association with IL16 plasma levels.

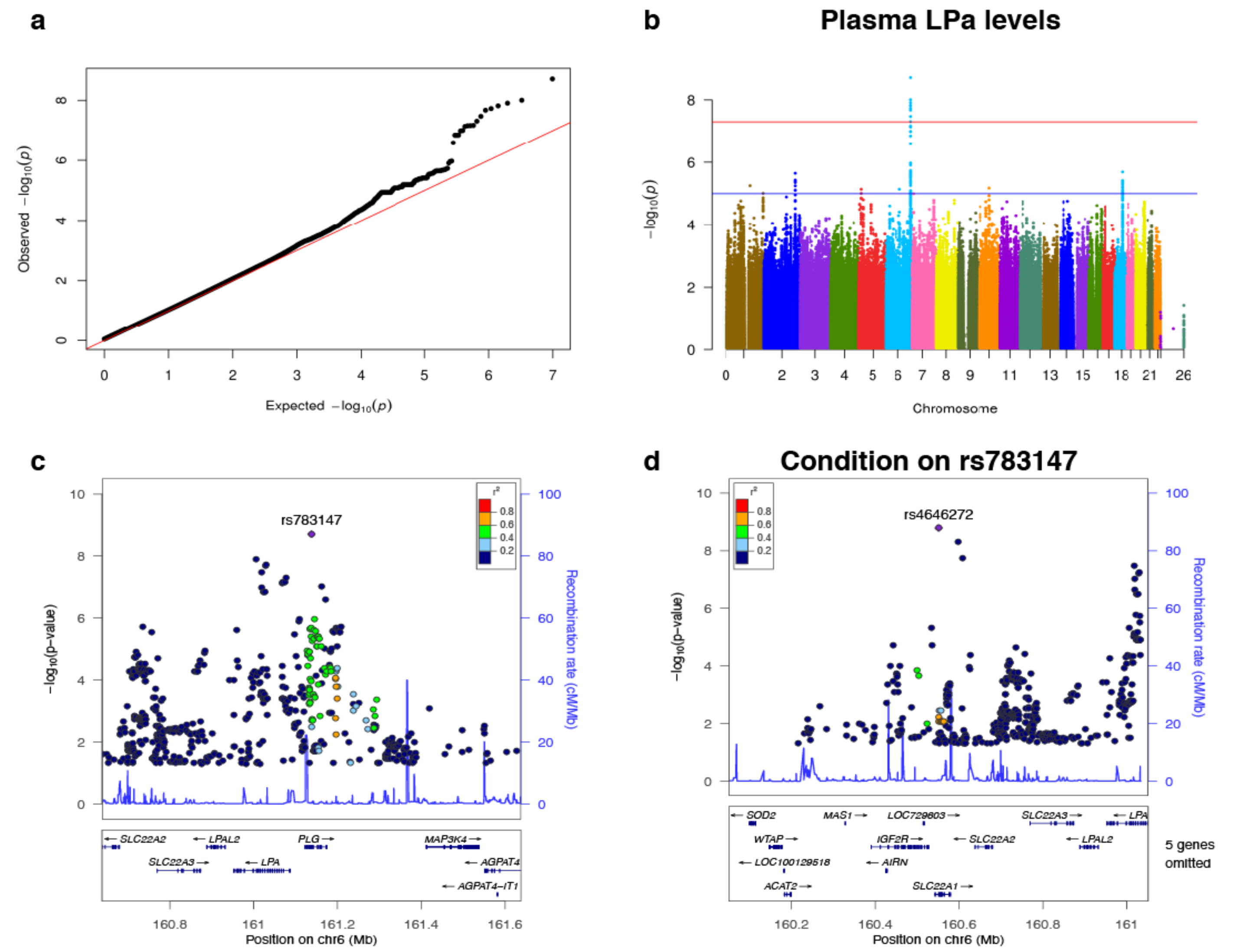


**Supplementary Figure S19. Q-Q, Manhattan and regional plots for associations with plasma levels of LPa.** a) Q-Q plot of -$\log_{10}$ p-values, expected versus observed, from joint GWAS of plasma LPa levels; b) Manhattan plot of -$\log_{10}$ p-values for association with plasma levels of LPa; c) Regional plot for genome-wide significant association with LPa plasma levels; d) Regional plot for genome-wide significant association with LPa plasma levels after conditioning on rs783147.

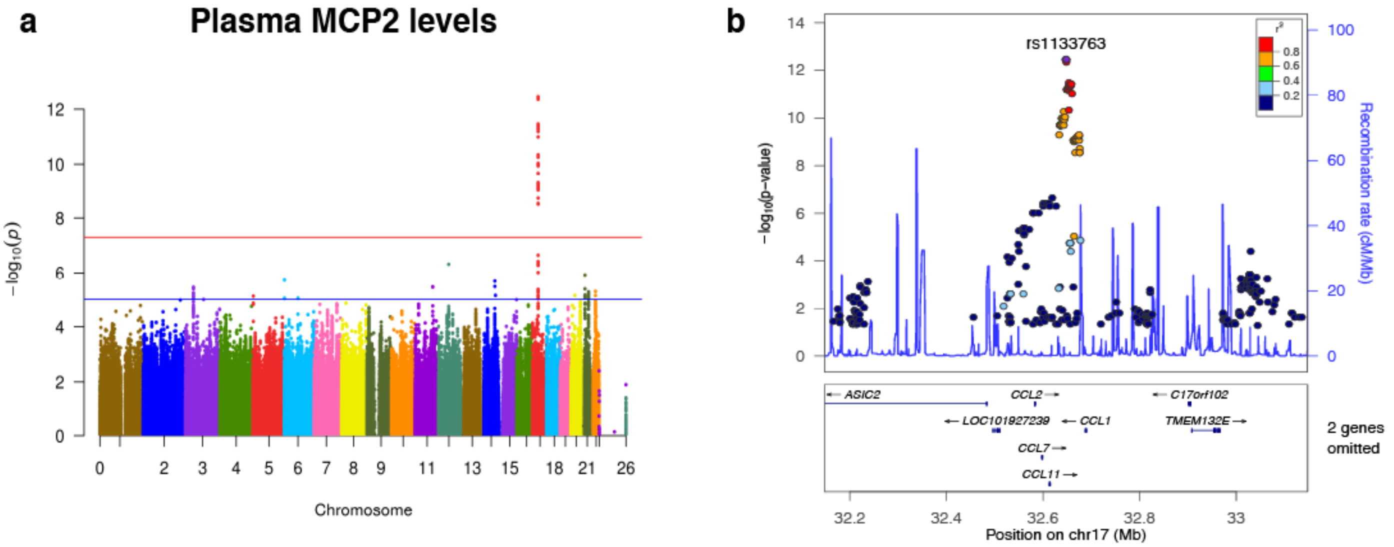


**Supplementary Figure S20. Manhattan and regional plots for associations with plasma levels of MCP2.** a) Manhattan plot of -$\log_{10}$ p-values for association with plasma levels of MCP2; b) Regional plot for genome-wide significant association with MCP2 plasma levels.

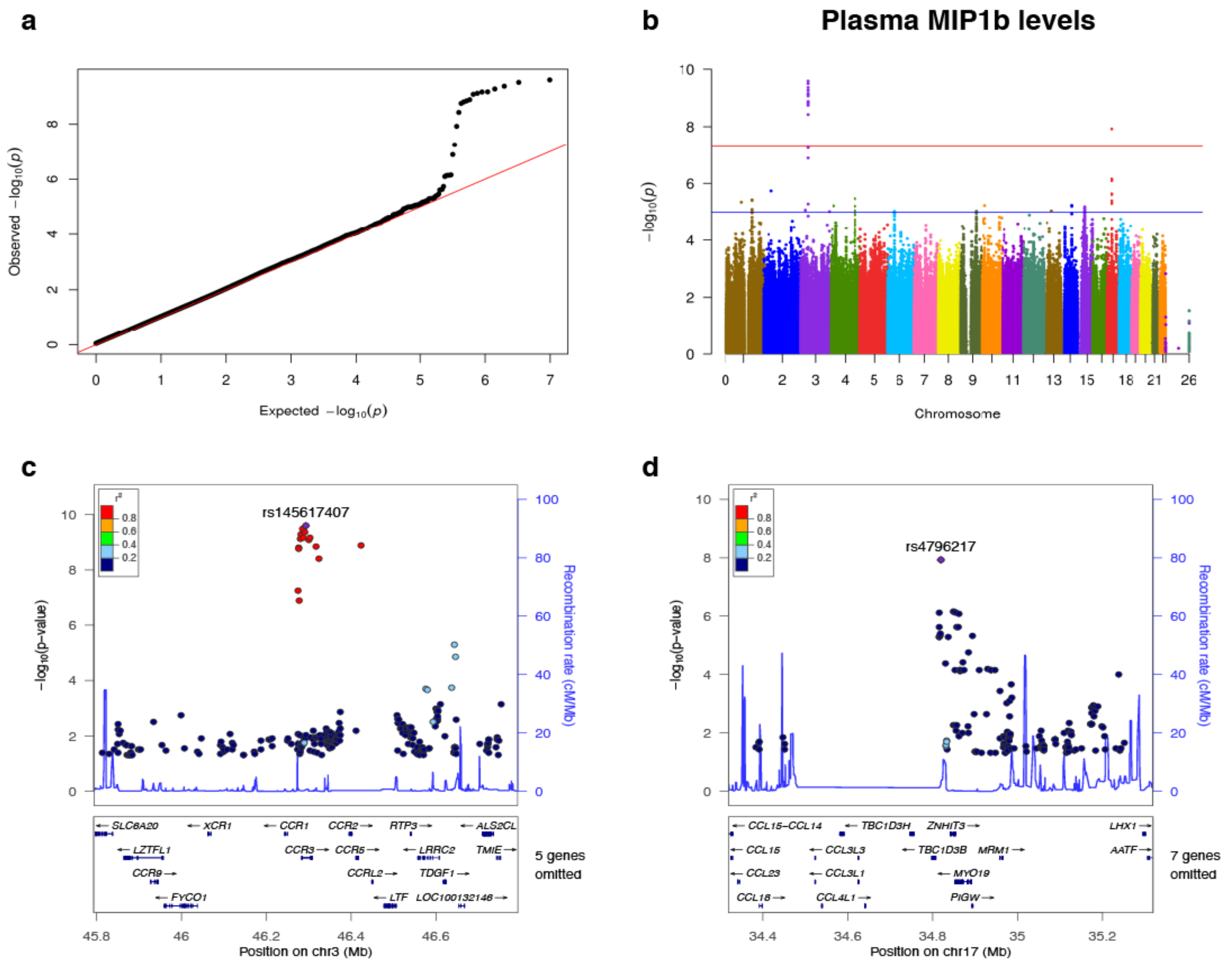


**Supplementary Figure S21. Q-Q, Manhattan and regional plots for associations with plasma levels of MIP1b.** a) Q-Q plot of -log$_{10}$ p-values, expected versus observed, from joint GWAS of plasma MIP1b levels; b) Manhattan plot of -log$_{10}$ p-values for association with plasma levels of MIP1b; c) Regional plot for genome-wide significant association on chromosome 3 with MIP1b plasma levels; d) Regional plot for genome-wide significant association on chromosome 17 with MIP1b plasma levels.

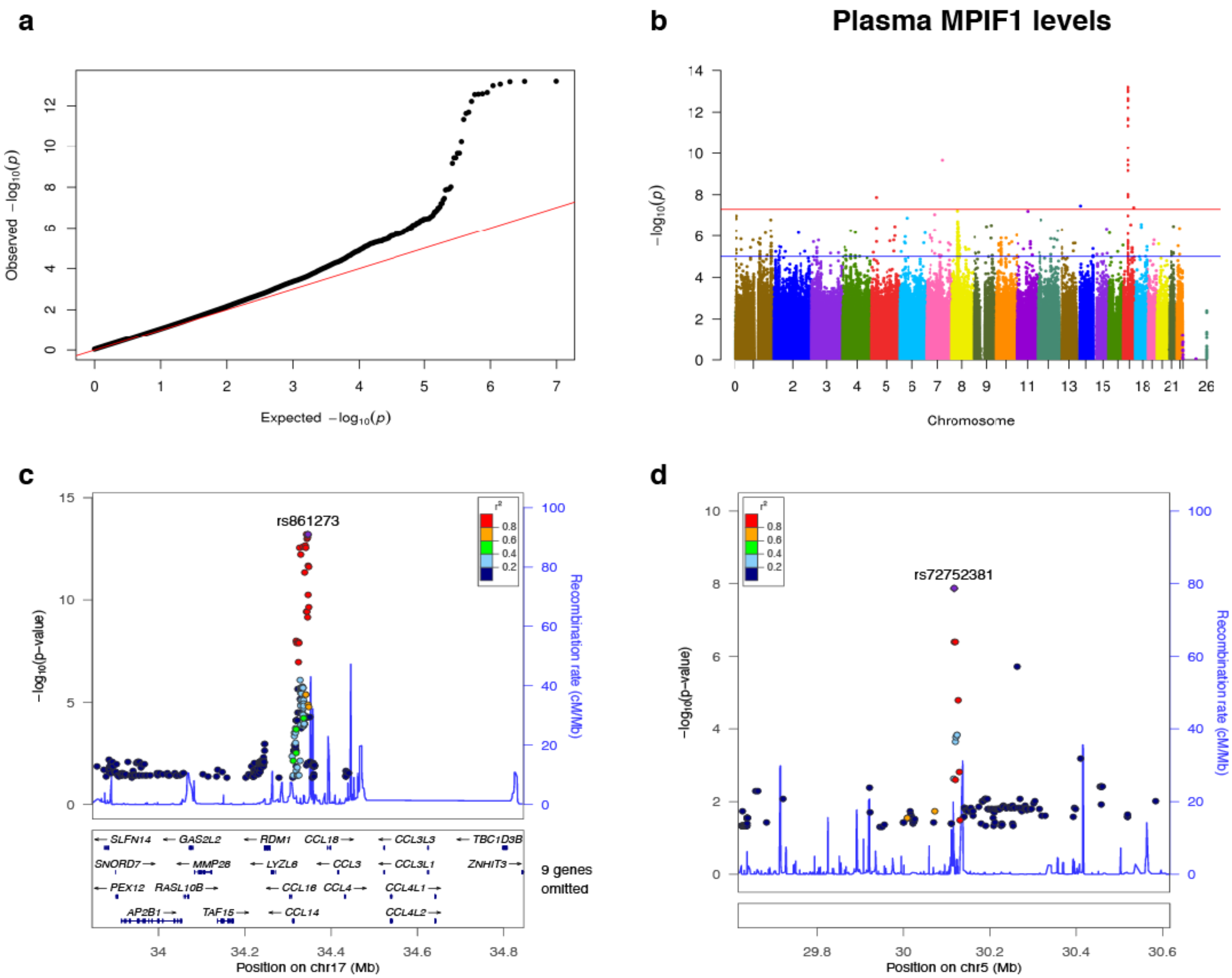


**Supplementary Figure S22. Q-Q, Manhattan and regional plots for associations with plasma levels of MPIF1.** a) Q-Q plot of $-\log_{10}$ p-values, expected versus observed, from joint GWAS of plasma MPIF1 levels; b) Manhattan plot of $-\log_{10}$ p-values for association with plasma levels of MPIF1; c) Regional plot for genome-wide significant association on chromosome 17 with MPIF1 plasma levels; d) Regional plot for genome-wide significant association on chromosome 5 with MPIF1 plasma levels.

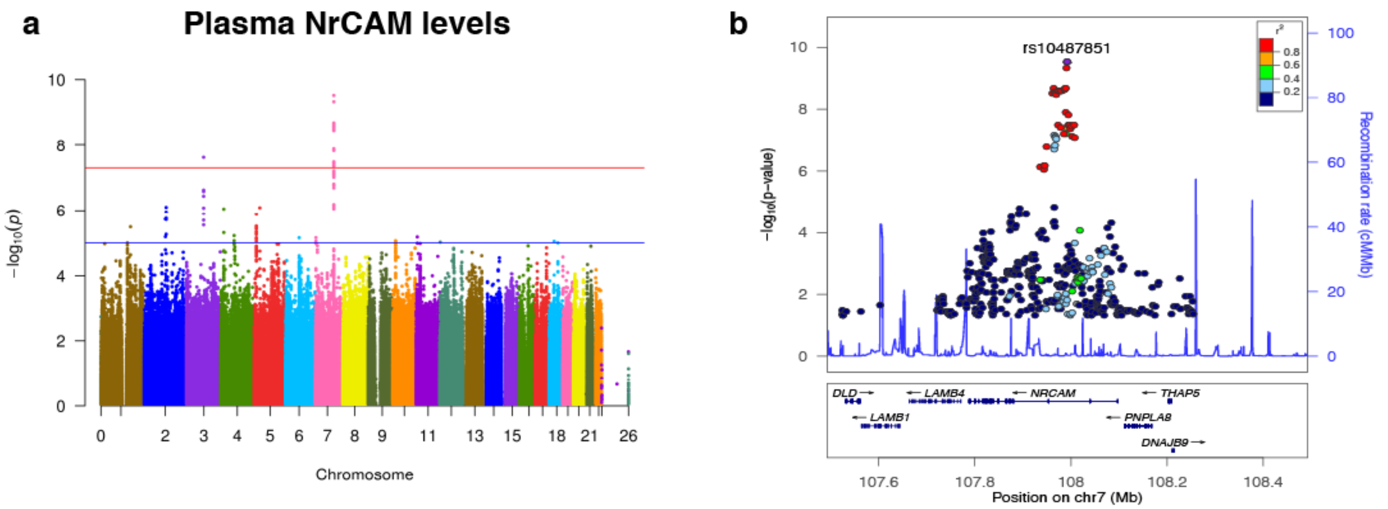


**Supplementary Figure S23. Manhattan and regional plots for associations with plasma levels of NrCAM.** a) Manhattan plot of -$\log_{10}$ p-values for association with plasma levels of NrCAM; b) Regional plot for genome-wide significant association with NrCAM plasma levels.

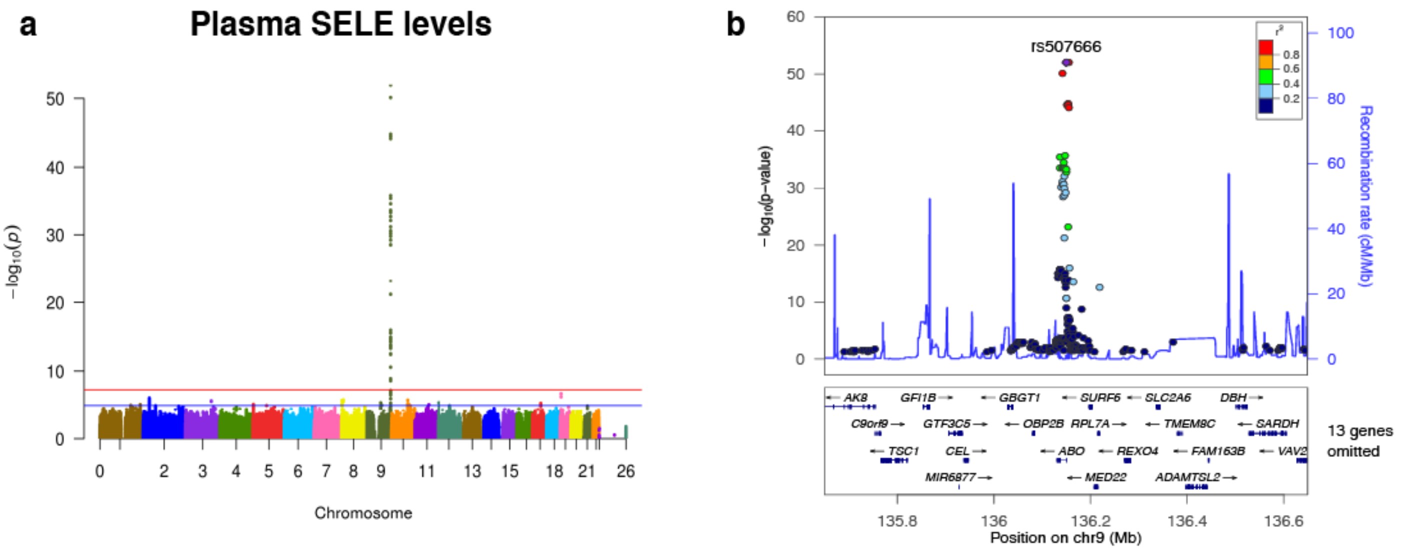


**Supplementary Figure S24. Manhattan and regional plots for associations with plasma levels of SELE.** a) Manhattan plot of -$\log_{10}$ p-values for association with plasma levels of SELE; b) Regional plot for genome-wide significant association with SELE plasma levels.

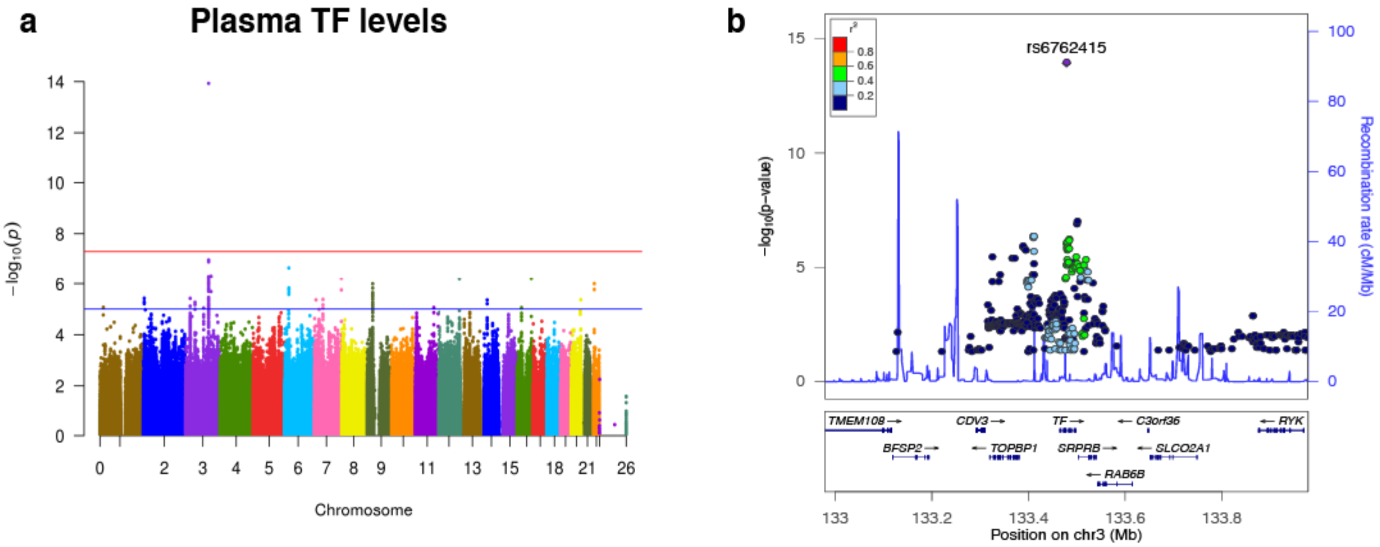


**Supplementary Figure S25. Manhattan and regional plots for associations with plasma levels of TF.** a) Manhattan plot of -$\log_{10}$ p-values for association with plasma levels of TF; b) Regional plot for genome-wide significant association with TF plasma levels.

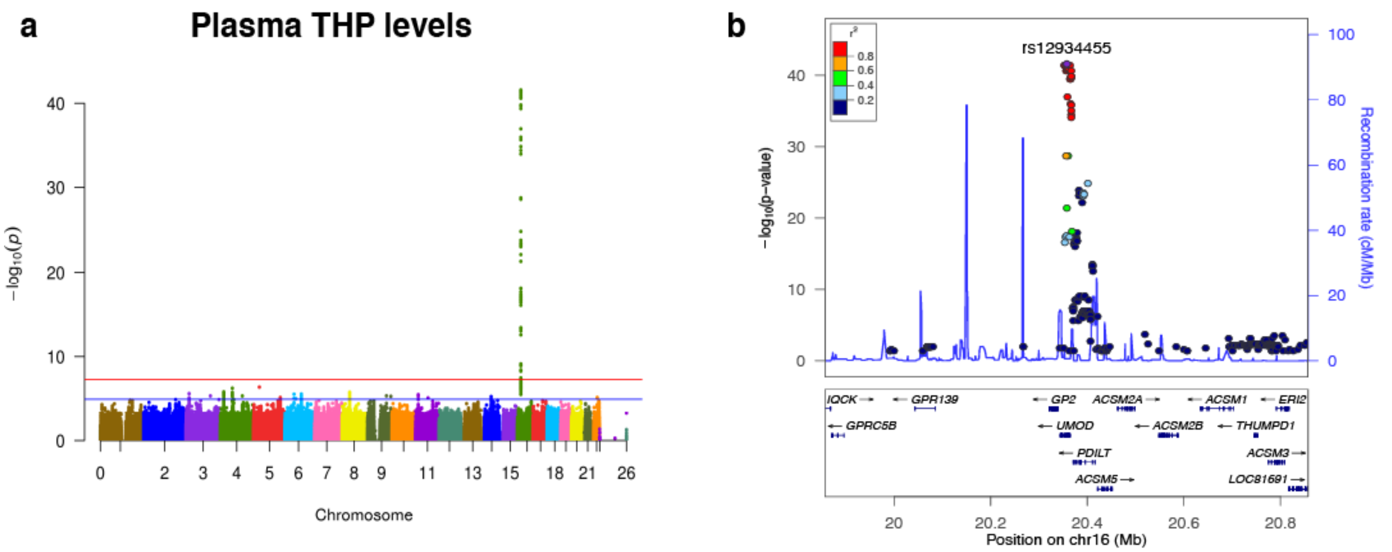


**Supplementary Figure S26. Manhattan and regional plots for associations with plasma levels of THP.** a) Manhattan plot of -$\log_{10}$ p-values for association with plasma levels of THP; b) Regional plot for genome-wide significant association with THP plasma levels.

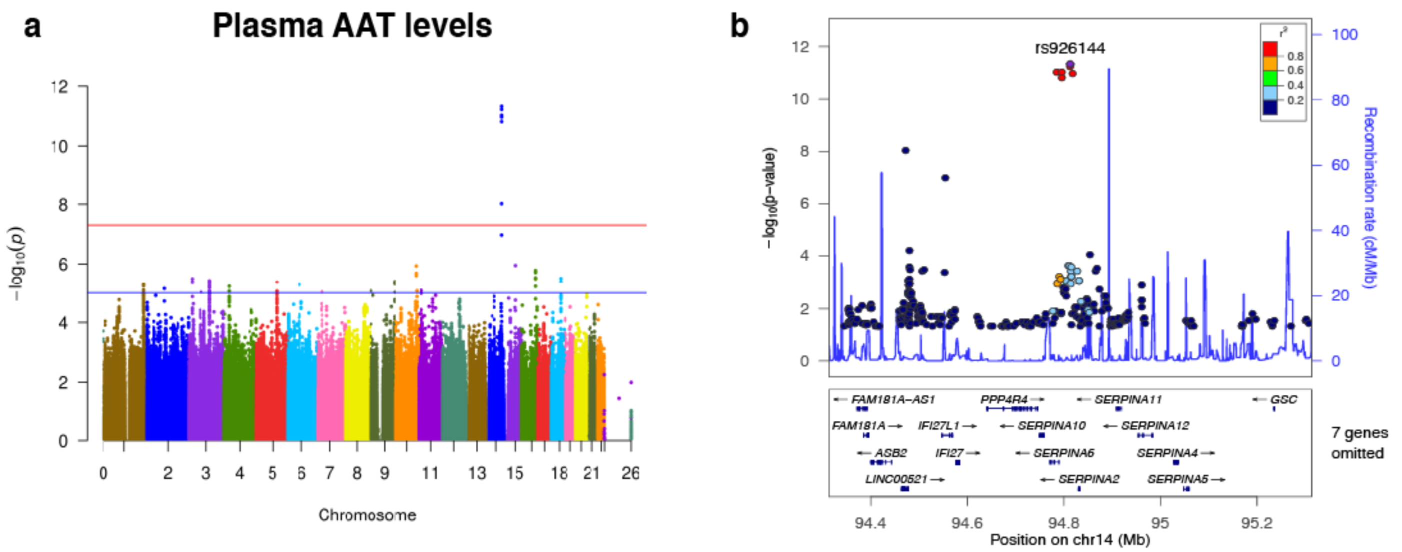


**Supplementary Figure S27. Manhattan and regional plots for associations with plasma levels of AAT.** a) Manhattan plot of -$\log_{10}$ p-values for association with plasma levels of AAT; b) Regional plot for genome-wide significant association with AAT plasma levels.

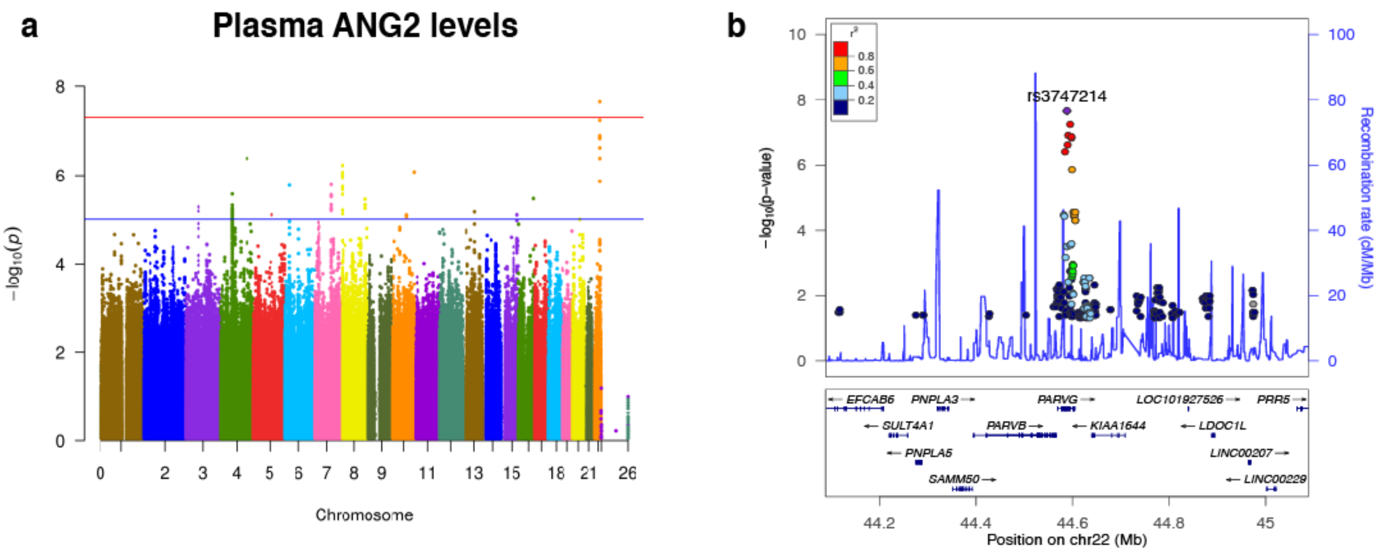


**Supplementary Figure S28. Manhattan and regional plots for associations with plasma levels of ANG2.** a) Manhattan plot of -$\log_{10}$ p-values for association with plasma levels of ANG2; b) Regional plot for genome-wide significant association with ANG2 plasma levels.

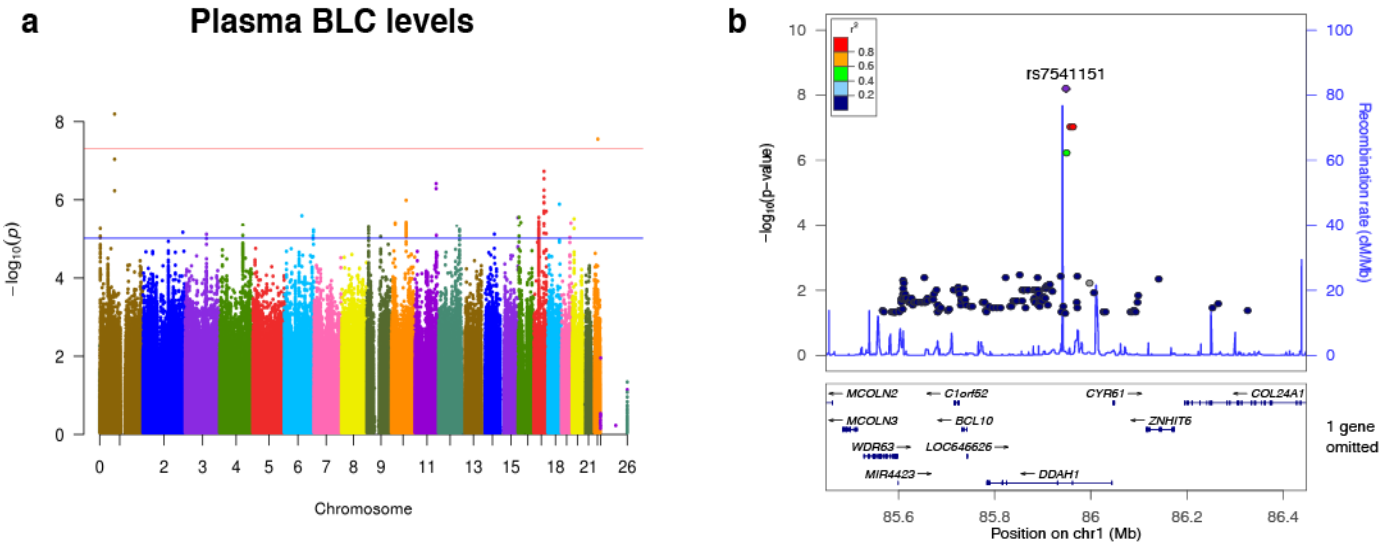


**Supplementary Figure S29. QQ, Manhattan, and regional plots for associations with plasma levels of BLC.** a) Manhattan plot of -$\log_{10}$ p-values for association with plasma levels of BLC; b) Regional plot for genome-wide significant association with BLC plasma levels.

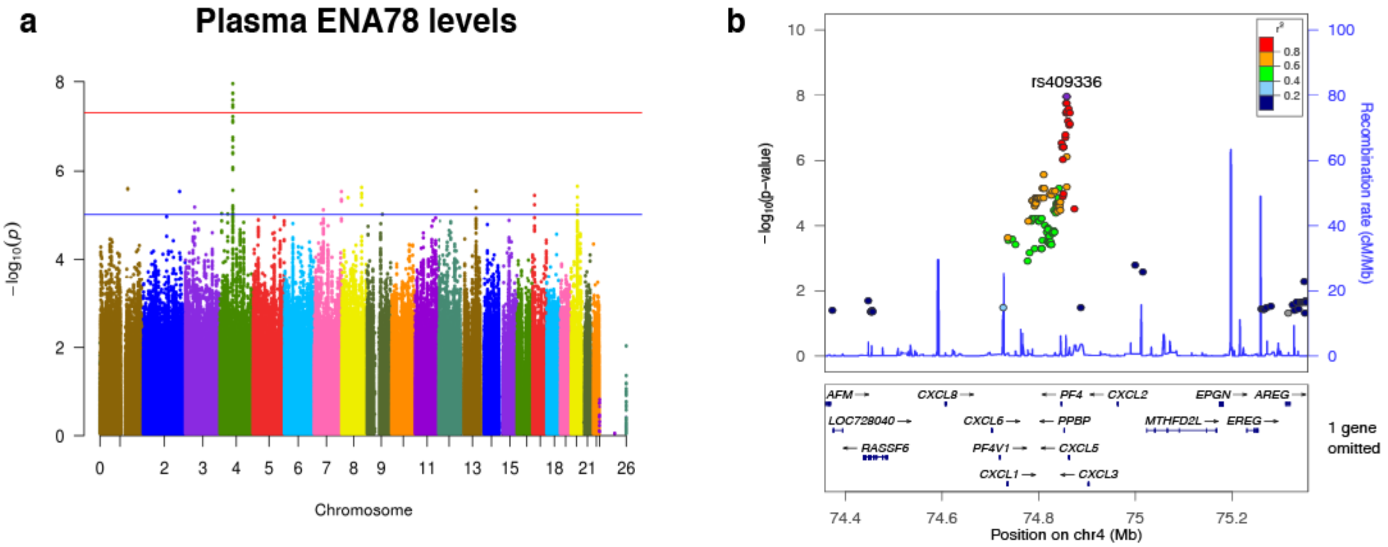


**Supplementary Figure S30. Manhattan and regional plots for associations with plasma levels of ENA78.** a) Manhattan plot of -$\log_{10}$ p-values for association with plasma levels of ENA78; b) Regional plot for genome-wide significant association with ENA78 plasma levels.

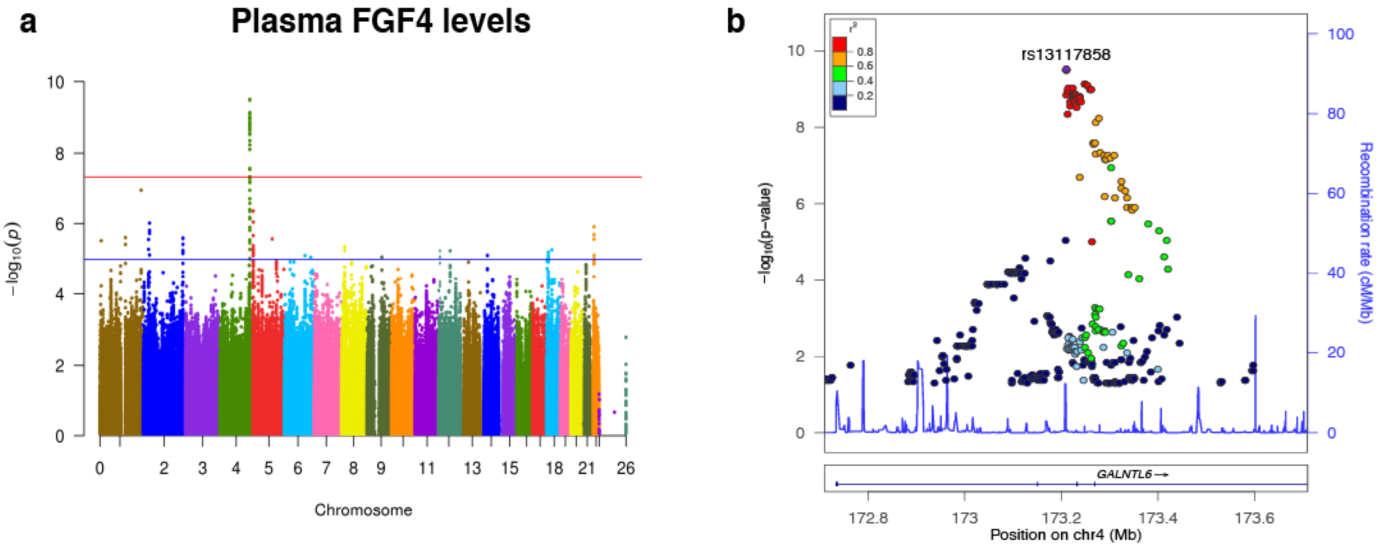


**Supplementary Figure S31. Manhattan and regional plots for associations with plasma levels of FGF4.** a) Manhattan plot of -$\log_{10}$ p-values for association with plasma levels of FGF4; b) Regional plot for genome-wide significant association with FGF4 plasma levels.

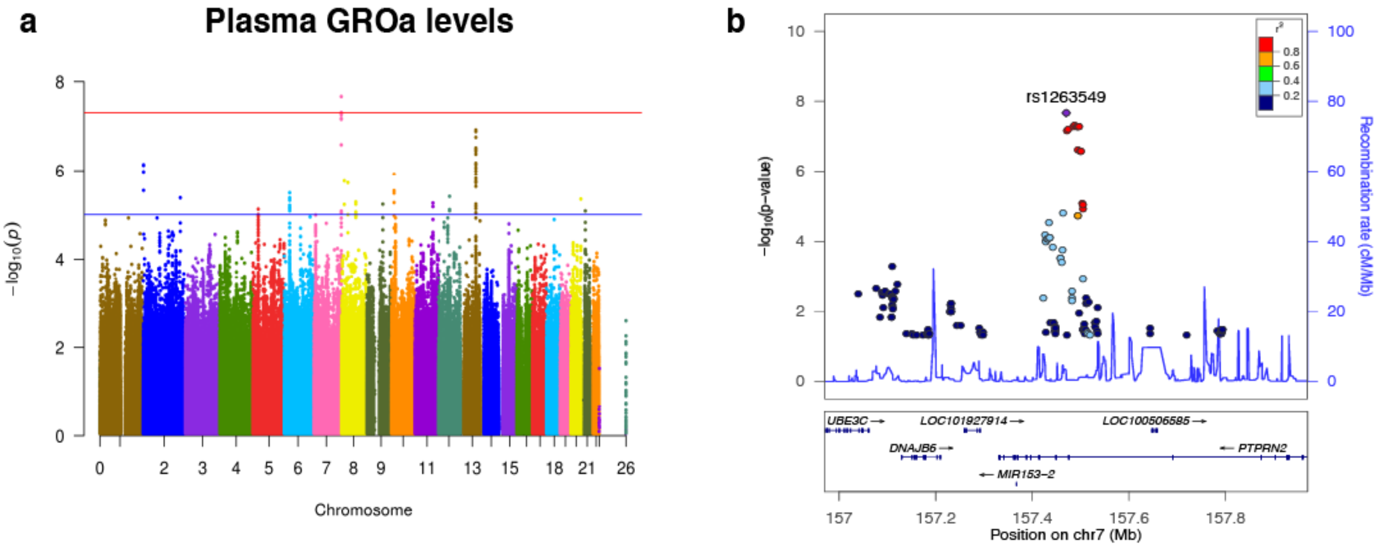


**Supplementary Figure S32. Manhattan and regional plots for associations with plasma levels of GROa.** a) Manhattan plot of -$\log_{10}$ p-values for association with plasma levels of GROa; b) Regional plot for genome-wide significant association with GROa plasma levels.

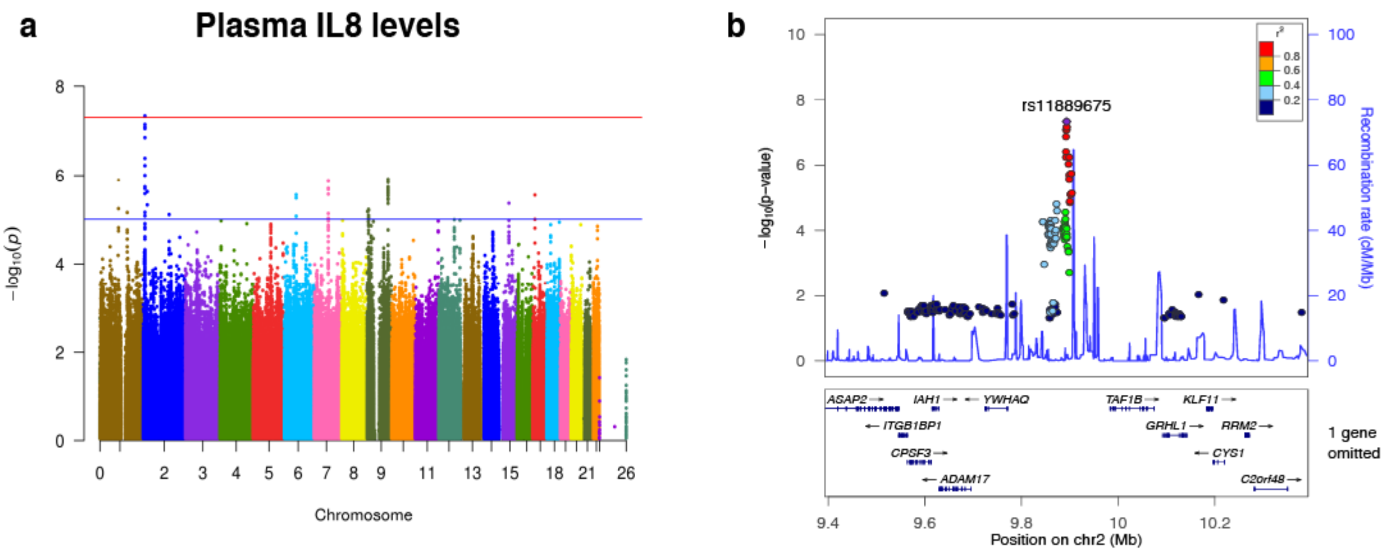


**Supplementary Figure S33. Manhattan and regional plots for associations with plasma levels of IL8.** a) Manhattan plot of -$\log_{10}$ p-values for association with plasma levels of IL8; b) Regional plot for genome-wide significant association with IL8 plasma levels.

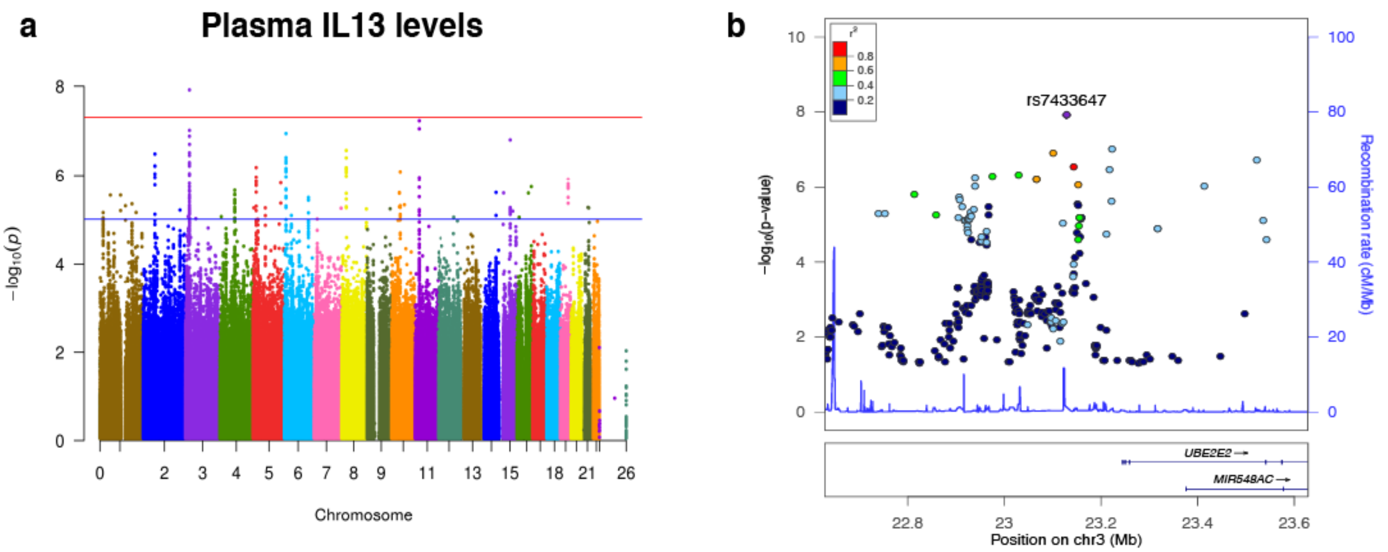


**Supplementary Figure S34. Manhattan and regional plots for associations with plasma levels of IL13.** a) Manhattan plot of -$\log_{10}$ p-values for association with plasma levels of IL13; b) Regional plot for genome-wide significant association with IL13 plasma levels.

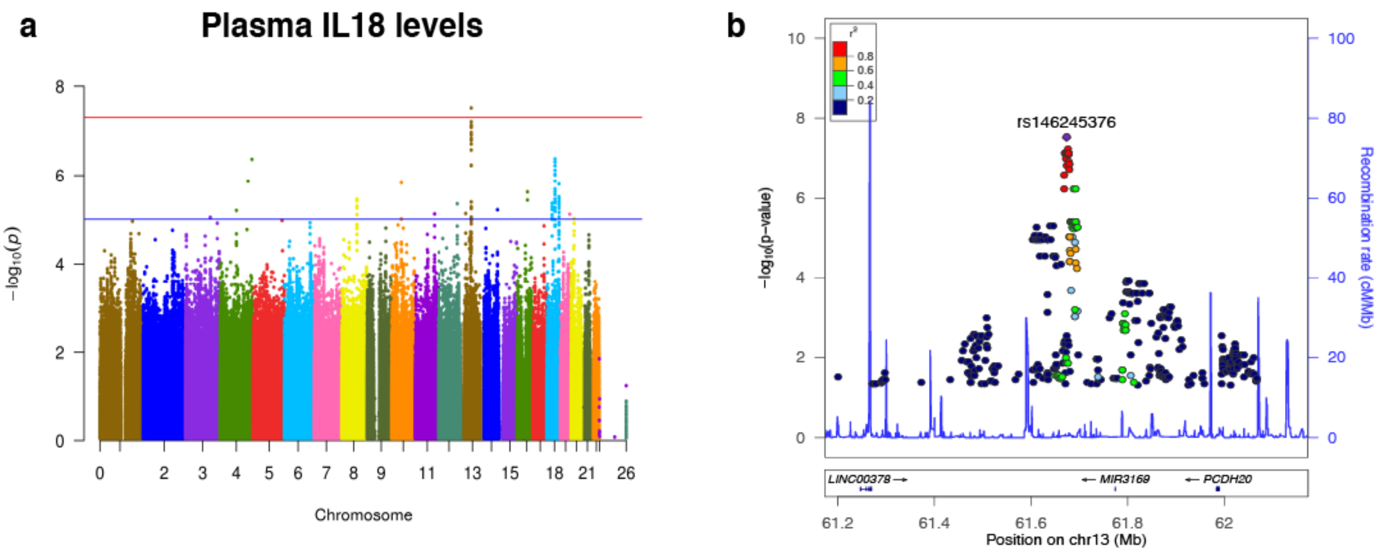


**Supplementary Figure S35. Manhattan and regional plots for associations with plasma levels of IL18.** a) Manhattan plot of -$\log_{10}$ p-values for association with plasma levels of IL18; b) Regional plot for genome-wide significant association with IL18 plasma levels.

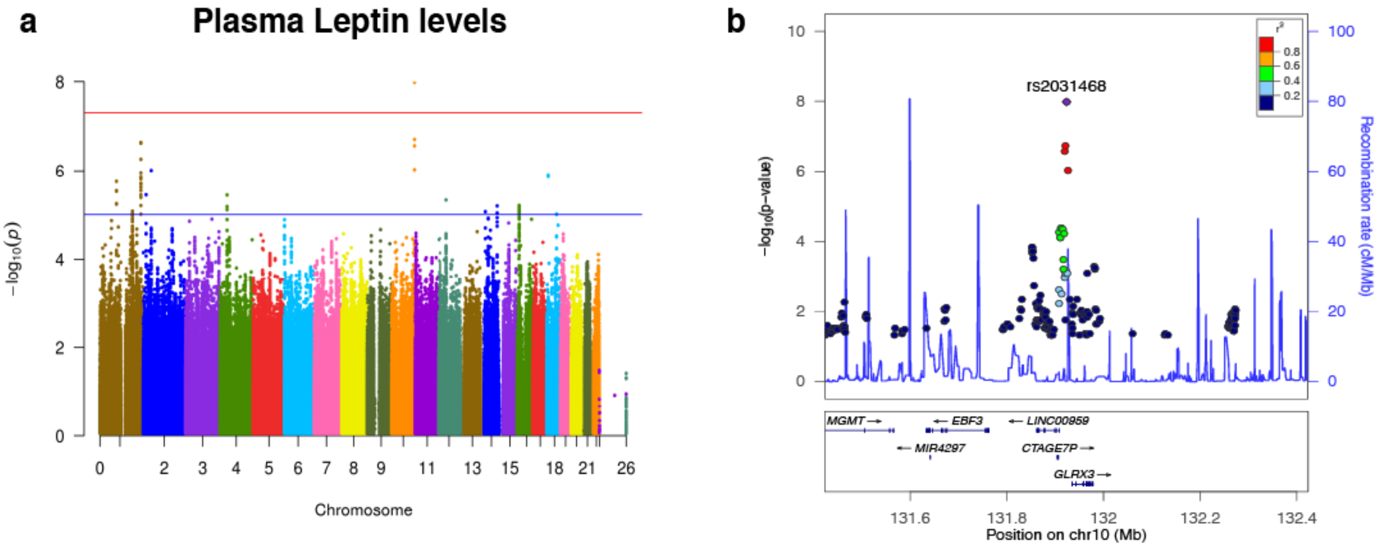


**Supplementary Figure S36. Manhattan and regional plots for associations with plasma levels of Leptin.** a) Manhattan plot of $-\log_{10}$ p-values for association with plasma levels of Leptin; b) Regional plot for genome-wide significant association with Leptin plasma levels.

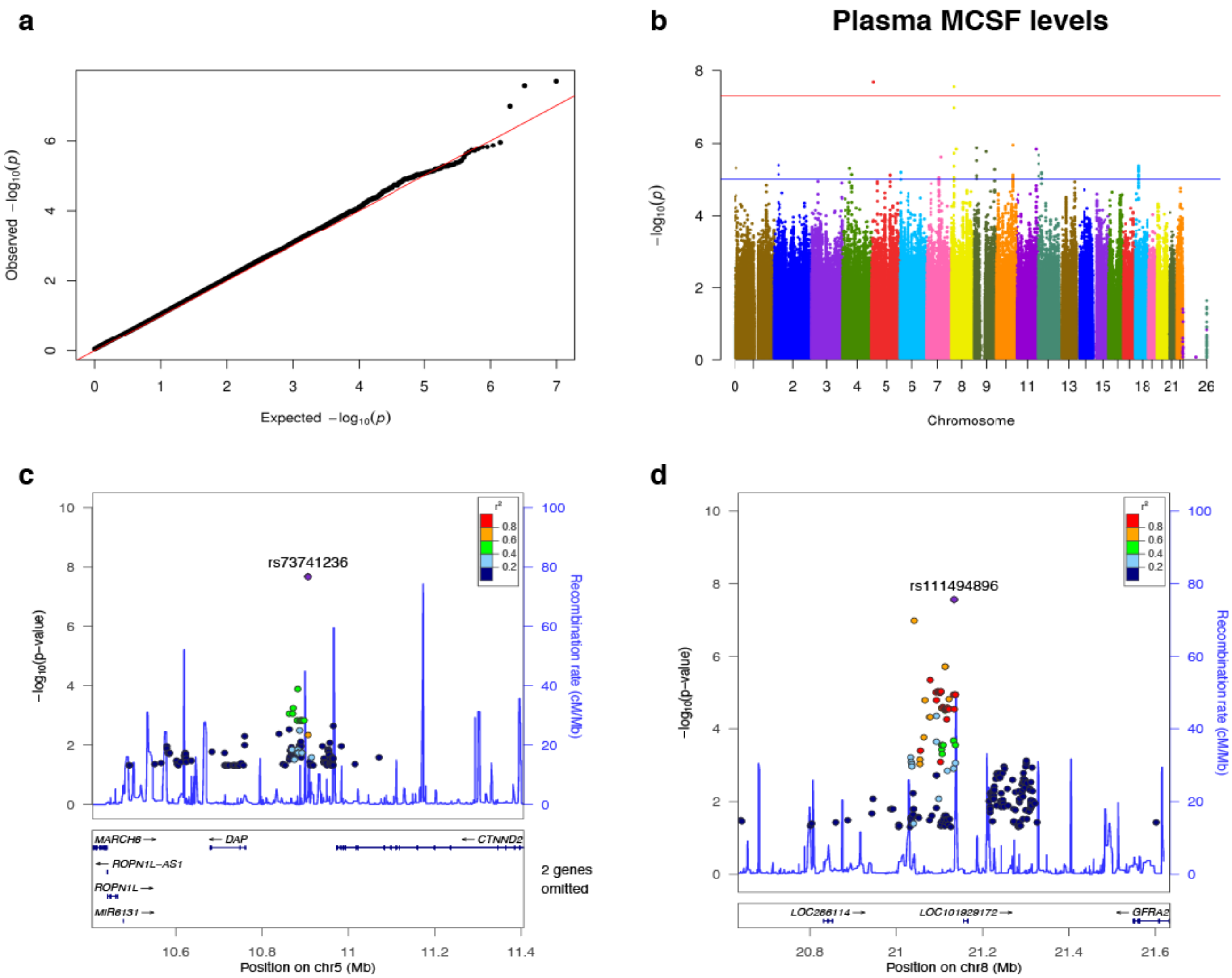


**Supplementary Figure S37. Q-Q, Manhattan and regional plots for associations with plasma levels of MCSF.** a) Q-Q plot of $-\log_{10}$ p-values, expected versus observed, from joint GWAS of plasma MCSF levels; b) Manhattan plot of $-\log_{10}$ p-values for association with plasma levels of MCSF; c) Regional plot for genome-wide significant association on chromosome 5 with MCSF plasma levels; d) Regional plot for genome-wide significant association on chromosome 8 with MCSF plasma levels.

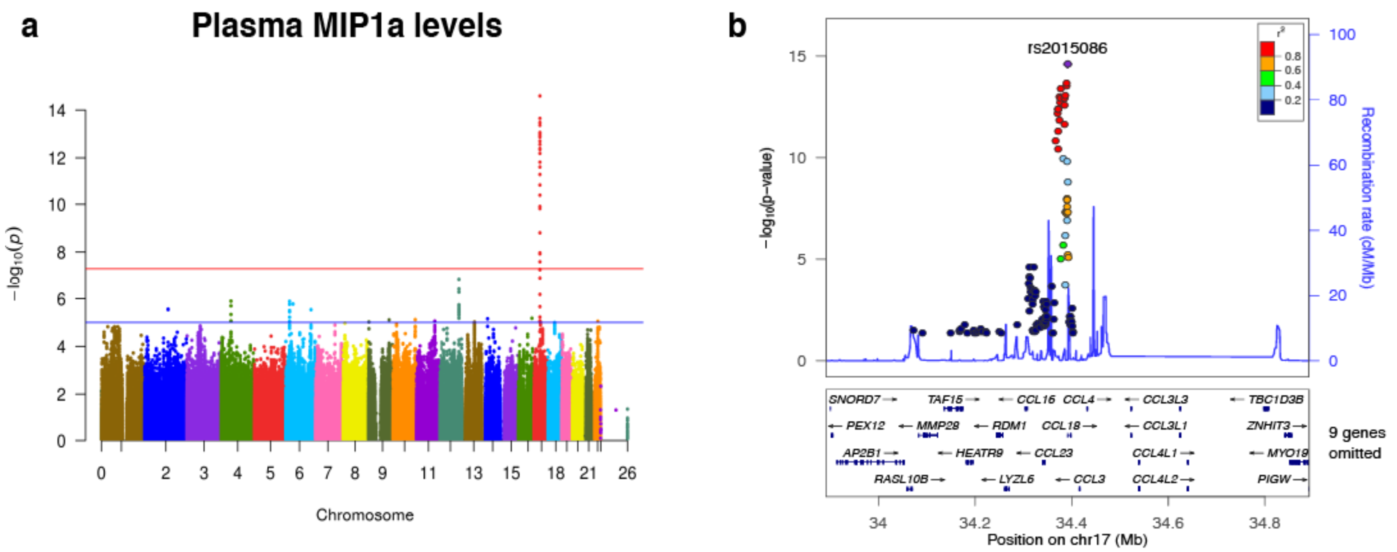


**Supplementary Figure S38. Manhattan and regional plots for associations with plasma levels of MIP1a.** a) Manhattan plot of $-\log_{10}$ p-values for association with plasma levels of MIP1a; b) Regional plot for genome-wide significant association with MIP1a plasma levels.

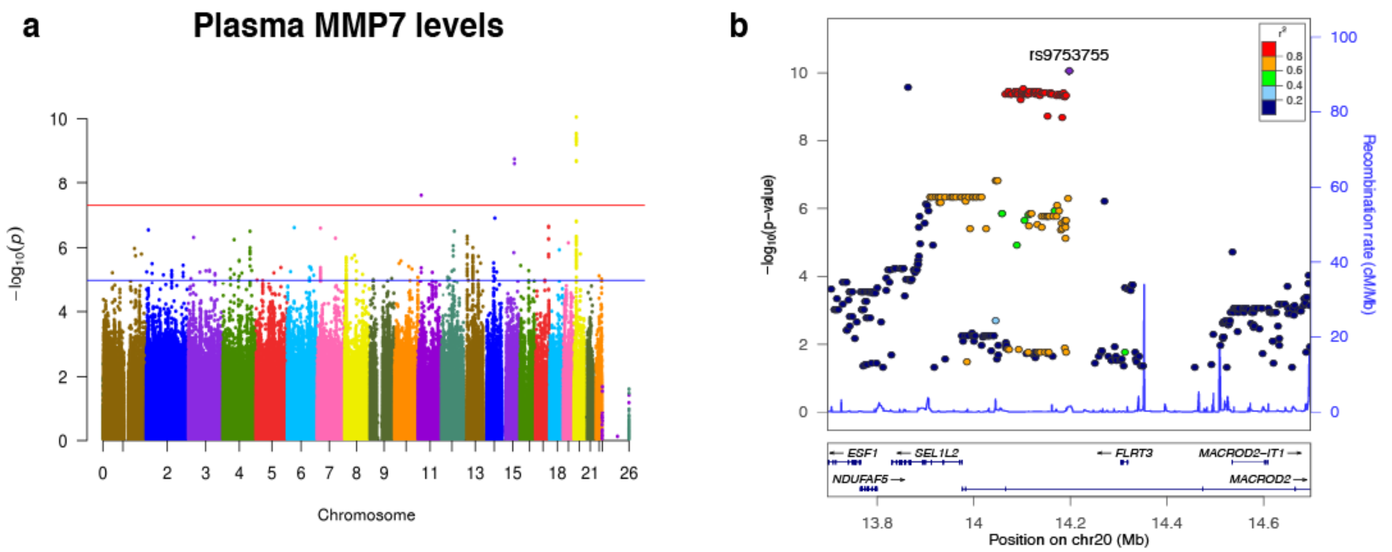


**Supplementary Figure S39. Manhattan and regional plots for associations with plasma levels of MMP7.** a) Manhattan plot of -$\log_{10}$ p-values for association with plasma levels of MMP7; b) Regional plot for genome-wide significant association with MMP7 plasma levels.

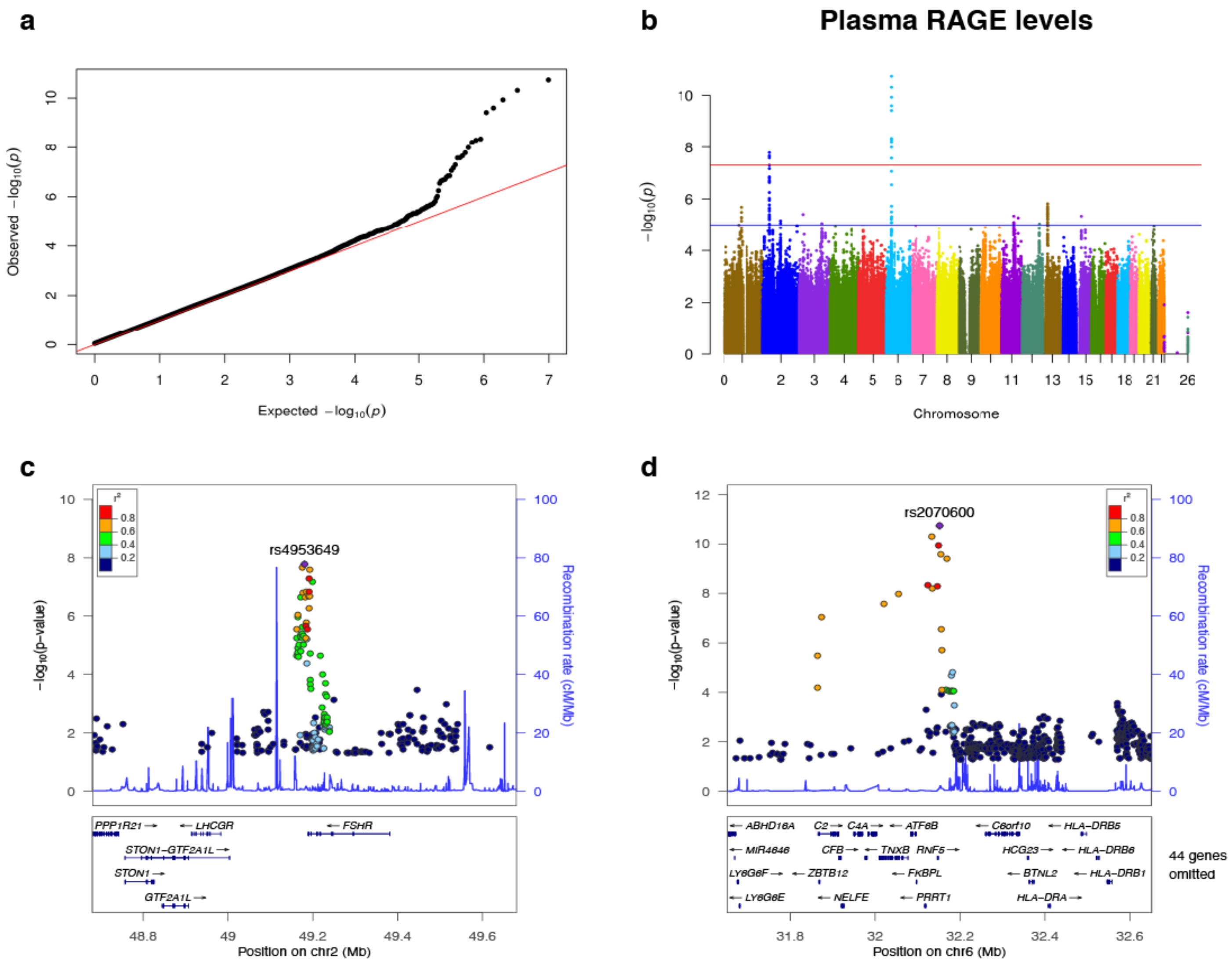


**Supplementary Figure S40. Q-Q, Manhattan and regional plots for associations with plasma levels of RAGE.** a) Q-Q plot of -log$_{10}$ p-values, expected versus observed, from joint GWAS of plasma RAGE levels; b) Manhattan plot of -log$_{10}$ p-values for association with plasma levels of RAGE; c) Regional plot for genome-wide significant association on chromosome 2 with RAGE plasma levels; d) Regional plot for genome-wide significant association on chromosome 6 with RAGE plasma levels.

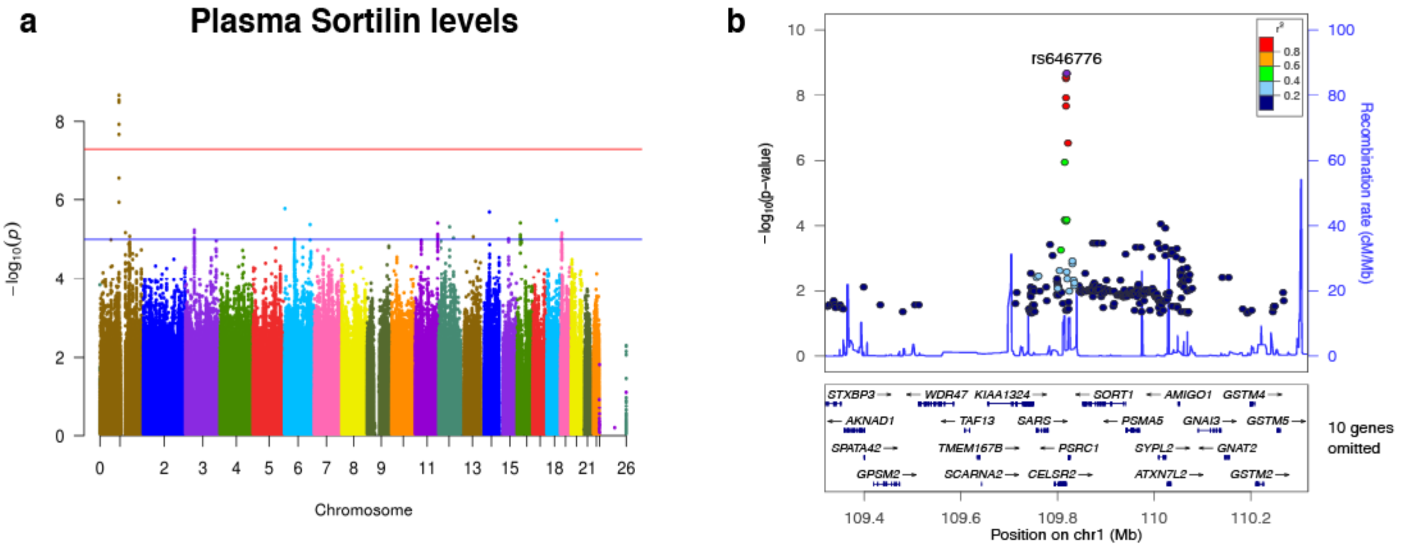


**Supplementary Figure S41. Manhattan and regional plots for associations with plasma levels of Sortilin.** a) Manhattan plot of -$\log_{10}$ p-values for association with plasma levels of Sortilin; b) Regional plot for genome-wide significant association with Sortilin plasma levels.

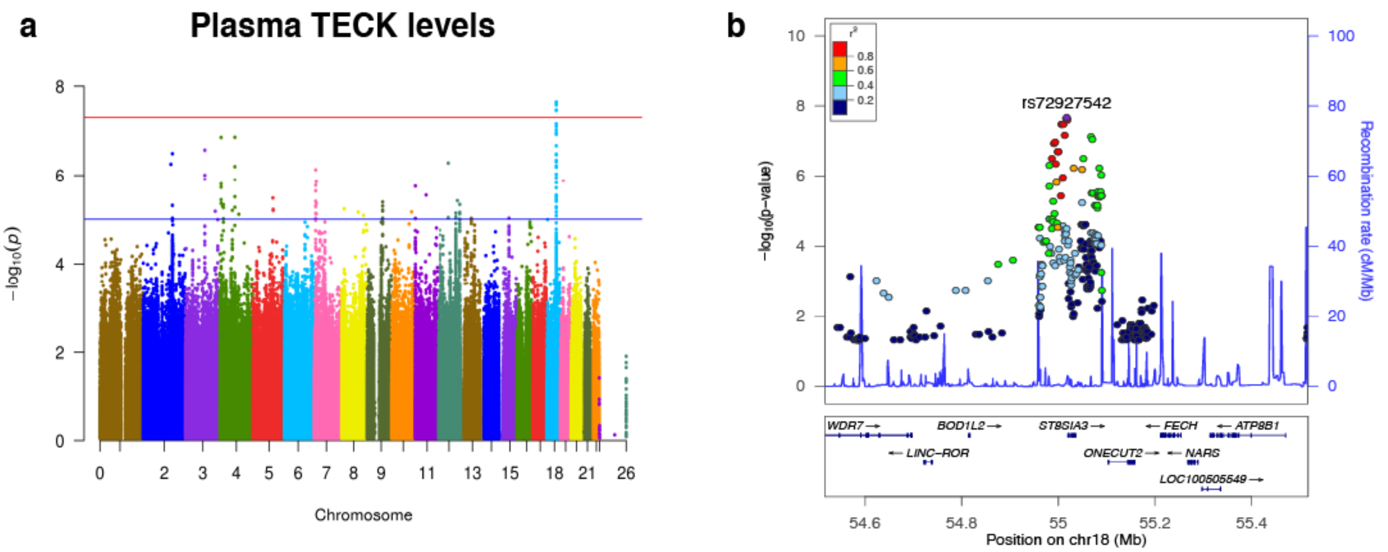


**Supplementary Figure S42. Manhattan and regional plots for associations with plasma levels of TECK.** a) Manhattan plot of -$\log_{10}$ p-values for association with plasma levels of TECK; b) Regional plot for genome-wide significant association with TECK plasma levels.

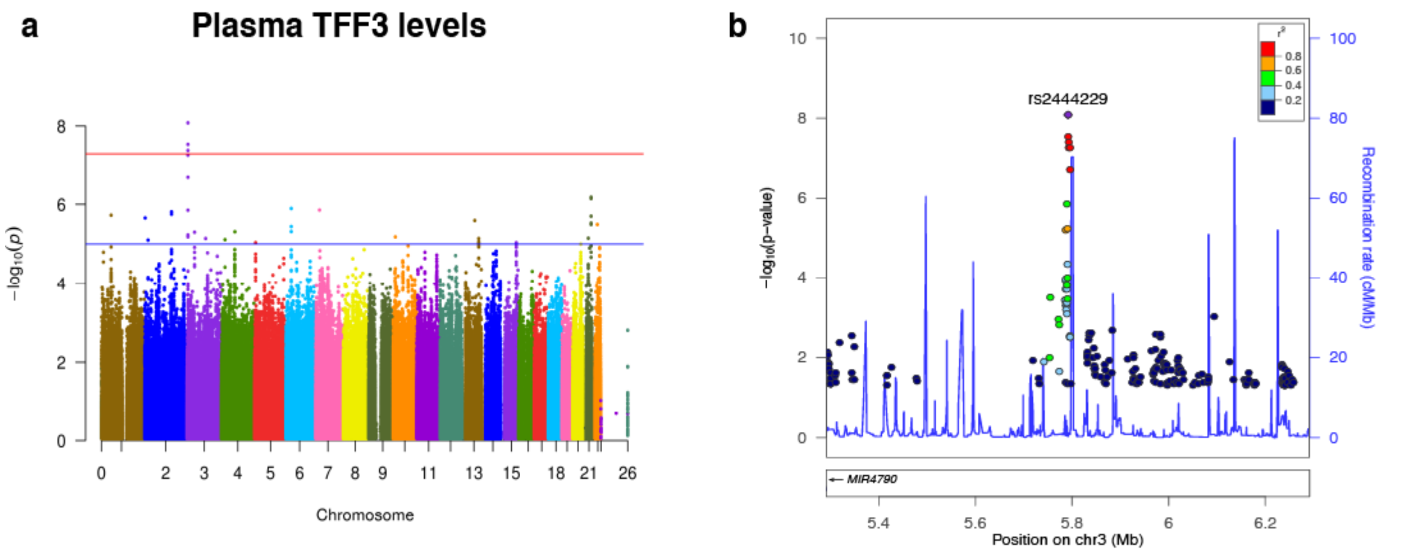


**Supplementary Figure S43. Manhattan and regional plots for associations with plasma levels of TFF3.** a) Manhattan plot of -$\log_{10}$ p-values for association with plasma levels of TFF3; b) Regional plot for genome-wide significant association with TFF3 plasma levels.

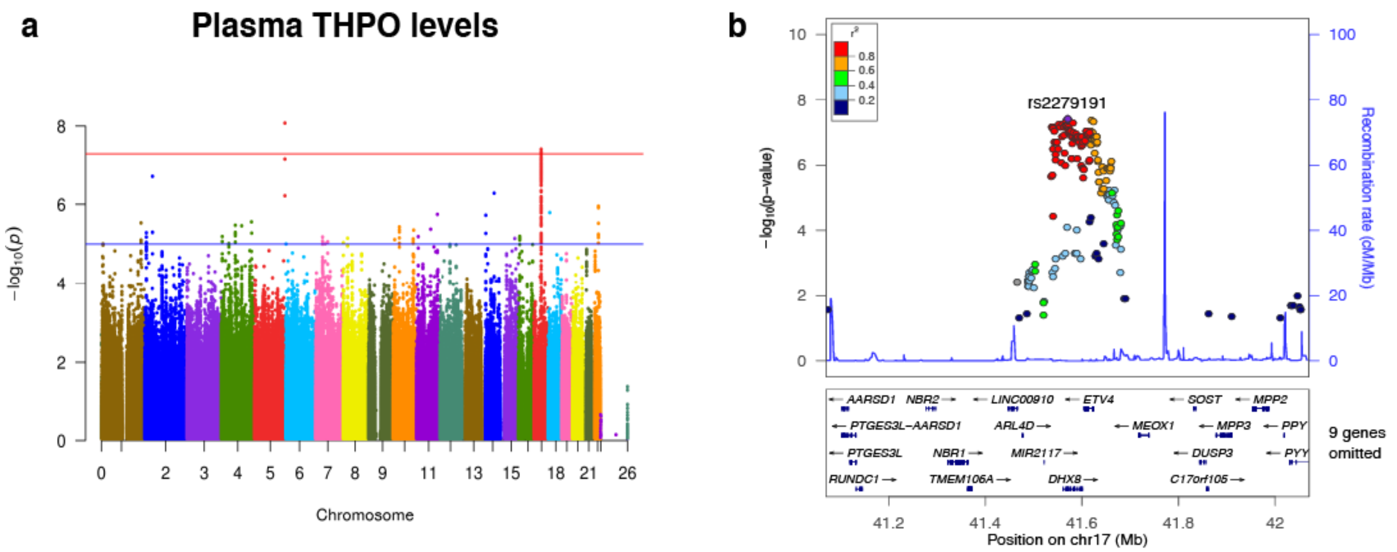


**Supplementary Figure S44. Manhattan and regional plots for associations with plasma levels of THPO.** a) Manhattan plot of -$\log_{10}$ p-values for association with plasma levels of THPO; b) Regional plot for genome-wide significant association with THPO plasma levels.

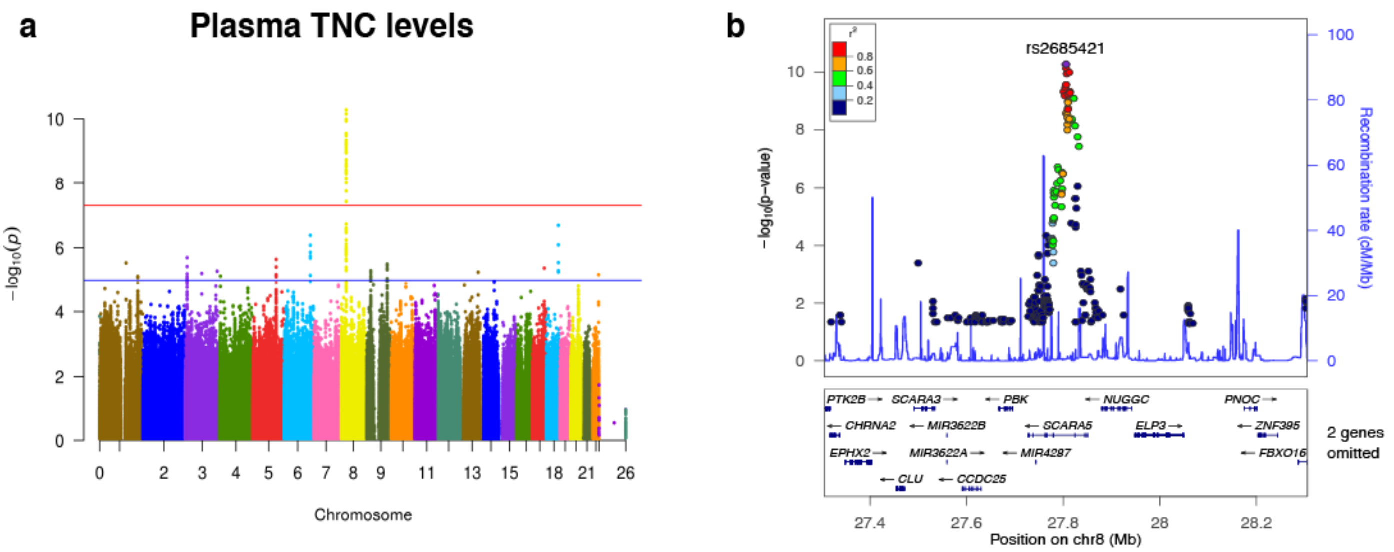


**Supplementary Figure S45. Manhattan and regional plots for associations with plasma levels of TNC.** a) Manhattan plot of -$\log_{10}$ p-values for association with plasma levels of TNC; b) Regional plot for genome-wide significant association with TNC plasma levels.

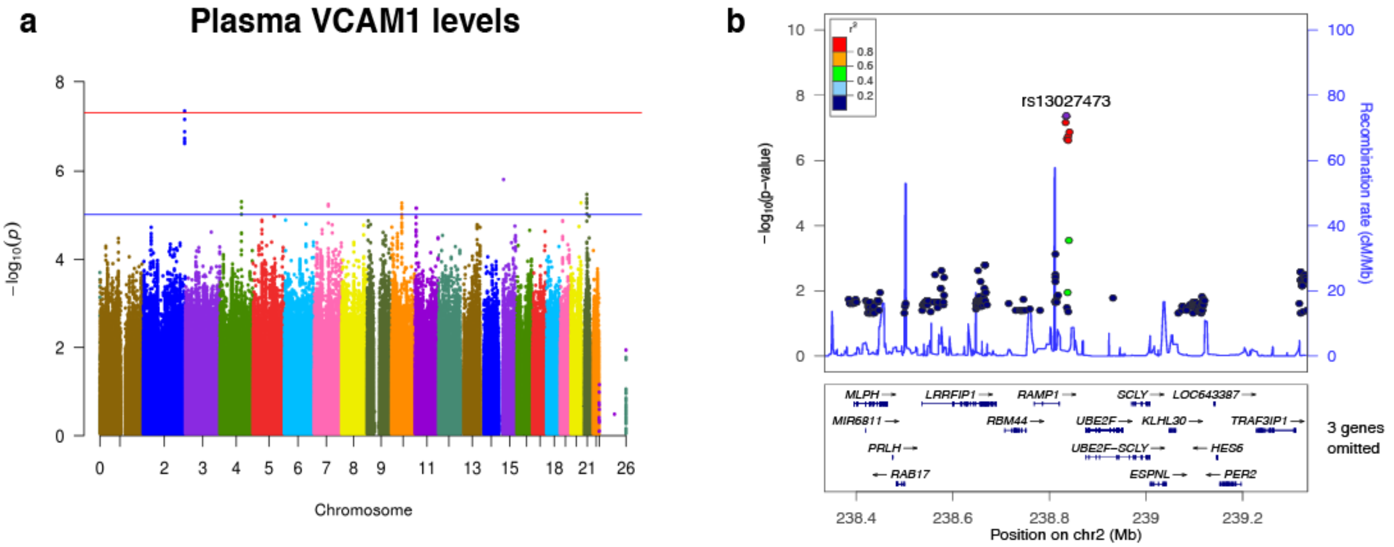


**Supplementary Figure S46. Manhattan and regional plots for associations with plasma levels of VCAM1.** a) Manhattan plot of -$\log_{10}$ p-values for association with plasma levels of VCAM1; b) Regional plot for genome-wide significant association with VCAM1 plasma levels.

**Supplementary Table S1. Comparison of $\log_{10}$ plasma analyte levels to examine difference between cases and controls (mean±SD)**

| Analyte | Case (n=521) | Control (n=297) | p-value |
|---|---|---|---|
| A1Micro | 0.100±0.982 | -0.216±0.941 | 2.20E-06 |
| A2Macro | 0.048±0.899 | -0.145±1.167 | 5.00E-04 |
| AACT | -0.075±0.790 | -0.141±0.830 | 3.30E-02 |
| AAT | 0.024±0.975 | 0.001±0.912 | 4.84E-01 |
| ACE | 0.008±0.938 | -0.000±1.118 | 9.94E-01 |
| Adiponectin | 0.049±0.989 | -0.120±1.018 | 5.00E-03 |
| AFP | 0.024±0.956 | -0.020±1.072 | 9.80E-01 |
| AgRP | 0.056±0.984 | -0.137±0.987 | 6.10E-03 |
| AGT | -0.013±1.013 | -0.059±0.934 | 3.18E-01 |
| ANG2 | 0.068±0.984 | -0.173±1.061 | 4.30E-03 |
| ApoA1 | -0.050±0.981 | 0.051±1.070 | 1.55E-07 |
| ApoA2 | -0.052±0.990 | 0.168±0.998 | 4.00E-03 |
| ApoA4 | 0.016±0.988 | -0.095±1.016 | 5.07E-02 |
| ApoB | 0.045±1.034 | -0.043±0.883 | 5.40E-02 |
| ApoC1 | 0.030±1.021 | -0.082±0.962 | 1.80E-02 |
| ApoC3 | -0.015±1.019 | 0.072±0.962 | 2.83E-01 |
| ApoD | -0.004±1.009 | -0.040±0.949 | 8.98E-01 |
| ApoE | -0.071±0.931 | 0.174±0.916 | 6.32E-03 |
| ApoH | -0.003±0.900 | 0.028±1.313 | 4.68E-01 |
| AXL | 0.064±0.967 | -0.113±1.114 | 2.76E-02 |
| B2M | -0.026±0.916 | -0.095±0.879 | 6.32E-01 |
| BDNF | -0.046±0.978 | -0.089±1.033 | 5.75E-01 |
| BLC | 0.049±0.826 | -0.110±0.883 | 1.90E-02 |
| BMP6 | 0.027±0.892 | 0.039±0.835 | 7.36E-01 |
| BTC | 0.014±0.912 | -0.045±1.113 | 2.69E-01 |
| C-peptide | 0.029±1.008 | -0.042±0.989 | 4.19E-01 |
| C3 | -0.064±1.012 | 0.158±0.923 | 3.00E-03 |
| CA199 | 0.052±1.020 | -0.133±0.940 | 2.62E-02 |
| Calcitonin | 0.042±0.982 | -0.116±1.109 | 4.25E-02 |
| CD40 | 0.013±1.037 | -0.100±0.945 | 3.35E-01 |
| CD40L | -0.054±1.013 | -0.044±0.890 | 1.21E-01 |
| CD5L | 0.014±1.032 | 0.001±0.969 | 4.31E-01 |
| CEA | 0.029±0.969 | -0.109±1.009 | 2.04E-01 |
| CFHR1 | 0.129±0.717 | 0.242±0.707 | 1.83E-01 |
| CgA | 0.007±1.021 | -0.007±0.876 | 1.50E-01 |
| CKMB | 0.058±0.936 | -0.124±1.148 | 4.31E-02 |
| CLU | -0.042±0.962 | 0.038±1.016 | 6.43E-01 |
| CNTF | -0.001±0.967 | -0.029±0.951 | 9.94E-01 |
| Cortisol | 0.052±0.997 | -0.127±1.067 | 5.37E-02 |
| CRP | -0.074±1.008 | 0.135±0.958 | 3.00E-03 |
| CystC | -0.031±0.953 | 0.003±0.951 | 6.70E-02 |

| | | | |
|---|---|---|---|
| EGF | -0.050±1.015 | -0.079±0.873 | 5.60E-01 |
| EGFR | -0.041±0.938 | 0.114±1.041 | 3.69E-01 |
| ENA78 | -0.059±0.988 | -0.048±0.961 | 9.27E-01 |
| Eotaxin-1 | -0.011±1.003 | 0.060±0.993 | 6.51E-02 |
| Eotaxin-3 | 0.060±0.935 | -0.232±1.152 | 7.00E-04 |
| F7 | 0.049±0.796 | 0.156±0.722 | 1.79E-01 |
| FABP | 0.047±1.026 | -0.052±0.947 | 9.06E-01 |
| FAS | -0.007±0.862 | -0.126±0.785 | 1.29E-01 |
| FASL | -0.034±0.855 | -0.001±0.923 | 9.71E-01 |
| Ferritin | -0.024±1.008 | 0.037±0.961 | 1.77E-01 |
| FetuinA | -0.030±1.059 | 0.035±0.877 | 7.57E-01 |
| FGF4 | 0.055±0.922 | -0.121±0.929 | 1.10E-02 |
| Fibrinogen | 0.071±0.458 | 0.029±0.436 | 7.59E-01 |
| FSH | -0.011±1.037 | 0.003±0.875 | 1.33E-01 |
| GH | 0.049±0.995 | -0.065±1.019 | 2.08E-01 |
| GROa | -0.068±0.971 | -0.065±0.946 | 8.29E-01 |
| GSTa | -0.017±1.06 | 0.062±0.841 | 2.64E-01 |
| HBEGF | 0.133±0.751 | -0.112±0.857 | 1.74E-01 |
| HCC4 | 0.017±0.976 | 0.017±0.983 | 7.90E-01 |
| HGF | 0.066±0.985 | -0.176±1.022 | 4.20E-03 |
| HP | -0.028±1.106 | 0.019±0.787 | 4.81E-01 |
| I309 | 0.010±1.009 | 0.001±0.989 | 8.15E-01 |
| ICAM1 | 0.061±0.841 | 0.061±0.784 | 6.37E-01 |
| IgA | -0.023±1.013 | -0.006±1.013 | 5.03E-01 |
| IgE | -0.025±0.949 | 0.007±1.085 | 3.85E-01 |
| IGFBP2 | 0.123±0.978 | -0.249±1.066 | 1.90E-18 |
| IgM | -0.076±1.011 | 0.166±0.978 | 2.00E-04 |
| IL13 | 0.038±1.047 | -0.081±0.881 | 3.74E-02 |
| IL16 | 0.024±0.946 | -0.023±0.898 | 5.51E-01 |
| IL18 | 0.008±1.004 | -0.037±1.001 | 6.97E-01 |
| IL3 | 0.019±1.037 | -0.063±1.037 | 7.04E-02 |
| IL6r | 0.052±0.867 | -0.009±0.933 | 3.75E-01 |
| IL8 | -0.006±1.058 | -0.01±0.839 | 8.17E-01 |
| Insulin | -0.023±1.026 | -0.04±0.857 | 7.00E-01 |
| IP10 | 0.022±0.999 | -0.056±1.031 | 7.92E-01 |
| KIM1 | 0.027±0.982 | -0.095±0.982 | 2.93E-04 |
| Leptin | -0.040±0.920 | 0.128±1.020 | 5.30E-02 |
| LH | -0.019±0.972 | 0.002±1.031 | 4.45E-01 |
| LPa | 0.002±0.999 | -0.018±0.994 | 4.46E-01 |
| MB | 0.023±1.028 | -0.023±0.954 | 3.23E-01 |
| MCP1 | -0.042±0.838 | -0.003±0.764 | 3.00E-01 |
| MCP2 | -0.009±0.761 | -0.187±0.926 | 1.70E-02 |
| MCP3 | 0.019±0.918 | -0.105±1.012 | 5.40E-02 |
| MCP4 | -0.045±0.720 | -0.208±1.107 | 2.79E-01 |
| MCSF | 0.007±0.946 | -0.100±0.946 | 2.36E-01 |

| | | | |
|---|---|---|---|
| MDC | -0.022±0.863 | -0.008±0.887 | 5.56E-01 |
| MIF | -0.048±0.995 | -0.056±0.995 | 1.09E-01 |
| MIG | 0.020±0.976 | -0.079±0.993 | 8.46E-01 |
| MIP1a | 0.048±0.933 | -0.130±1.002 | 5.30E-02 |
| MIP1b | -0.044±0.768 | -0.222±0.680 | 3.70E-02 |
| MIP3a | -0.100±0.824 | -0.019±0.888 | 3.54E-01 |
| MMP1 | 0.038±1.013 | -0.154±0.944 | 4.30E-03 |
| MMP10 | -0.009±1.018 | -0.017±1.018 | 6.15E-02 |
| MMP2 | 0.120±0.574 | -0.080±0.457 | 1.00E-03 |
| MMP7 | 0.038±1.074 | -0.118±0.757 | 5.04E-02 |
| MMP9 | 0.033±0.906 | -0.113±0.776 | 2.30E-02 |
| MMP9tot | 0.071±0.885 | -0.024±0.839 | 2.44E-01 |
| MPIF1 | 0.044±0.871 | -0.156±1.200 | 1.93E-02 |
| MPO | -0.013±1.070 | 0.014±0.823 | 6.38E-01 |
| NGAL | 0.020±1.029 | -0.038±0.920 | 9.17E-01 |
| NrCAM | 0.036±0.688 | -0.130±0.744 | 3.50E-02 |
| NTproBNP | 0.137±0.975 | -0.308±0.877 | 1.83E-29 |
| Osteopontin | 0.079±1.017 | -0.081±0.973 | 4.53E-01 |
| PAI1 | -0.075±0.942 | 0.033±1.102 | 2.27E-01 |
| PAP | 0.025±1.048 | -0.073±0.840 | 5.00E-03 |
| PAPPA | 0.058±1.049 | -0.159±0.845 | 1.83E-01 |
| PARC | -0.059±1.065 | 0.158±0.853 | 8.64E-04 |
| PDGFBB | -0.055±1.034 | -0.064±0.868 | 6.92E-01 |
| PLGF | -0.027±1.107 | 0.121±0.670 | 6.30E-03 |
| PPP | 0.111±1.035 | -0.284±0.861 | 2.98E-10 |
| PRL | 0.016±1.011 | -0.012±1.001 | 8.61E-01 |
| Proins | 0.013±0.991 | -0.094±1.017 | 2.52E-01 |
| Proinst | 0.005±0.983 | -0.092±1.027 | 3.55E-01 |
| ProteinS | 0.001±0.935 | 0.038±0.852 | 9.71E-01 |
| PYY | 0.057±1.033 | -0.158±0.911 | 3.10E-03 |
| RAGE | 0.065±0.994 | -0.081±0.979 | 4.26E-02 |
| RANTES | -0.035±0.971 | -0.112±0.997 | 2.65E-01 |
| Resist | 0.033±0.945 | -0.040±0.836 | 8.70E-01 |
| SAP | -0.041±1.027 | 0.091±0.956 | 3.67E-02 |
| SCF | 0.007±0.973 | -0.176±0.965 | 9.50E-03 |
| SELE | -0.064±0.995 | 0.155±1.006 | 5.00E-03 |
| SGOT | -0.041±1.037 | 0.039±0.827 | 1.61E-01 |
| SHBG | -0.001±0.920 | -0.037±1.078 | 4.46E-01 |
| SOD1 | -0.049±0.976 | -0.032±0.925 | 8.19E-01 |
| Sortilin | 0.026±0.984 | -0.172±1.000 | 1.80E-03 |
| TBG | -0.032±0.982 | 0.005±1.041 | 7.09E-01 |
| TECK | 0.002±0.974 | -0.029±0.980 | 9.44E-01 |
| Testosterone | 0.007±1.042 | -0.007±0.973 | 8.75E-02 |
| TF | 0.048±0.981 | -0.127±0.993 | 9.00E-04 |
| TFF3 | 0.034±0.966 | -0.122±1.051 | 1.84E-20 |

| | | | |
|---|---|---|---|
| THP | -0.006±0.992 | 0.019±1.009 | 9.53E-02 |
| THPO | 0.290±0.798 | 0.109±0.536 | 4.17E-01 |
| TIMP1 | -0.011±0.910 | -0.064±0.884 | 7.65E-01 |
| TM | 0.038±1.006 | -0.095±1.005 | 2.02E-01 |
| TNC | 0.073±0.973 | -0.187±1.069 | 4.00E-04 |
| TNFa | 0.138±0.788 | 0.088±0.848 | 1.31E-01 |
| TNFR2 | -0.015±0.890 | -0.033±0.877 | 6.58E-01 |
| TP1 | -0.005±0.979 | -0.145±1.033 | 6.30E-02 |
| TRAILR3 | 0.019±0.963 | -0.089±0.982 | 2.68E-01 |
| TSH | 0.040±0.975 | -0.019±0.934 | 4.91E-01 |
| TTR | -0.023±1.031 | 0.099±0.940 | 6.51E-02 |
| VCAM1 | 0.028±0.973 | -0.091±0.996 | 2.63E-01 |
| VEGF | 0.004±0.949 | -0.079±0.994 | 4.92E-01 |
| Vitron | -0.021±0.997 | 0.054±0.978 | 2.65E-01 |
| vWF | 0.071±0.939 | -0.144±0.873 | 7.00E-03 |

**Supplementary Table S2. The correlation between the analyte values and age, gender in the combined dataset**

| Analytes | Age p-value | Estimate | Gender p-value | Estimate |
|---|---|---|---|---|
| A1Micro | 7.59E-09 | 0.025 | 9.64E-04 | -0.204 |
| A2Macro | 1.29E-02 | 0.015 | 6.08E-12 | 0.465 |
| AACT | 3.36E-02 | 0.007 | 3.82E-04 | 0.212 |
| AAT | 1.90E-01 | 0.007 | 2.84E-06 | 0.314 |
| ACE | 4.76E-01 | 0.003 | 9.50E-01 | -0.013 |
| Adiponectin | 2.73E-05 | 0.021 | 7.27E-23 | 0.671 |
| AFP | 3.83E-02 | 0.009 | 1.70E-01 | -0.098 |
| AgRP | 4.85E-01 | 0.005 | 9.99E-01 | -0.021 |
| AGT | 6.55E-01 | -0.001 | 3.56E-01 | 0.061 |
| ANG2 | 1.81E-12 | 0.033 | 2.65E-02 | 0.183 |
| ApoA1 | 1.37E-01 | 0.007 | 1.18E-31 | 0.826 |
| ApoA2 | 3.80E-02 | -0.009 | 6.32E-08 | 0.352 |
| ApoA4 | 6.71E-01 | 0.000 | 8.59E-02 | 0.105 |
| ApoB | 1.91E-04 | -0.015 | 6.19E-02 | 0.095 |
| ApoC1 | 4.96E-02 | -0.005 | 2.37E-19 | 0.586 |
| ApoC3 | 9.34E-01 | 0.002 | 1.03E-12 | 0.478 |
| ApoD | 4.51E-03 | 0.010 | 6.54E-05 | -0.251 |
| ApoE | 3.10E-02 | 0.010 | 3.57E-06 | 0.324 |
| ApoH | 4.37E-01 | 0.002 | 3.16E-01 | -0.064 |
| AXL | 5.38E-02 | 0.010 | 9.20E-01 | -0.001 |
| B2M | 2.47E-24 | 0.038 | 1.37E-01 | -0.034 |
| BDNF | 3.00E-01 | 0.006 | 3.34E-04 | 0.250 |
| BLC | 5.04E-16 | 0.029 | 5.60E-01 | 0.072 |
| BMP6 | 5.63E-01 | -0.001 | 3.33E-01 | 0.053 |
| BTC | 6.56E-01 | 0.002 | 1.07E-01 | 0.125 |
| C-peptide | 8.12E-02 | 0.007 | 3.08E-03 | -0.195 |
| C3 | 5.02E-02 | -0.009 | 9.24E-03 | 0.179 |
| CA199 | 3.68E-02 | 0.009 | 9.25E-01 | -0.012 |
| Calcitonin | 6.70E-01 | -0.003 | 5.88E-09 | -0.443 |
| CD40 | 1.47E-22 | 0.042 | 5.02E-02 | 0.175 |
| CD40L | 8.90E-01 | -0.001 | 2.21E-05 | 0.302 |
| CD5L | 2.56E-09 | 0.024 | 8.98E-03 | -0.155 |
| CEA | 1.74E-04 | 0.018 | 4.93E-01 | -0.061 |
| CFHR1 | 4.32E-01 | 0.001 | 1.86E-02 | 0.145 |
| CgA | 2.90E-20 | 0.037 | 6.08E-03 | -0.131 |
| CKMB | 9.63E-05 | 0.019 | 9.38E-08 | -0.386 |
| CLU | 3.64E-01 | -0.003 | 1.09E-11 | 0.454 |
| CNTF | 6.16E-01 | 0.003 | 5.78E-01 | 0.029 |
| Cortisol | 2.68E-03 | 0.014 | 9.39E-02 | -0.106 |
| CRP | 9.41E-02 | 0.007 | 3.12E-05 | 0.308 |
| CystC | 5.35E-24 | 0.039 | 7.46E-02 | -0.055 |

| | | | | |
|---|---|---|---|---|
| EGF | 8.08E-01 | 0.000 | 6.84E-04 | 0.234 |
| EGFR | 2.24E-13 | -0.031 | 3.25E-06 | 0.284 |
| ENA78 | 9.54E-01 | 0.002 | 1.07E-07 | 0.366 |
| Eotaxin-1 | 1.32E-05 | 0.017 | 2.65E-02 | -0.129 |
| Eotaxin-3 | 1.96E-07 | 0.026 | 9.56E-01 | 0.011 |
| F7 | 2.47E-01 | -0.003 | 7.01E-10 | 0.339 |
| FABP | 6.90E-17 | 0.034 | 1.20E-05 | -0.246 |
| FAS | 6.22E-08 | 0.020 | 2.32E-03 | -0.149 |
| FASL | 9.81E-02 | -0.005 | 8.58E-03 | 0.146 |
| Ferritin | 4.78E-01 | -0.001 | 8.17E-16 | -0.539 |
| FetuinA | 2.09E-03 | -0.013 | 3.82E-02 | 0.133 |
| FGF4 | 1.63E-01 | 0.007 | 8.19E-01 | -0.021 |
| Fibrinogen | 2.39E-03 | 0.008 | 1.33E-02 | 0.071 |
| FSH | 1.97E-05 | 0.019 | 5.69E-195 | 1.600 |
| GH | 2.51E-06 | 0.025 | 3.39E-08 | 0.402 |
| GROa | 7.54E-01 | 0.000 | 1.26E-03 | 0.214 |
| GSTa | 1.07E-01 | -0.008 | 8.24E-01 | 0.027 |
| HBEGF | 6.02E-09 | 0.026 | 4.64E-01 | -0.072 |
| HCC4 | 2.48E-01 | 0.004 | 6.29E-03 | -0.160 |
| HGF | 3.01E-11 | 0.029 | 7.18E-03 | -0.147 |
| HP | 2.69E-02 | 0.010 | 8.85E-03 | 0.219 |
| I309 | 2.54E-02 | 0.011 | 2.10E-01 | 0.078 |
| ICAM1 | 5.04E-02 | 0.007 | 1.28E-02 | 0.151 |
| IgA | 1.40E-02 | 0.010 | 2.27E-01 | -0.071 |
| IgE | 6.90E-01 | -0.005 | 1.50E-10 | -0.444 |
| IGFBP2 | 8.79E-19 | 0.041 | 6.77E-01 | 0.067 |
| IgM | 2.19E-01 | 0.004 | 1.32E-02 | 0.172 |
| IL13 | 2.65E-02 | -0.008 | 8.16E-03 | 0.156 |
| IL16 | 6.42E-10 | 0.025 | 8.80E-01 | 0.017 |
| IL18 | 7.79E-01 | -0.001 | 5.76E-06 | -0.312 |
| IL3 | 3.19E-01 | 0.005 | 7.12E-02 | 0.131 |
| IL6r | 6.49E-01 | 0.003 | 2.99E-02 | 0.129 |
| IL8 | 6.10E-06 | 0.020 | 1.08E-01 | 0.138 |
| Insulin | 9.25E-01 | 0.001 | 5.85E-01 | 0.037 |
| IP10 | 2.45E-11 | 0.031 | 2.68E-01 | 0.110 |
| KIM1 | 5.92E-04 | 0.017 | 3.33E-01 | 0.070 |
| Leptin | 2.94E-01 | 0.000 | 1.93E-40 | 0.838 |
| LH | 1.49E-06 | 0.021 | 1.48E-155 | 1.494 |
| LPa | 4.88E-01 | -0.003 | 9.44E-02 | 0.128 |
| MB | 5.72E-16 | 0.030 | 3.85E-19 | -0.547 |
| MCP1 | 6.43E-03 | 0.009 | 2.24E-01 | 0.073 |
| MCP2 | 5.52E-05 | 0.017 | 1.86E-02 | 0.149 |
| MCP3 | 3.06E-01 | 0.005 | 8.19E-01 | -0.022 |
| MCP4 | 4.20E-03 | 0.014 | 7.69E-01 | -0.014 |
| MCSF | 4.14E-01 | 0.004 | 6.89E-01 | 0.030 |

| | | | | |
|---|---|---|---|---|
| MDC | 1.05E-01 | 0.007 | 8.16E-10 | 0.386 |
| MIF | 1.03E-01 | 0.008 | 7.98E-01 | 0.018 |
| MIG | 1.15E-45 | 0.059 | 1.57E-04 | 0.285 |
| MIP1a | 8.43E-15 | 0.032 | 1.59E-03 | -0.146 |
| MIP1b | 1.24E-04 | 0.015 | 7.36E-01 | -0.024 |
| MIP3a | 1.43E-01 | 0.004 | 1.17E-01 | 0.116 |
| MMP1 | 8.31E-01 | 0.003 | 5.90E-01 | 0.019 |
| MMP10 | 9.34E-03 | 0.013 | 5.83E-01 | -0.042 |
| MMP2 | 7.30E-14 | 0.020 | 2.36E-01 | -0.044 |
| MMP7 | 1.45E-07 | 0.025 | 1.04E-02 | 0.176 |
| MMP9 | 3.72E-01 | -0.001 | 4.93E-01 | 0.022 |
| MMP9tot | 6.06E-01 | 0.003 | 9.53E-01 | -0.015 |
| MPIF1 | 6.99E-05 | 0.019 | 7.81E-01 | 0.015 |
| MPO | 4.83E-01 | 0.003 | 7.20E-01 | 0.024 |
| NGAL | 2.47E-09 | 0.023 | 8.61E-05 | -0.244 |
| NrCAM | 8.12E-02 | 0.009 | 8.44E-02 | 0.058 |
| NTproBNP | 1.15E-27 | 0.047 | 9.55E-02 | 0.129 |
| Osteopontin | 3.47E-23 | 0.043 | 3.79E-03 | -0.154 |
| PAI1 | 2.04E-01 | -0.006 | 2.33E-02 | 0.164 |
| PAP | 2.42E-01 | 0.005 | 5.34E-28 | -0.761 |
| PAPPA | 2.79E-03 | 0.014 | 2.44E-07 | -0.367 |
| PARC | 3.79E-07 | 0.020 | 5.34E-01 | 0.003 |
| PDGFBB | 7.44E-01 | -0.001 | 7.87E-03 | 0.188 |
| PLGF | 1.13E-03 | 0.013 | 7.37E-01 | 0.047 |
| PPP | 3.04E-11 | 0.031 | 8.50E-02 | -0.094 |
| PRL | 4.25E-05 | 0.018 | 4.51E-01 | 0.072 |
| Proins | 1.94E-03 | 0.012 | 1.78E-06 | -0.318 |
| Proinst | 1.14E-03 | 0.013 | 3.84E-05 | -0.275 |
| ProteinS | 9.24E-02 | -0.007 | 2.48E-02 | 0.137 |
| PYY | 2.90E-01 | 0.007 | 8.03E-01 | 0.007 |
| RAGE | 3.19E-01 | 0.008 | 3.51E-02 | 0.145 |
| RANTES | 3.64E-01 | 0.006 | 4.78E-04 | 0.241 |
| Resist | 3.81E-07 | 0.020 | 4.51E-02 | -0.114 |
| SAP | 3.15E-01 | -0.009 | 5.16E-14 | -0.509 |
| SCF | 4.32E-04 | 0.017 | 1.27E-02 | 0.183 |
| SELE | 3.86E-02 | -0.010 | 6.82E-02 | 0.115 |
| SGOT | 2.14E-01 | 0.004 | 2.22E-01 | -0.057 |
| SHBG | 4.99E-07 | 0.023 | 1.16E-28 | 0.742 |
| SOD1 | 6.29E-01 | 0.002 | 2.24E-01 | 0.082 |
| Sortilin | 1.63E-04 | 0.020 | 8.59E-06 | 0.320 |
| TBG | 6.29E-03 | -0.010 | 1.34E-12 | 0.482 |
| TECK | 4.06E-02 | 0.008 | 2.14E-02 | -0.145 |
| Testosterone | 9.50E-05 | -0.018 | 4.04E-176 | -1.609 |
| TF | 2.92E-03 | -0.010 | 1.13E-05 | 0.281 |
| TFF3 | 4.15E-19 | 0.042 | 6.03E-04 | 0.236 |

| | | | | |
|---|---|---|---|---|
| THP | 1.56E-04 | -0.018 | 2.12E-05 | 0.305 |
| THPO | 3.82E-02 | 0.015 | 7.27E-03 | 0.073 |
| TIMP1 | 1.31E-17 | 0.032 | 6.88E-01 | 0.028 |
| TM | 2.40E-05 | 0.017 | 2.70E-07 | -0.330 |
| TNC | 2.32E-02 | 0.012 | 1.61E-01 | 0.086 |
| TNFa | 3.54E-02 | 0.007 | 6.83E-01 | 0.003 |
| TNFR2 | 2.43E-18 | 0.032 | 1.28E-01 | -0.038 |
| TP1 | 5.82E-01 | 0.004 | 3.30E-02 | 0.147 |
| TRAILR3 | 1.49E-06 | 0.021 | 6.65E-01 | -0.012 |
| TSH | 3.85E-01 | 0.005 | 4.79E-01 | 0.021 |
| TTR | 2.78E-02 | -0.013 | 2.31E-05 | -0.304 |
| VCAM1 | 1.98E-11 | 0.029 | 9.20E-01 | 0.037 |
| VEGF | 1.45E-15 | 0.032 | 5.90E-01 | 0.078 |
| Vitron | 2.15E-02 | -0.008 | 3.83E-02 | 0.131 |
| vWF | 4.96E-08 | 0.024 | 5.03E-02 | 0.132 |

**Supplementary Table S3. Genome-wide significant results from joint GWAS in ADNI or KADRC GWAS. Highlighted results were excluded due to inconsistency in association between studies. λ=genomic inflation estimate.**

| | | | | ADNI | | KADRC | | Joint | | Meta | Previously | Other traits associated with loci or gene |
|---|---|---|---|---|---|---|---|---|---|---|---|---|
| **Analyte** | **SNP** | **Gene** | **λ** | **p-value** | **beta** | **p-value** | **beta** | **p-value** | **beta** | **p-value** | **reported** | **(based on NHGRI catalog)** |
| AAT | rs926144 | *SERPINA1* | 1.009 | 2.80E-07 | -0.381 | 3.73E-06 | -0.446 | 4.71E-12 | -0.405 | 5.38E-12 | novel | Metabolite levels |
| ACE | rs4343 | *ACE* | 1.029 | 5.03E-25 | 0.562 | 5.52E-21 | 0.845 | 6.66E-44 | 0.493 | 4.11E-44 | known | Metabolite levels; Metabolic traits; Angiotensin-converting enzyme activity |
| ACE | rs2519093 | *ABO* | 1.029 | 2.63E-05 | -0.289 | 1.65E-04 | -0.459 | 1.90E-08 | -0.352 | 1.78E-08 | *known | Lipid traits; Coronary artery disease; Ischemic stroke; Large artery stroke; Serum alkaline phosphatase levels; Malaria; Venous thromboembolism; Graves' disease; Thyroid hormone levels; Tumor biomarkers; End-stage coagulation; Coagulation factor levels; Red blood cell traits; Obesity-related traits; Activated partial thromboplastin time; Duodenal ulcer; Inflammatory biomarkers; Liver enzyme levels; Metabolic traits; Soluble ICAM-1; D-dimer levels; Phytosterol levels; E-selectin levels; Soluble levels of adhesion molecules; Hematological and biochemical traits; mean corpuscular hemoglobin concentration; Angiotensin-converting enzyme activity; Pancreatic cancer; vWF and FVIII levels |
| AGT | rs35837081 | *AGT* | 1.021 | 3.82E-17 | 0.799 | 4.70E-21 | 1.066 | 4.45E-34 | 0.890 | 2.17E-35 | known | AGT levels |
| ANG2 | rs3747214 | *PARVG* | 1.000 | 2.92E-03 | 0.185 | 3.63E-07 | 0.415 | 2.21E-08 | 0.277 | 4.13E-08 | novel | - |
| ApoA4 | rs1263167 | *APOA4* | 1.000 | 6.95E-30 | -0.873 | 1.19E-27 | -0.978 | 2.64E-54 | -0.919 | 2.89E-55 | known | Hypertriglyceridemia; Coronary heart disease; HDL cholesterol; LDL cholesterol; Triglycerides; total cholesterol; Hematological and biochemical traits |

| | | | | | | | | | | | | |
|---|---|---|---|---|---|---|---|---|---|---|---|---|
| ApoE | rs769449 | *APOE* | 1.043 | 7.58E-14 | -0.512 | 4.05E-15 | -0.755 | 2.76E-26 | -0.594 | 6.64E-27 | known | LDL cholesterol; Alzheimer's disease; HDL cholesterol; C-reactive protein; Age-related macular degeneration; Cholesterol, total; Alzheimer's disease biomarkers; Brain imaging; Triglycerides; Quantitative traits; Apolipoprotein Levels; Metabolite levels; Cardiovascular disease risk factors; Lipid traits; Response to statin therapy (LDL-C); Lipid metabolism phenotypes |
| ApoH | rs2873966 | *APOH* | 1.030 | 5.34E-12 | 0.403 | 1.26E-05 | 0.464 | 3.37E-15 | 0.428 | 4.60E-16 | known | Blood pressure measurement (high sodium and potassium intervention); LDL cholesterol; B2-Glycoprotein I plasma levels |
| ApoH | rs117842936 | *MBD2* | 1.030 | 1.15E-02 | -0.309 | 6.34E-11 | -1.430 | 1.03E-10 | -0.727 | 1.79E-09 | novel | Periodontitis |
| ApoH | rs148521708 | *TJP2* | 1.030 | 7.76E-03 | -0.338 | 7.92E-10 | -1.428 | 2.64E-10 | -0.742 | 4.31E-09 | novel | Refractive error; Renal sinus fat |
| BLC | rs7541151 | *DDAH1* | 1.004 | 4.44E-05 | -0.674 | 3.52E-05 | -0.807 | 6.44E-09 | -0.720 | 8.13E-09 | novel | Serum dimethylarginine levels; Multiple sclerosis |
| CA19-9 | rs485073 | *FUT2* | 1.000 | 2.03E-15 | -0.534 | 1.86E-09 | -0.453 | 2.12E-23 | -0.509 | 2.35E-23 | known | Tumor biomarkers; Vitamin B12 levels; Bipolar disorder; Retinal vascular caliber; Liver enzyme levels (alkaline phosphatase); Crohn's disease; Cholesterol, total; Metabolic traits; Obesity-related traits; Liver enzyme levels (gamma-glutamyl transferase); Folate pathway vitamin levels; Homocysteine levels |
| CD40 | rs6032660 | *CD40* | 1.000 | 1.60E-12 | -0.438 | 3.13E-10 | -0.530 | 9.81E-21 | -0.463 | 3.55E-21 | known | Inflammatory bowel disease; Kawasaki disease; Rheumatoid arthritis; Multiple sclerosis |
| CD5L | rs2765501 | *CD5L* | 1.000 | 2.34E-07 | 0.339 | 7.62E-04 | 0.272 | 9.97E-10 | 0.309 | 7.98E-10 | known | CD6; CD5; PTGDR2 |

| | | | | | | | | | | | | | |
|---|---|---|---|---|---|---|---|---|---|---|---|---|---|
| CEA | rs570794 | *FUT2* | 1.000 | 6.33E-09 | -0.378 | 7.23E-10 | -0.486 | 2.84E-17 | -0.424 | 5.49E-17 | known | Tumor biomarkers; Vitamin B12 levels; Bipolar disorder; Retinal vascular caliber; Liver enzyme levels (alkaline phosphatase); Crohn's disease; Cholesterol, total; Metabolic traits; Obesity-related traits; Liver enzyme levels (gamma-glutamyl transferase); Folate pathway vitamin levels; Homocysteine levels |
| CEA | rs12468845 | *AFF3* | 1.000 | 1.10E-05 | -0.746 | 2.22E-04 | -1.287 | 3.88E-08 | -0.834 | 2.16E-08 | novel | - |
| CFHR1 | rs12144939 | *CFH* | 1.000 | 9.44E-89 | -1.077 | 7.60E-55 | -1.162 | 8.99E-143 | -1.108 | 1.11E-141 | known | Age-related macular degeneration; Circulating myeloperoxidase levels (serum); Complement C3 and C4 levels; Nephropathy Meningococcal disease |
| CRP | rs12972156 | *PVRL2* | 1.000 | 1.65E-05 | -0.308 | 1.58E-07 | -0.565 | 9.93E-11 | -0.383 | 4.00E-11 | known | Alzheimer's disease biomarkers; Age-related macular degeneration; HDL cholesterol; Alzheimer's disease |
| CystC | rs13039144 | *CST3* | 1.008 | 1.20E-06 | -0.356 | 1.48E-03 | -0.337 | 1.23E-08 | -0.347 | 7.42E-09 | known | Chronic kidney disease; Cystatin C |
| ENA78 | rs409336 | *CXCL5* | 1.000 | 4.14E-07 | 0.453 | 5.00E-03 | 0.326 | 1.11E-08 | 0.405 | 1.10E-08 | novel | Inflammatory bowel disease; Metabolite levels |
| F7 | rs10665 | *MCF2L* | 1.014 | 1.81E-16 | -0.609 | 1.38E-11 | -0.570 | 1.44E-26 | -0.598 | 1.74E-26 | known | Osteoarthritis; Factor VII |
| F7 | rs11594693 | *WDR11* | 1.014 | 1.30E-05 | 0.245 | 1.89E-03 | 0.212 | 3.72E-08 | 0.239 | 8.89E-08 | novel | - |
| FetuinA | rs2070633 | *AHSG* | 1.000 | 1.91E-28 | -0.662 | 5.92E-17 | -0.567 | 2.88E-44 | -0.629 | 9.94E-44 | known | Fetuin-A levels; Activated partial thromboplastin time |
| FGF4 | rs13117858 | *GALNTL6* | 1.011 | 1.17E-07 | 0.457 | 5.93E-04 | 0.340 | 3.12E-10 | 0.410 | 2.90E-10 | novel | - |
| GROa | rs1263549 | *PTPRN2* | 1.001 | 1.22E-05 | 0.698 | 8.29E-04 | 0.650 | 2.16E-08 | 0.685 | 3.71E-08 | novel | Myopia (pathological); Response to amphetamines; Bipolar disorder and schizophrenia; Obesity-related traits |
| GSTa | rs9395826 | *GSTA1* | 1.000 | 7.91E-09 | -0.426 | 2.44E-03 | -0.288 | 8.19E-11 | -0.396 | 7.21E-11 | known | GSTa levels |
| HCC4 | rs80329614 | *CCL16* | 1.010 | 9.29E-15 | -0.977 | 4.90E-16 | -0.948 | 1.16E-27 | -0.958 | 1.21E-28 | known | HCC4 levels |
| HP | rs138433199 | *SPRY4* | 1.031 | 7.56E-09 | -1.294 | 9.69E-02 | -0.315 | 6.49E-09 | -0.900 | 7.56E-09 | novel | Testicular germ cell tumor; Inflammatory bowel disease |
| HP | rs72787038 | *DHODH* | 1.031 | 4.64E-09 | 0.522 | 5.53E-07 | 0.401 | 9.69E-14 | 0.468 | 1.35E-14 | known | Attention deficit hyperactivity disorder and conduct disorder |
| IL13 | rs7433647 | *UBE2E2* | 1.003 | 1.49E-05 | -0.449 | 3.90E-04 | -0.394 | 1.21E-08 | -0.438 | 2.19E-08 | novel | Psychosis (atypical); Type 2 diabetes |
| IL16 | rs11556218 | *IL16* | 1.000 | 4.33E-18 | -1.101 | 1.48E-16 | -0.970 | 6.02E-32 | -1.064 | 9.43E-33 | known | Inattentive symptoms |

| | | | | | | | | | | | | | |
|---|---|---|---|---|---|---|---|---|---|---|---|---|---|
| IL18 | rs146245376 | *MIR3169* | 1.000 | 1.77E-05 | -0.565 | 4.17E-04 | -0.558 | 3.06E-08 | -0.559 | 2.76E-08 | novel | - |
| IL6r | rs12126142 | *IL6R* | 1.000 | 3.56E-69 | 0.843 | 4.45E-39 | 0.860 | 1.81E-106 | 0.850 | 2.48E-106 | known | IL6r levels; Asthma; C-reactive protein; Protein quantitative trait loci; Pulmonary function; Fibrinogen |
| IL8 | rs11889675 | *TAF1B* | 1.009 | 1.48E-07 | 0.481 | 4.36E-02 | 0.185 | 4.60E-08 | 0.364 | 7.47E-08 | novel | - |
| Leptin | rs2031468 | *GLRX3* | 1.000 | 4.06E-06 | -0.250 | 9.66E-04 | -0.242 | 1.04E-08 | -0.250 | 1.48E-08 | novel | HIV-1 susceptibility |
| LPa | rs783147 | *PLG* | 1.000 | 4.05E-08 | -0.353 | 8.27E-03 | -0.192 | 1.96E-09 | -0.290 | 2.72E-09 | known | Aging; Lp(a) levels |
| MCP2 | rs1133763 | *CCL8* | 1.015 | 1.99E-15 | -0.523 | 1.77E-02 | -0.255 | 3.54E-13 | -0.419 | 1.02E-14 | known | - |
| MCSF | rs73741236 | *CTNND2* | 1.008 | 7.69E-04 | -0.586 | 4.03E-06 | -1.277 | 2.08E-08 | -0.841 | 3.98E-08 | novel | Amyotrophic lateral sclerosis (sporadic); Myopia (pathological) |
| MCSF | rs111494896 | *GFRA2* | 1.008 | 1.80E-05 | -0.658 | 4.59E-04 | -0.752 | 2.77E-08 | -0.695 | 3.08E-08 | novel | Neuropathic pain in type 2 diabetes; Migraine with aura |
| MIP1a | rs2015086 | *CCL18* | 1.026 | 1.47E-08 | 0.459 | 6.66E-08 | 0.516 | 2.56E-15 | 0.490 | 6.72E-15 | novel | Higher macrophage expression of CCL18 in human carotid atherosclerotic plaques |
| MIP1b | rs145617407 | *CCR3* | 1.000 | 1.22E-07 | 0.384 | 5.66E-04 | 0.293 | 2.58E-10 | 0.348 | 3.13E-10 | novel | MCP1 levels; Obesity-related traits; Monocyte chemoattractant protein-1; Celiac disease |
| MIP1b | rs4796217 | *CCL4L2* | 1.000 | 7.04E-05 | -0.220 | 1.37E-05 | -0.263 | 1.19E-08 | -0.234 | 6.37E-09 | known | MIP1b levels |
| MMP7 | rs9753755 | *MACROD2* | 1.000 | 1.02E-07 | 1.160 | 1.53E-05 | 0.591 | 8.87E-11 | 0.913 | 6.98E-12 | novel | Eating disorders; Brain connectivity; Presence of antiphospholipid antibodies; Hypertension; Obesity-related traits; Non-alcoholic fatty liver disease histology (other); Autism |
| MPIF1 | rs12154780 | *NAPEPLD* | 1.022 | 3.24E-01 | 0.209 | 2.99E-11 | 2.153 | 2.23E-10 | 1.159 | 2.99E-11 | novel | - |
| MPIF1 | rs17093050 | *NGDN* | 1.022 | 9.49E-01 | 0.036 | 1.95E-04 | 0.624 | 3.49E-08 | 0.642 | 1.95E-04 | novel | - |
| MPIF1 | rs861273 | *CCL23* | 1.022 | 2.64E-12 | -0.429 | 1.41E-04 | -0.683 | 6.37E-14 | -0.455 | 1.69E-15 | known | MPIF1 levels; Pulmonary function |
| MPIF1 | rs72752381 | *CDH6* | 1.022 | 1.20E-02 | 0.352 | 1.04E-07 | 1.431 | 1.37E-08 | 0.767 | 1.44E-07 | novel | Response to methotrexate in juvenile idiopathic arthritis; Liver enzyme levels (gamma-glutamyl transferase); Emphysema-related traits |
| NrCAM | rs10487851 | *NRCAM* | 1.019 | 3.53E-08 | 0.252 | 6.15E-04 | 0.236 | 3.01E-10 | 0.242 | 1.11E-10 | known | NRCAM levels; Femoral neck bone geometry and menarche (age at onset); Coffee consumption |

| | | | | | | | | | | | | |
|---|---|---|---|---|---|---|---|---|---|---|---|---|
| RAGE | rs2070600 | *AGER* | 1.003 | 5.42E-05 | -0.695 | 1.95E-08 | -0.941 | 1.86E-11 | -0.814 | 3.06E-11 | novel | Normal glucose metabolism; Impaired glucose metabolism; Type 2 diabetes mellitus; Chronic obstructive pulmonary disease; Prostate cancer; Pulmonary function |
| RAGE | rs4953649 | *FSHR* | 1.003 | 3.19E-08 | 0.365 | 2.30E-02 | 0.183 | 1.66E-08 | 0.288 | 9.04E-09 | novel | Response to anti-retroviral therapy (ddI/d4T) in HIV-1 infection (Grade 3 peripheral neuropathy); Adverse response to chemotherapy (neutropenia/leucopenia) (etoposide); Polycystic ovary syndrome; Erectile dysfunction and prostate cancer treatment, Radiation response |
| SELE | rs507666 | *ABO* | 1.026 | 4.52E-32 | -0.809 | 3.81E-23 | -1.018 | 1.01E-52 | -0.882 | 1.89E-53 | known | Lipid traits; Coronary artery disease; Ischemic stroke; Large artery stroke; Serum alkaline phosphatase levels; Malaria; Venous thromboembolism; Graves' disease; Thyroid hormone levels; Tumor biomarkers; End-stage coagulation; Coagulation factor levels; Red blood cell traits; Obesity-related traits; Activated partial thromboplastin time; Duodenal ulcer; Inflammatory biomarkers; Liver enzyme levels; Metabolic traits; Soluble ICAM-1; D-dimer levels; Phytosterol levels; E-selectin levels; Soluble levels of adhesion molecules; Hematological and biochemical traits; mean corpuscular hemoglobin concentration; Angiotensin-converting enzyme activity; Pancreatic cancer; vWF and FVIII levels |

| | | | | | | | | | | | | |
|---|---|---|---|---|---|---|---|---|---|---|---|---|
| Sortilin | rs646776 | *CELSR2* | 1.003 | 7.82E-06 | 0.316 | 2.63E-05 | 0.376 | 2.20E-09 | 0.331 | 9.84E-10 | novel | Coronary heart disease; LDL cholesterol; Metabolite levels; Cholesterol, total; Progranulin levels; Response to statin therapy; Lipid metabolism phenotypes; Myocardial infarction (early onset); Cardiovascular disease risk factors; Lipoprotein-associated phospholipase A2 activity and mass |
| TECK | rs72927542 | *ST8SIA3* | 1.000 | 1.21E-05 | 0.669 | 1.35E-03 | 0.637 | 2.26E-08 | 0.679 | 5.91E-08 | novel | - |
| TF | rs6762415 | *TF* | 1.000 | 2.85E-11 | -0.430 | 5.27E-05 | -0.329 | 1.17E-14 | -0.390 | 9.46E-15 | known | Transferrin levels; Alcohol consumption (transferrin glycosylation); Iron status biomarkers; Hepcidin levels; Iron levels |
| TFF3 | rs2444229 | *MIR4790* | 1.025 | 4.60E-05 | 0.290 | 2.98E-05 | 0.352 | 8.33E-09 | 0.311 | 7.32E-09 | novel | - |
| THP | rs12934455 | *UMOD* | 1.006 | 1.23E-24 | -0.854 | 8.54E-19 | -0.882 | 2.80E-42 | -0.871 | 1.08E-41 | known | Femoral neck bone geometry; Hypertension; Chronic kidney disease and serum creatinine levels; Chronic kidney disease; Renal function and chronic kidney disease |
| THPO | rs2279191 | *DHX8* | 1.000 | 1.13E-06 | 0.368 | 6.94E-03 | 0.058 | 3.83E-08 | 0.239 | 6.22E-08 | novel | - |
| TNC | rs2685421 | *SCARA5* | 1.010 | 1.13E-06 | 0.335 | 1.36E-05 | 0.397 | 5.27E-11 | 0.358 | 7.40E-11 | novel | Adverse response to chemotherapy (neutropenia/leucopenia) (cisplatin) |
| VCAM1 | rs13027473 | *RAMP1* | 1.045 | 7.05E-05 | -0.317 | 1.60E-04 | -0.389 | 4.53E-08 | -0.345 | 4.85E-08 | novel | Obesity-related traits |

**Supplementary Table S4. Genome-wide significant associations stratified by AD status.**

| | | Joint | | Cases (n=521) | | Controls (n=297) | |
|---|---|---|---|---|---|---|---|
| **Analyte** | **SNP** | **p-value** | **beta** | **p-value** | **beta** | **p-value** | **beta** |
| AAT | rs926144 | 4.71E-12 | -0.405 | 2.26E-06 | -0.358 | 1.06E-07 | -0.492 |
| ACE | rs4343 | 6.66E-44 | 0.669 | 1.18E-26 | 0.609 | 4.53E-19 | 0.778 |
| ACE | rs2519093 | 1.90E-08 | -0.352 | 1.71E-05 | -0.312 | 1.75E-04 | -0.439 |
| AGT | rs35837081 | 4.45E-34 | 0.890 | 2.40E-18 | 0.816 | 1.04E-17 | 0.998 |
| ANG2 | rs3747214 | 2.21E-08 | 0.277 | 7.38E-04 | 0.207 | 4.40E-06 | 0.383 |
| ApoA4 | rs1263167 | 2.64E-54 | -0.919 | 1.04E-29 | -0.881 | 9.91E-27 | -0.970 |
| ApoE | rs769449 | 2.76E-26 | -0.594 | 1.46E-15 | -0.510 | 2.22E-14 | -0.890 |
| ApoH | rs2873966 | 3.37E-15 | 0.428 | 2.08E-11 | 0.399 | 1.12E-05 | 0.463 |
| ApoH | rs17690171 | 1.02E-15 | -0.511 | 2.61E-09 | -0.415 | 1.34E-07 | -0.650 |
| ApoH | rs8178828 | 6.84E-13 | -0.714 | 2.77E-14 | -0.768 | 1.02E-02 | -0.572 |
| ApoH | rs117842936 | 1.03E-10 | -0.727 | 3.20E-02 | -0.264 | 1.99E-12 | -1.533 |
| ApoH | rs148521708 | 2.64E-10 | -0.742 | 1.54E-03 | -0.398 | 2.12E-08 | -1.341 |
| BLC | rs7541151 | 6.44E-09 | -0.720 | 4.00E-06 | -0.672 | 5.84E-04 | -0.806 |
| CA19-9 | rs485073 | 2.12E-23 | -0.509 | 3.13E-17 | -0.548 | 2.90E-07 | -0.425 |
| CA19-9 | rs112313064 | 7.46E-23 | -0.507 | 1.50E-23 | -0.637 | 2.94E-03 | -0.260 |
| CA19-9 | rs2306969 | 6.11E-23 | -0.575 | 2.16E-20 | -0.678 | 6.11E-05 | -0.383 |
| CD40 | rs6032660 | 9.81E-21 | -0.463 | 9.33E-15 | -0.468 | 1.84E-07 | -0.458 |
| CD5L | rs2765501 | 9.97E-10 | 0.309 | 5.46E-08 | 0.355 | 4.24E-03 | 0.231 |
| CEA | rs570794 | 2.84E-17 | -0.424 | 1.83E-10 | -0.405 | 5.74E-08 | -0.446 |
| CEA | rs12468845 | 3.88E-08 | -0.834 | 2.20E-05 | -0.772 | 2.49E-04 | -1.008 |
| CFHR1 | rs12144939 | 8.99E-143 | -1.108 | 2.76E-89 | -1.081 | 2.49E-54 | -1.162 |
| CRP | rs12972156 | 9.93E-11 | -0.383 | 4.04E-07 | -0.350 | 3.73E-05 | -0.495 |
| CystC | rs13039144 | 1.23E-08 | -0.347 | 1.50E-04 | -0.301 | 8.26E-06 | -0.425 |
| ENA78 | rs409336 | 1.11E-08 | 0.405 | 3.51E-05 | 0.394 | 1.89E-04 | 0.395 |
| F7 | rs10665 | 1.44E-26 | -0.598 | 3.59E-17 | -0.626 | 3.90E-11 | -0.556 |
| F7 | rs11594693 | 3.72E-08 | 0.239 | 4.02E-06 | 0.257 | 6.91E-03 | 0.187 |
| FetuinA | rs2070633 | 2.88E-44 | -0.629 | 3.01E-31 | -0.670 | 6.78E-14 | -0.547 |
| FetuinA | rs2593813 | 6.74E-44 | -0.698 | 3.92E-31 | -0.749 | 1.21E-13 | -0.591 |
| FGF4 | rs13117858 | 3.12E-10 | 0.410 | 1.21E-07 | 0.452 | 2.81E-04 | 0.371 |
| GROa | rs1263549 | 2.16E-08 | 0.685 | 3.53E-05 | 0.684 | 3.89E-04 | 0.655 |
| GSTa | rs9395826 | 8.19E-11 | -0.396 | 1.10E-07 | -0.414 | 7.66E-05 | -0.353 |
| HCC4 | rs80329614 | 1.16E-27 | -0.958 | 6.15E-21 | -1.069 | 3.53E-09 | -0.818 |
| HP | rs72787038 | 9.69E-14 | 0.468 | 2.03E-09 | 0.537 | 7.54E-07 | 0.383 |
| IL13 | rs7433647 | 1.21E-08 | -0.438 | 3.29E-06 | -0.476 | 1.80E-03 | -0.349 |
| IL16 | rs11556218 | 6.02E-32 | -1.064 | 4.61E-19 | -1.080 | 2.60E-14 | -0.993 |
| IL18 | rs146245376 | 3.06E-08 | -0.559 | 8.09E-05 | -0.498 | 1.12E-04 | -0.653 |
| IL6r | rs12126142 | 1.81E-106 | 0.850 | 4.69E-70 | 0.858 | 4.01E-38 | 0.835 |
| IL6r | rs7526131 | 4.47E-72 | -0.711 | 1.44E-43 | -0.694 | 2.57E-29 | -0.731 |
| IL8 | rs11889675 | 4.60E-08 | 0.364 | 1.67E-05 | 0.378 | 3.67E-04 | 0.353 |
| Leptin | rs2031468 | 1.04E-08 | -0.250 | 5.36E-06 | -0.232 | 7.73E-04 | -0.279 |
| LPa | rs783147 | 1.96E-09 | -0.290 | 8.07E-06 | -0.277 | 3.19E-05 | -0.324 |

| | | | | | | | |
|---|---|---|---|---|---|---|---|
| LPa | rs4646272 | 9.86E-09 | 0.590 | 1.39E-05 | 0.571 | 3.49E-04 | 0.601 |
| MCP2 | rs1133763 | 3.54E-13 | -0.419 | 6.33E-16 | -0.525 | 2.31E-02 | -0.251 |
| MCSF | rs73741236 | 2.08E-08 | -0.841 | 5.95E-03 | -0.542 | 7.62E-07 | -1.170 |
| MCSF | rs111494896 | 2.77E-08 | -0.695 | 1.23E-04 | -0.605 | 1.41E-04 | -0.794 |
| MIP1a | rs2015086 | 2.56E-15 | 0.490 | 2.63E-07 | 0.395 | 4.98E-09 | 0.605 |
| MIP1b | rs145617407 | 2.58E-10 | 0.348 | 3.26E-08 | 0.394 | 2.18E-03 | 0.264 |
| MIP1b | rs4796217 | 1.19E-08 | -0.234 | 1.99E-05 | -0.234 | 1.75E-04 | -0.230 |
| MMP7 | rs9753755 | 8.87E-11 | 0.913 | 7.86E-07 | 1.025 | 2.07E-06 | 0.753 |
| MPIF1 | rs861273 | 6.37E-14 | -0.455 | 4.23E-10 | -0.405 | 1.25E-05 | -0.597 |
| MPIF1 | rs72752381 | 1.37E-08 | 0.767 | 6.58E-03 | 0.375 | 4.12E-08 | 1.587 |
| NrCAM | rs10487851 | 3.01E-10 | 0.242 | 3.89E-09 | 0.272 | 4.50E-03 | 0.194 |
| RAGE | rs2070600 | 1.86E-11 | -0.814 | 1.64E-05 | -0.670 | 6.33E-08 | -1.045 |
| RAGE | rs4953649 | 1.66E-08 | 0.288 | 4.03E-07 | 0.331 | 3.76E-03 | 0.239 |
| SELE | rs507666 | 1.01E-52 | -0.882 | 1.34E-37 | -0.876 | 4.40E-18 | -0.909 |
| Sortilin | rs646776 | 2.20E-09 | 0.331 | 5.63E-05 | 0.275 | 9.63E-07 | 0.464 |
| TECK | rs72927542 | 2.26E-08 | 0.679 | 6.71E-05 | 0.588 | 1.10E-04 | 0.826 |
| TF | rs6762415 | 1.17E-14 | -0.390 | 1.22E-09 | -0.375 | 1.92E-06 | -0.422 |
| TFF3 | rs2444229 | 8.33E-09 | 0.311 | 5.00E-05 | 0.284 | 3.66E-05 | 0.355 |
| THP | rs12934455 | 2.80E-42 | -0.871 | 1.44E-23 | -0.846 | 3.03E-20 | -0.896 |
| THPO | rs2279191 | 3.83E-08 | 0.239 | 2.24E-05 | 0.281 | 2.96E-04 | 0.168 |
| TNC | rs2685421 | 5.27E-11 | 0.358 | 7.21E-07 | 0.330 | 2.20E-05 | 0.408 |
| VCAM1 | rs13027473 | 4.53E-08 | -0.345 | 5.79E-07 | -0.410 | 8.04E-03 | -0.262 |

**Supplementary Table S5. Previously reported SNP-Analyte plasma level associations.**

| Analyte | Chr | SNP | *Gene* | Our GWAS p-value | beta | Reported SNP | Linkage disequilibrium | Our GWAS p-value | Reference |
|---|---|---|---|---|---|---|---|---|---|
| ACE | 17 | rs4343 | *ACE* | 6.66E-44 | 0.493 | rs4343 | $r^2$=1, D'=1 | 6.66E-44 | Kim et al, 2013 |
| AGT | 1 | rs35837081 | *AGT* | 4.45E-34 | 0.890 | rs4762 | $r^2$=0.982, D'=1 | 6.63E-34 | Kim et al, 2013 |
| ApoA4 | 11 | rs1263167 | *APOA4* | 2.64E-54 | -0.919 | rs1263167 | $r^2$=1, D'=1 | 2.64E-54 | Kim et al, 2013 |
| ApoE | 19 | rs769449 | *APOE* | 2.76E-26 | -0.594 | rs445925 | $r^2$=0.028, D'=1 | NA, MAF=0.121 | Surakka et al, 2012 |
| ApoH | 17 | rs2873966 | *APOH* | 3.37E-15 | 0.428 | rs8178841 | $r^2$=0.029, D'=1 | 8.14E-13 | Kim et al, 2013 |
| CA19-9 | 19 | rs485073 | *FUT2* | 2.12E-23 | -0.509 | rs1047781 | $r^2$=0.004, D'=1 | NA, MAF=0.004 | He et al, 2014 |
| CD40 | 20 | rs6032660 | *CD40* | 9.81E-21 | -0.463 | rs1569723 | $r^2$=1, D'=1 | 1.27E-20 | Kim et al, 2013 |
| CD5L | 1 | rs2765501 | *CD5L* | 9.97E-10 | 0.309 | rs2765501 | $r^2$=1, D'=1 | 9.97E-10 | Kim et al, 2013 |
| CEA | 19 | rs570794 | *FUT2* | 2.84E-17 | -0.424 | rs1047781 | $r^2$=0.004, D'=1 | NA, MAF=0.004 | He et al, 2014; Liang et al, 2014 |
| CFHR1 | 1 | rs12144939 | *CFH* | 8.99E-143 | -1.108 | rs6677604; rs7517126 | $r^2$=0.989, D'=1; $r^2$=0.613, D'=0.951 | 3.52E-140; 3.58E-66 | Kim et al, 2013 |
| CystC | 20 | rs13039144 | *CST3* | 1.23E-08 | -0.347 | rs2424590; rs1158167; rs35610040 | r2=0.602, D'=0.778;r2=0.6, D'=1; $r^2$=0.628, D'=1 | 8.05E-7; 9.67E-6; 5.48E-7 | Hwang et al, 2007; Kim et al, 2013; Akerblom et al, 2014 |
| F7 | 13 | rs10665 | *MCF2L* | 1.44E-26 | -0.598 | rs561241 | $r^2$=0.908, D'=1 | 7.37E-26 | Yang et al, 2007 |
| FetuinA | 3 | rs2070633 | *AHSG* | 2.88E-44 | -0.629 | rs2070633 | $r^2$=1, D'=1 | 2.88E-44 | Kim et al, 2013 |
| GSTa | 6 | rs9395826 | *GSTA1* | 8.19E-11 | -0.396 | rs4715326 | $r^2$=0.740, D'=1 | 4.02E-10 | Kim et al, 2013 |
| HCC4 | 17 | rs80329614 | *CCL16* | 1.16E-27 | -0.958 | rs11080369 | $r^2$=0.983, D'=0.991 | 1.17E-25 | Kim et al, 2013 |
| HP | 16 | rs72787038 | *HP* | 9.69E-14 | 0.468 | rs2000999 | $r^2$=0.056, D'=1 | 5.93E-01 | Froguel et al, 2012 |
| IL16 | 15 | rs11556218 | *IL16* | 6.02E-32 | -1.064 | rs4778636 | $r^2$=0.926, D'=0.991 | 2.46E-29 | Kim et al, 2013 |
| IL6r | 1 | rs12126142 | *IL6R* | 1.81E-106 | 0.850 | rs4129267 | $r^2$=1, D'=1 | 1.38E-104 | Melzer et al, 2008; Kim et al, |

| | | | | | | | | | |
|---|---|---|---|---|---|---|---|---|---|
| | | | | | | | | | 2013 |
| LPa | 6 | rs783147 | *PLG* | 1.96E-09 | -0.290 | rs783147 | $r^2$=1, D'=1 | 1.96E-09 | Qi et al, 2012 |
| MCP2 | 17 | rs1133763 | *CCL8* | 3.54E-13 | -0.419 | rs12602195 | $r^2$=0.878, D'=0.985 | 1.03E-11 | Kim et al, 2013 |
| MIP1b | 17 | rs4796217 | *CCL4L2* | 1.19E-08 | -0.234 | rs4796217 | $r^2$=1, D'=1 | 1.19E-08 | Melzer et al, 2008 |
| MPIF1 | 17 | rs861273 | *CCL23* | 6.37E-14 | -0.455 | rs854656 | $r^2$=1, D'=1 | 6.71E-14 | Kim et al, 2013 |
| NrCAM | 7 | rs10487851 | *NRCAM* | 3.01E-10 | 0.242 | rs10487849 | $r^2$=0.952, D'=0.994 | 2.25E-09 | Kim et al, 2013 |
| SELE | 9 | rs507666 | *ABO* | 1.01E-52 | -0.882 | rs579459; rs651007 | $r^2$=0.868, D'=1; $r^2$=0.865, D'=1 | 3.32E-45; 1.57E-45 | Paterson et al, 2009; Qi et al, 2010 |
| TF | 3 | rs6762415 | *TF* | 1.17E-14 | -0.390 | rs3811647 | $r^2$=0.440, D'=1 | 1.53E-06 | Benyamin et al, 2009 |
| THP | 16 | rs12934455 | *UMOD* | 2.80E-42 | -0.871 | rs4293393 | $r^2$=0.847, D'=0.995 | 4.31E-40 | Kim et al, 2013 |

**Supplementary Table S6. See Excel file**

**Supplementary Table S7. Characteristics of Multiple Sclerosis samples**

| | cases | controls |
|---|---|---|
| **Samples** | 20 | 20 |
| **Age (y)** | 44.45±15.51 | 41.84±11.52 |
| **Age range (y)** | 22-72 | 22-68 |
| **Gender (M/F)** | 8/12 | 8/12 |

**Supplementary Table S8. Analyte abbreviations and full analyte names.**

| Abbreviation | Full name |
|---|---|
| A1Micro | Alpha-1-Microglobulin |
| A2Macro | Alpha-2-Macroglobulin |
| AACT | Alpha-1-Antichymotrypsin |
| AAT | Alpha-1-Antitrypsin |
| ACE | Angiotensin-Converting Enzyme |
| Adipo | Adiponectin |
| AFP | Alpha-Fetoprotein |
| AgRP | Agouti-Related Protein |
| AGT | Angiotensinogen |
| ANG2 | Angiopoietin-2 |
| LPa | Apolipoprotein(a) |
| ApoA1 | Apolipoprotein A-I |
| ApoA2 | Apolipoprotein A-II |
| ApoA4 | Apolipoprotein A-IV |
| ApoB | Apolipoprotein B |
| ApoC1 | Apolipoprotein C-I |
| ApoC3 | Apolipoprotein C-III |
| ApoD | Apolipoprotein D |
| ApoE | Apolipoprotein E |
| ApoH | Apolipoprotein H |
| AXL | AXL Receptor Tyrosine Kinase |
| B2M | Beta-2-Microglobulin |
| BDNF | Brain-Derived Neurotrophic Factor |
| BLC | B Lymphocyte Chemoattractant |
| BMP6 | Bone Morphogenetic Protein 6 |
| BTC | Betacellulin |
| C3 | Complement C3 |
| CA199 | Cancer Antigen 19-9 |
| Calcitonin | Calcitonin |
| CD40 | CD 40 antigen |
| CD40L | CD40 Ligand |
| CD5L | CD5 |
| CEA | Carcinoembryonic Antigen |
| CFHR1 | Complement Factor H |
| CgA | Chromogranin-A |
| CKMB | Creatine Kinase-MB |
| CLU | Clusterin |
| CNTF | Ciliary Neurotrophic Factor |
| Cortisol | Cortisol |
| C-peptide | C-peptide |

| | |
|---|---|
| CRP | C-Reactive Protein |
| CystC | Cystatin-C |
| EGF | Epidermal Growth Factor |
| EGFR | Epidermal Growth Factor Receptor |
| ENA78 | Epithelial-Derived Neutrophil-Activating |
| Eotaxin-1 | Eotaxin-1 |
| Eotaxin-3 | Eotaxin-3 |
| F7 | Factor VII |
| FABP | Fatty Acid-Binding Protein- heart |
| FAS | FASLG Receptor |
| FASL | Fas Ligand |
| FRTN | Ferritin |
| FetuinA | Fetuin-A |
| FGF4 | Fibroblast Growth Factor 4 |
| Fibrinogen | Fibrinogen |
| FSH | Follicle-Stimulating Hormone |
| GH | Growth Hormone |
| GROa | Growth-Regulated alpha protein |
| GSTa | Glutathione S-Transferase alpha |
| HBEGF | Heparin-Binding EGF-Like Growth Factor |
| HCC4 | Chemokine CC-4 |
| HGF | Hepatocyte Growth Factor |
| HP | Haptoglobin |
| I309 | T Lymphocyte-Secreted Protein I-309 |
| ICAM1 | Intercellular Adhesion Molecule 1 |
| IgA | Immunoglobulin A |
| IgE | Immunoglobulin E |
| IGFBP2 | Insulin-like Growth Factor-Binding Prote |
| IgM | Immunoglobulin M |
| IL13 | Interleukin-13 |
| IL16 | Interleukin-16 |
| IL18 | Interleukin-18 |
| IL3 | Interleukin-3 |
| IL6r | Interleukin-6 receptor |
| IL8 | Interleukin-8 |
| Insulin | Insulin |
| IP10 | Interferon gamma Induced Protein 10 |
| KIM1 | Kidney Injury Molecule-1 |
| Leptin | Leptin |
| LH | Luteinizing Hormone |
| MB | Myoglobin |
| MCP1 | Monocyte Chemotactic Protein 1 |
| MCP2 | Monocyte Chemotactic Protein 2 |

| | |
|---|---|
| MCP3 | Monocyte Chemotactic Protein 3 |
| MCP4 | Monocyte Chemotactic Protein 4 |
| MCSF | Macrophage Colony-Stimulating Factor 1 |
| MDC | Macrophage-Derived Chemokine |
| MIF | Macrophage Migration Inhibitory Factor |
| MIG | Monokine Induced by Gamma Interferon |
| MIP1a | Macrophage Inflammatory Protein-1 alpha |
| MIP1b | Macrophage Inflammatory Protein-1 beta |
| MIP3a | Macrophage Inflammatory Protein-3 alpha |
| MMP1 | Matrix Metalloproteinase-1 |
| MMP10 | Matrix Metalloproteinase-10 |
| MMP2 | Matrix Metalloproteinase-2 |
| MMP7 | Matrix Metalloproteinase-7 |
| MMP9 | Matrix Metalloproteinase-9 |
| MMP9tot | Matrix Metalloproteinase-9- total |
| MPIF1 | Myeloid Progenitor Inhibitory Factor 1 |
| MPO | Myeloperoxidase |
| NGAL | Neutrophil Gelatinase-Associated Lipocal |
| NrCAM | Neuronal Cell Adhesion Molecule |
| NTproBNP | Brain Natriuretic Peptide |
| Osteopontin | Osteopontin |
| PAI1 | Plasminogen Activator Inhibitor 1 |
| PAP | Prostatic Acid Phosphatase |
| PAPPA | Pregnancy-Associated Plasma Protein A |
| PARC | Pulmonary and Activation-Regulated Chemo |
| PDGFBB | Platelet-Derived Growth Factor BB |
| PLGF | Placenta Growth Factor |
| PPP | Pancreatic Polypeptide |
| PRL | Prolactin |
| Proins | Proinsulin- Intact |
| Proinst | Proinsulin- Total |
| ProteinS | Vitamin K-Dependent Protein S |
| PYY | Peptide YY |
| RAGE | Receptor for advanced glycosylation end |
| RANTES | T-Cell-Specific Protein RANTES |
| Resistin | Resistin |
| SAP | Serum Amyloid P-Component |
| SCF | Stem Cell Factor |
| SELE | E-Selectin |
| SGOT | Serum Glutamic Oxaloacetic Transaminase |
| SHBG | Sex Hormone-Binding Globulin |
| SOD1 | Superoxide Dismutase 1- Soluble |
| Sortilin | Sortilin |

| | |
|---|---|
| TBG | Thyroxine-Binding Globulin |
| TECK | Thymus-Expressed Chemokine |
| Testosterone | Testosterone- Total |
| TF | Serotransferrin |
| TFF3 | Trefoil Factor 3 |
| THP | Tamm-Horsfall Urinary Glycoprotein |
| THPO | Thrombopoietin |
| TIMP1 | Tissue Inhibitor of Metalloproteinases 1 |
| TM | Thrombomodulin |
| TNC | Tenascin-C |
| TNFa | Tumor Necrosis Factor alpha |
| TNFR2 | Tumor Necrosis Factor Receptor-Like 2 |
| TP1 | Thrombospondin-1 |
| TRAILR3 | TNF-Related Apoptosis-Inducing Ligand Re |
| TSH | Thyroid-Stimulating Hormone |
| TTR | Transthyretin |
| VCAM1 | Vascular Cell Adhesion Molecule-1 |
| VEGF | Vascular Endothelial Growth Factor |
| Vitron | Vitronectin |
| vWF | von Willebrand Factor |

## SUPPLEMENTARY METHODS

### Alzheimer's Disease Neuroimaging Initiative (ADNI)

Data used in the preparation of this article were obtained from the Alzheimer's Disease Neuroimaging Initiative (ADNI) database (adni.loni.usc.edu). The ADNI was launched in 2003 by the National Institute on Aging (NIA), the National Institute of Biomedical Imaging and Bioengineering (NIBIB), the Food and Drug Administration (FDA), private pharmaceutical companies and non-profit organizations, as a $60 million, 5-year public-private partnership. The primary goal of ADNI has been to test whether serial magnetic resonance imaging (MRI), positron emission tomography (PET), other biological markers, and clinical and neuropsychological assessment can be combined to measure the progression of mild cognitive impairment (MCI) and early Alzheimer's disease (AD). Determination of sensitive and specific markers of very early AD progression is intended to aid researchers and clinicians to develop new treatments and monitor their effectiveness, as well as lessen the time and cost of clinical trials. The Principal Investigator of this initiative is Michael W. Weiner, MD, VA Medical Center and University of California — San Francisco. ADNI is the result of efforts of many co-investigators from a broad range of academic institutions and private corporations, and subjects have been recruited from over 50 sites across the U.S. and Canada. The initial goal of ADNI was to recruit 800 subjects but ADNI has been followed by ADNI-GO and ADNI-2. To date these three protocols have recruited over 1500 adults, ages 55 to 90, to participate in the research, consisting of cognitively normal older individuals, people with early or late MCI, and people with early AD. The follow up duration of each group is specified in the protocols for ADNI-1, ADNI-2 and ADNI-GO. Subjects originally recruited for ADNI-1 and ADNI-GO had the option to be followed in ADNI-2. Longitudinal imaging, performance on neuropsychological and clinical assessments and biological samples were collected at baseline and at follow-up visits for all of participants. Longitudinal proteomic data was obtained for 566 participants and we used the baseline values of selected analytes. For up-to-date information, see www.adni-info.org.

## SUPPLEMENTARY RESULTS

### *Previously reported findings*

Twenty-eight of the 56 loci associated with specific plasma proteins levels have been previously reported (Table 2, Figure 1, Supplemental Figures S1-S26, and Supplemental Table S5). In this study, a total of 13 SNPs were associated with soluble receptor for advanced glycosylation end products (RAGE/AGER) levels at $p<5\times10^{-8}$ (four at $p<3.42\times10^{-10}$) (Supplemental Table S6). Rs2070600 at chromosome 6q21.3 showed the most significant association with RAGE ($p=1.86\times10^{-11}$) (Supplemental Figure S40). A conditional GWAS adjusting for rs2070600 did not yield any other genome-wide significant association, which suggests that rs2070600 is the dominant contributor to association with RAGE levels. Rs2070600 is located within an exon encoding *AGER*, and is a non-synonymous coding variant in the ligand-binding domain. The polymorphism leads to a substitution of Gly for Ser at residue 82. The association of rs2070600 with systemic RAGE levels was previously reported in subjects with normal glucose metabolism, impaired glucose metabolism, type 2 diabetes mellitus and chronic obstructive pulmonary disease (Supplemental Table S5) [1-4].

We also replicated the associations between rs4343 located on chr17q23.3 and rs495828 located on chr9q34.2 with ACE levels (Supplemental Table S5) [5]. In this study, 94 SNPs were associated with ACE levels at $p<5\times10^{-8}$ (71 at $p<3.42\times10^{-10}$) (Supplemental Table S6). The most significant association was observed with rs4343 ($p=6.66\times10^{-44}$), located in the *ACE* gene (Supplemental Figure S1). Rs4343 is an exonic variant but causes no change in protein sequence (synonymous SNP). Bioinformatic analyses indicated rs4343 is located within a transcription factor (STAT1) binding site, suggesting this variant or a linked variant could influence the expression of *ACE*.

Rs507666 located within an intron of *ABO* blood group gene was strongly associated with plasma SELE levels ($p=1.01\times10^{-52}$) (Supplemental Table S6 and Supplemental Figure S24). Rs507666 is in complete linkage disequilibrium with rs651007, which has been previously reported to be associated with plasma soluble SELE levels ($r^2=0.865$, D'=1) (Supplemental Table S5) [6].

We also replicated the association between rs10665 located on chr13q34 and Factor7 (F7) levels (Supplemental Table S5) [7,8]. Thirty-four SNPs were associated with F7 levels at $p<5\times10^{-8}$ (28 at $p<3.42\times10^{-10}$) (Supplemental Table S6). The most significant association was observed with rs10665 ($p=1.44\times10^{-26}$), within the 3' UTR of *MCF2L*, 7.3 KB from *F7* (Supplemental Figure S12). A conditional GWAS adjusting for the top SNP rs10665 did not yield any other genome-wide significant association in the region surrounding it (± 200 kb). We also identified another SNP on chr10q26.13 (rs11594693), associated with F7 levels ($p=3.72\times10^{-8}$), suggesting that rs2774033 and rs11594693 are the dominant contributors to association with F7 levels (Supplemental Figure S12).

In this study, a total of sixty-six SNPs were associated with Monocyte Chemotactic Protein 2 (MCP2) levels at $p<5\times10^{-8}$ (44 at $p<3.42\times10^{-10}$) (Supplemental Table S6). Rs1133763 located within the *MCP2* gene had the strongest association ($p=1.28\times10^{-13}$) (Supplemental Figure S20). No SNPs reached genome-wide significance in a conditional GWAS adjusted for rs1133763. Rs1133763 is a non-synonymous coding variant causing a substitution from Lys to Gln at the residue 69 of *MCP2*. A previous study has reported the association of another *MCP2* variant (rs12602195, $p=1.03\times10^{-11}$ in our study) with MCP2 levels (Supplemental Table S5) [9]. These two SNPs are in strong linkage disequilibrium with each other ($r^2=0.88$, D'=0.98).

Sixty-eight SNPs were associated with Glutathione S-Transferase alpha (GSTa) levels at $p<5\times10^{-8}$ (18 at $p<3.42\times10^{-10}$) (Supplemental Table S6). The most significant association was found with rs9395826 on chromosome 6p12.2 ($p=8.19\times10^{-11}$), near the *GSTA1* gene (Supplemental Figure S14). A conditional GWAS adjusting for rs9395826 did not yield any other genome-wide significant association. A previous study reported the association of rs4715326 with GSTa levels (Supplemental Table S5) [9]. These two SNPs show high linkage disequilibrium with each other ($r^2=0.74$, D'=1).

Seven SNPs were associated with Alpha-1-antitrypsin (AAT) levels at $p<5\times10^{-8}$ (six at $p<3.42\times10^{-10}$) (Supplemental Table S6). The most significant association was found with rs926144 on chromosome 14q32.1 ($p=4.71\times10^{-12}$), near the *SERPINA1* gene (Supplemental Figure S27). Rs926144, which has a RegulomeDB score of 1f, is an eQTL for *SERPINA1* and affects binding of IKZF1 (Supplemental Table S6). No SNP reached genome-wide significance in a conditional GWAS adjusted for rs926144. A prior study showed association of *SERPINA1* polymorphism (rs4905179) with AAT levels (Supplemental Table S5) [10]. These two SNPs are in near complete linkage disequilibrium (LD) with each other ($r^2=0.94$, D'=0.98).

A total of fifty-one SNPs were associated with Fetuin-A levels at $p<5\times10^{-8}$ (50 at $p<3.42\times10^{-10}$) (Supplemental Table S6). Rs2070633 in the intron of *AHSG* gene, which was previously reported to associate with Fetuin-A levels (Table 2 and Supplemental Table S5) [19], had the most significant p-value ($p=1.35\times10^{-43}$) (Supplemental Figure S13). When running a conditional GWAS, one additional SNP (rs2593813) remained significantly associated with Fetuin-A levels at a genome-wide level of significance ($p=7.27\times10^{-9}$ in the conditional analysis; $p=2.61\times10^{-42}$ in the default model) (Table 3 and Supplemental Figure S13). Bioinformatic analyses indicate rs2593813, located in the intron of the *AHSG* gene, affects binding of POLR2A, CTCF, ETS1 and HEY1 and alters the Olf-1 motif. After conditioning on these two independent SNPs, no additional SNPs were significant for Fetuin-A levels. Although rs2070633 has been previously reported, this was the first time an association between rs2593813 and plasma Fetuin-A levels has been reported.

We were able to replicate the association between rs4796217 located on chr17q12 and plasma levels of Macrophage Inflammatory Protein-1 beta (MIP1b/CCL4) (Supplemental Table S5) [11]. The association between rs4796217 and MIP1b levels was genome-wide significant ($p=1.19\times10^{-8}$) (Supplemental Figure S21). There were no genome-wide significant SNPs after a conditional GWAS adjusted for rs4796217.

A total of 26 SNPs were associated with MPIF1 levels at $p<5\times10^{-8}$ (16 at $p<3.42\times10^{-10}$) (Supplemental Table S6). Rs861273 located on chr17q12, is in complete LD with rs854656 which was previously reported to associate with MPIF1 levels [9], had the most significant p-value ($p=6.62\times10^{-14}$) (Table 2, Supplemental Figure S22, and Supplemental Table S5). A conditional GWAS adjusting for the top SNP rs854656 did not yield any other genome-wide significant association in the surrounding region. We also identified an additional SNP (rs72752381) chr5p13.3, associated with MPIF1 levels ($p=1.37\times10^{-8}$) (Table 2 and Supplemental Figure S22). The role of rs12154780 and rs72752381 on plasma level of MPIF1 has not been previously reported.

We identified thirty-one SNPs associated with plasma Neuronal Cell Adhesion Molecule (NRCAM) levels (two at $p<3.42\times10^{-10}$) (Supplemental Table S6). Rs10487851, within an intron of *NRCAM* gene, had the most significant p-value ($p=3.01\times10^{-10}$) (Supplemental Figure S23). Rs10487851 is located in a transcription factor (P300) binding site, suggesting that rs10487851, or a linked variant, could influence the expression of *NRCAM*. A previous study reported the association of an *NRCAM* variant (rs10487849) with NRCAM levels (Supplemental Table S5) [9]. These two SNPs are in near complete linkage disequilibrium with each other (r2=0.95, D'=0.99). After a conditional GWAS adjusting for rs10487851 there were no genome-wide significant SNPs.

A total of seventy-six SNPs associated with Tamm-Horsfall Urinary Glycoprotein (THP) protein levels (Supplemental Table S6). Rs12934455 in the intron of *UMOD* gene had the most significant p value ($p=2.80\times10^{-42}$) (Table 2 and Supplemental Figure S26). No SNPs reached genome-wide significance in a conditional GWAS adjusted for rs12934455. A variant upstream of *UMOD*, rs4293393, has been previously reported to be associated with THP plasma levels and is in high LD with rs12934455 ($r^2=0.847$) (Supplemental Table S5) [9].

***Novel GWAS hits***

We identified 28 additional novel genome-wide loci associated with plasma proteins levels (Table 2, Figure 1, Supplemental Figures S8, S12, S21-22, S27-S36, and Supplemental Table S5). Fourteen SNPs were associated with Macrophage Inflammatory Protein-1 beta (MIP1b/CCL4) levels at $p<5\times10^{-8}$ (two at $p<3.42\times10^{-10}$) (Supplemental Table S6). The most significant association was found with rs145617407 on chromosome 3p21.31 ($p=2.58\times10^{-10}$), in the intron of *CCR3* gene (Supplemental Figure S21).

We identified 85 SNPs associated with Apolipoprotein H (ApoH) levels (50 at $p<3.42\times10^{-10}$) (Supplemental Table S6). Rs2873966 within the intron of *ApoH* had the most significant p value ($p=1.02\times10^{-15}$) (Figure 1). A previous study reported association of rs10048158 ($p=3.42\times10^{-6}$ in our dataset) located on 17q24.2 with ApoH levels [13]. These two SNPs show low $r^2$ linkage disequilibrium with each other ($r^2=0.27$, D'=0.99). When running conditional GWAS adjusted for the top SNP rs2873966, rs17690171 within 5.3 KB of *ApoH* remained significantly associated with ApoH levels ($p=5.53\times10^{-9}$ in the conditional analysis; $p=1.02\times10^{-15}$ in the default model)

(Table 3 and Figure 1). When we ran a conditional GWAS adjusting for rs2873966 and rs17690171, rs8178828 located within an intron of *APOH* was still significantly associated with ApoH levels ($p=6.64\times10^{-13}$ in the conditional analysis; $p=6.84\times10^{-13}$ in the default model) (Table 3 and Figure 1). After conditioning on rs2873966, rs17690171, and rs8178828 there were no other genome-wide significant SNPs. The associations of rs2873966, rs17690171, and rs8178828 with plasma levels of ApoH have not been previously reported. These results suggest that multiple independent signals in the *APOH* locus modify ApoH protein levels.

A total of sixty-six SNPs were associated with Matrix Metalloproteinase-7 (MMP7) levels at $p<5\times10^{-8}$ (three at $p<3.42\times10^{-10}$) (Supplemental Table S6). Rs9753755 in an intron of *MACROD2* had the most significant p-value ($p=8.87\times10^{-11}$) ( Supplemental Figure S39). When running conditional GWAS there were no additional SNPs within this locus associated with MMP7 plasma levels.

We identified eighty-two SNPs associated with plasma CA19-9 levels (52 at $p<3.42\times10^{-10}$) (Supplemental Table S6). Rs485073 in the 3'UTR of the *fucosyltransferase 2(FUT2)* gene had the strongest association ($p=2.12\times10^{-23}$) ( Supplemental Figure S5). Rs570794 in the same locus was associated with plasma CEA levels ($p = 2.84 \times 10^{-17}$) (Supplemental Figure S8). These two SNPs (rs485073, rs570794) are in near complete linkage disequilibrium with each other ($r^2=0.98$, D'=0.99). Previous studies reported an association of rs1047781 within an exon of the *FUT2* gene with plasma CA19-9 and CEA levels (Supplemental Table S5) [14]. Rs1047781 (MAF=0.10) is not present in our dataset and is located within approximately 1kb of these two variants (rs485073 and rs570794). We also identified another SNP on chr 2q11.2 (rs12468845), associated with CEA levels ($p=3.88\times10^{-8}$) (Supplemental Figure S8). A conditional GWAS adjusting for rs570794 did not yield any other genome-wide significant association in the surrounding region with plasma CEA levels. When running conditional GWAS for rs485073 associated with CA19-9 levels, rs112313064 in *FUT6* remained significant ($p=3.79\times10^{-26}$ in the conditional analysis, $p=7.46\times10^{-23}$ in the default model) (Table 3 and Supplementary Figure S5). Conditioning on rs485073 and rs112313064 revealed a third signal that appeared to be independently associated with CA19-9, rs2306969 located slightly upstream of *FUT3* ($p=2.78\times10^{-9}$ in the conditional analysis, $p=6.11\times10^{-23}$ in the default model)(Table 3 and Supplemental Figure S5).

Our study identified a *DDAH1* variant significantly influencing the plasma B-Lymphocyte Chemoattractant (BLC) level in the present cohorts. The most significant association was found with rs7541151 on chr1p22 ($p=6.44\times10^{-9}$), in the intron of the *DDAH1* (dimethylarginine dimethylaminohydrolase 1) gene (Supplemental Figure S29). Prior studies have shown that *DDAH1* is responsible for the degradation of asymmetric dimethylarginine (ADMA) and *DDAH1* polymorphism (rs1884139, p=0.21 in our dataset) has previously been associated with ADMA levels [15]. These two SNPs (rs7541151, rs1884139) are not in linkage disequilibrium (LD) with each other ($r^2=0.004$, D'=0.47).

We also identified eight SNPs associated with Apolipoprotein(a) (LPa) levels (Supplemental Table S6). Rs783147 in an intron of *PLG*, which was previously reported to associate with LPa

levels, had the most significant p-value ($p=1.96\times10^{-9}$) (Supplemental Figure S19). When running conditional GWAS for SNPs associated with LPa levels, *rs4646272* within *SLC22A1* remained significantly associated with LPa ($p=1.64\times10^{-9}$ in the conditional analysis and $p=9.86\times10^{-9}$ in the default model) (Table 3 and Supplemental Figure S19). A prior study showed the association of the *LPA* variant (rs6919346) with LPa levels ($p=2.41\times10^{-5}$ in our dataset) [16].

Five SNPs were associated with Thrombopoietin (THPO) plasma levels at $p<5\times10^{-8}$ (Supplemental Table S6). Rs2279191, located on chr17q21.31, in the intron of the *DHX8* gene, was significantly associated with THPO levels ($p=3.83\times10^{-8}$) (Supplemental Figure S44).

Twenty-six SNPs were associated with Macrophage Inflammatory Protein-1 alpha (MIP1a/CCL3) levels at $p<5\times10^{-8}$ (21 at $p<3.42\times10^{-10}$) (Supplemental Table S6). The most significant association was found with rs2015086 on chromosome 17q11.2 ($p=2.56\times10^{-15}$), in the promoter region of *CCL18* gene (Supplemental Figure S38). No SNPs reached genome-wide significance in a conditional GWAS adjusted for rs2015086. A previous study showed the association of rs2015086 with higher macrophage expression of CCL18 in human carotid atherosclerotic plaques [17].

A total of 191 SNPs were associated with Interleukin-6 receptor (IL6r) levels at $p<5\times10^{-8}$ (174 at $p<3.42\times10^{-10}$) (Supplemental Table S6). Rs12126142 in the *IL6r* gene had the strongest relationship ($p=1.81\times10^{-106}$) (Supplemental Figure S17). Rs12126142 is in complete linkage disequilibrium with rs2228145, which was previously reported to associate with IL6r levels ($r^2=1$, D'=1) (Supplemental Table S5) [18]. When running a conditional GWAS adjusting for rs12126142, one other SNP in the intron of the *IL6r* gene (rs7526131) remained genome-wide significant ($p=1.43\times10^{-10}$ in the conditional analysis; $p=4.47\times10^{-72}$ in the default model), suggesting that two variants independently modulate IL6r levels (Table 3 and Supplemental Figure S17).
We also identified one SNP (rs3747214) significantly influencing plasma Angiopoietin-2 (ANG2) levels in the present cohorts (Supplemental Table S6). Rs3747214 is located on chromosome 22q13.31 ($p=2.21\times10^{-8}$), within an intron of *PARVG* gene (Supplemental Figure S28). Bioinformatic analyses indicate rs3747214 affects binding of SP1 and IRF1 and is situated in the Pax5 binding motif.

Eight SNPs were associated with C-Reactive Protein (CRP) levels at $p<5\times10^{-8}$ (three at $p<3.42\times10^{-10}$) (Supplemental Table S6). Rs12972156 within an intron of the *PVRL2* gene had the most significant p value ($p=9.93\times10^{-11}$) (Supplemental Figure S10). No SNPs reached genome-wide significance in a conditional GWAS adjusted for rs12972156. Previous studies have reported association of a *PVRL2* variant (rs6857) with Alzheimer's disease biomarkers and age-related macular degeneration [20,21]. These two SNPs are in strong LD with each other ($r^2=0.84$, D'=1).

A total of forty-two SNPs were associated with Fibroblast Growth Factor 4 (FGF4) levels at $p<5\times10^{-8}$ (two at $p<3.42\times10^{-10}$) (Supplemental Table S6). The most significant association was found with rs13117858 on chromosome 4q34.1 ($p=4.54\times10^{-10}$), in an intron of *GALNTL6* gene

(Supplemental Figure S31). No SNPs reached genome-wide significance in a conditional GWAS adjusted for rs13117858, which suggests that rs1133763 is the dominant contributor to association with FGF4 levels.

We identified two SNPs associated with Growth-Regulated alpha protein (GROa) levels (Supplemental Table S6). Rs1263549, in the intron of the *PTPRN2* gene, had the most significant p value ($p=2.16\times10^{-8}$) (Table 2 and Supplemental Figure S32). No SNPs reached genome-wide significance in a conditional GWAS adjusted for rs1263549. Previous studies reported association of *PTPRN2* variants (rs10274279, rs4909189, rs6459804, rs7786808) with myopia, response to amphetamines, bipolar disorder and schizophrenia, obesity-related traits respectively ($p>0.05$ in our dataset) [22-25]. These SNPs were not in LD with rs1263549 ($r^2=0.007$, $r^2=0.0005$, $r^2=0.02$, $r^2=0.001$ respectively).

We identified ten SNPs associated with Epithelial-Derived Neutrophil-Activating (ENA78; CXCL5) protein levels (Supplemental Table S6). Rs409336 near the *CXCL5* gene had the most significant p value ($p=1.11\times10^{-8}$) (Table 2 and Supplemental Figure S30). No SNPs reached genome-wide significance in a conditional GWAS adjusted for rs409336.

Thirty-two SNPs were associated with plasma haptoglobin (HP) protein levels at $p<5\times10^{-8}$ (seven at $p<3.42\times10^{-10}$) (Supplemental Table S6). The most significant association was found with rs72787038 on chr16q22, near *DHODH* gene ($p=9.69\times10^{-14}$) (Table 2 and Supplemental Figure S16). A conditional GWAS adjusting for rs72787038 did not yield any other genome-wide significant association in the region surrounding it. A previous study reported the association of a *haptoglobin* variant (rs2000999) with HP levels ($p>0.05$ in our dataset) [26]. These two SNPs (rs72787038, rs2000999) show low $r^2$ linkage disequilibrium with each other ($r^2=0.05$, D'=1).

We identified one SNP (rs7433647) significantly influencing the plasma Interleukin-13 (IL-13) level in the present cohorts ($p=1.21\times10^{-8}$) (Table 2, Supplemental Figure S34, and Supplemental S7). Rs7433647 is located on chromosome 3p24.3, near the *UBE2E2* gene.

One SNP (rs146245376) was strongly associated with plasma Interleukin-18 (IL18) levels ($p=3.06\times10^{-8}$) (Table 2, Supplemental Figure S35, and Supplemental S7). Rs146245376 is located near the *MIR3169* gene.

One SNP was strongly associated with Interleukin-8 (IL8) levels, rs11889675 near the *TAF1B* gene ($p=4.60\times10^{-8}$) (Table 2, Supplemental Figure S35, and Supplemental S7). Bioinformatic analyses indicated rs11889675 affects binding of POLR2A and is situated in the NF-E2 binding motif. No other SNP reached genome-wide significance in a conditional GWAS adjusted for rs11889675.

One SNP (rs2031468) was strongly associated with plasma Leptin levels ($p=1.04\times10^{-8}$) (Table 2, Supplemental Figure S35, and Supplemental S7). Rs2031468 is located on chromosome 10q26.3, near the *GLRX3* gene.

Six SNPs were associated with Thymus-Expressed Chemokine (TECK) levels at $p<5\times10^{-8}$ (Supplemental Table S6). The most significant association was found with rs72927542 near the *ST8SIA3* gene ($p=2.26\times10^{-8}$) (Table 2 and Supplemental Figure S42). A conditional GWAS adjusting for rs72927542 did not yield any other genome-wide significant association.

We identified 31 SNPs associated with Tenascin-C (TNC) levels (seven at $p<3.42\times10^{-10}$) and rs2685419 had the most significant p-value ($p=5.27\times10^{-11}$) (Table 2, Supplemental Figure S45, and Supplemental S7). Rs2685419 is located on 8p21.1, in the intron of the *SCARA5* gene. A conditional GWAS adjusting for the top SNP rs2685419 did not yield any other genome-wide significant association in the surrounding region. A previous study showed the association of a *SCARA5* variant (rs11774576) with adverse response to chemotherapy (neutropenia/leucopenia) ($p>0.05$ in our dataset) [27]. These two SNPs were not in LD with each other ($r^2=0.003$, D'=0.09).

Five SNPs were associated with Sortilin protein levels at $p<5\times10^{-8}$ (Supplemental Table S6). The most significant association was found with rs646776 on chromosome 1p13.3 ($p=2.20\times10^{-9}$), downstream of the *CELSR2* gene (Table 2 and Supplemental Figure S41). Rs646776, which has a RegulomeDB score of 1f, is an eQTL for *CELSR2* and is predicted to affect binding of five different proteins (CTCF, HEY1, REST, POLR2A and ZBTB7A). No other SNP reached genome-wide significance in a conditional GWAS adjusted for rs646776. Previous studies showed the association of rs646776 with plasma Progranulin levels and Lipid (total cholesterol and LDL cholesterol) levels [28,29].

We identified one SNP (rs6762415) significantly influencing transferrin (TF) levels in the present cohorts ($p=1.17\times10^{-14}$) (Table 2, Supplemental Figure S25, and Supplemental Table S6). Rs6762415 was located on chromosome 3q22.1, within an intron of the *TF* gene. A previous study showed the association of *TF* variants (rs1799852, rs3811647, rs1830084, rs2280673) and *hemochromatosis* variant (rs1800562) with serum TF levels ($p=0.01$, $p=1.53\times10^{-6}$, $p=3.01\times10^{-5}$, $p=0.006$, $p=1.49\times10^{-6}$ respectively in our dataset) [30]. These SNPs were all in low LD with rs6762415 from our study ($r^2=0.11$, $r^2=0.44$, $r^2=0.35$, $r^2=0.42$, $r^2=0.0007$ respectively).

We also identified one SNP (rs13027473) associated with plasma vascular cell adhesion molecule-1 (VCAM-1) protein levels (Supplemental Table S6). The most significant association was found with rs13027473 near the *RAMP1* gene ($p=4.53\times10^{-8}$) (Table 2 and Supplemental Figure S46). There were no significant SNPs after conditioning on rs13027473.

We identified two SNPs (rs73741236, rs111494896) associated with Macrophage Colony-Stimulating Factor 1 (MCSF) protein levels (Supplemental Table S6). The most significant association was found with rs73741236 on chr5p15.2 ($p=2.08\times10^{-8}$), near the *CTNND2* gene (Table 2 and Supplemental Figure S37). Rs111494896 is located on chr8p21.3, near the *GFRA2* gene ($p=2.77\times10^{-8}$) (Table 2 and Supplemental Figure S37).

We also identified three SNPs associated with plasma Trefoil Factor 3 (TFF3) protein levels

(Supplemental Table S6). The most significant association was found with rs2444229 near the *MIR4790 g*ene ($p=8.33\times10^{-9}$) (Table 2 and Supplemental Figure S43). SNPs became non-significant after conditioning on rs2444229.

**Impact on complex traits**

Common variants in the *ApoH* gene have previously been implicated in blood pressure responses to high sodium and potassium intervention [31]. We also identified three independent *ApoH* variants associated with ApoH levels. Potential pleiotropy of the *APOH* locus may indicate common genetic mechanisms between APOH levels and blood pressure responses to high sodium and potassium intervention.

CRP is an acute phase reactant that can be used as a general screening aid for inflammatory diseases, infections, and neoplastic diseases [32]. Elevated serum CRP level is a marker for increased systemic inflammation and is associated with both macrovascular (i.e stroke and coronary heart disease) and microvascular (i.e diabetic nephropathy and chronic kidney disease) diseases [33-36]. Previous studies investigated the role of polymorphisms near the *CRP* gene in relation to CRP levels [37-41]. We found rs12972156 within the *PVRL2* gene to be associated with plasma CRP levels. Variants in *PVRL2-APOE* have previously been associated with Alzheimer's disease, age-related macular degeneration and HDL cholesterol in various GWAS [20,21,42]. Interestingly, the top SNP from the present study is in high LD ($r^2=0.84$, D'=1) with a SNP (rs6857) previously associated with Alzheimer's disease biomarkers, suggesting that CRP might be involved in pathophysiology of Alzheimer's disease. Further experimental studies are needed to determine the mechanistic relationship between CRP and Alzheimer's disease.

B-Lymphocyte Chemoattractant (BLC; CXCL13) is a member of the CXC subtype of the chemokine superfamily [49]. It is critical for secondary lymphoid tissue development and distribution of lymphocytes within microenvironments [50]. Elevated serum CXCL13 levels were found in patients with chronic hepatitis C virus infection and ANCA-associated vasculitis [51,52]. In our study, rs7541151 on the *DDAH1* gene was associated with the plasma BLC level. DDAH1 is responsible for the degradation of ADMA into citrulline and dimethylamine and previous studies showed the association of *DDAH1* variants with multiple sclerosis and ADMA levels [15,53]. The pleiotropic *DDAH1* loci were associated with several plasma biomarkers and multiple sclerosis, supporting the hypothesis that the pathobiology of multiple sclerosis is heterogeneous and has numerous underlying mechanisms.

Epithelial-Derived Neutrophil-Activating (ENA78; CXCL5) is a member of the CXC chemokines and is a chemoattractant that activates neutrophils. Its expression is elevated in the inflamed tissues of patients with rheumatoid arthritis, ulcerative colitis and Crohn's disease [54,55]. Several studies showed the association of *CXCL5* variants with inflammatory bowel disease and metabolite levels [56,57]. In our study, rs409336 near the *CXCL5* gene showed the strongest effect on the plasma CXCL5 level. High CXCL5 levels have been reported in patients with inflammatory bowel disease [54,55]. Because of similarity in genetic influences on CXCL5 levels and inflammatory bowel disease, it is possible that these traits share a common

pathophysiological pathway and our findings support further investigation of the involvement of CXCL5 in etiology of inflammatory bowel disease.

Growth-Regulated alpha protein (GROa; CXCL1) is a 107 amino acid protein that plays a major role in inflammation, angiogenesis, tumorigenesis, and wound healing [58-60]. We found one SNP rs1263549 in the *protein tyrosine phosphatase, receptor type, N polypeptide 2* (*PTPRN2*) gene associated with plasma GROa levels. *PTPRN2* gene encodes a 1015-amino acid polypeptide with a single transmembrane and one putative tyrosine phosphatase catalytic domain [61]. Previous studies reported that *PTPRN2* variants have been implicated in several human phenotypes, including myopia, response to amphetamines and obesity-related traits [22-24]. Variants in this gene have also previously been associated with bipolar disorder, schizophrenia and chronic kidney disease [25,62]. The LD between these SNPs from the prior studies and rs1263549 from our study is very low, indicating that these signals are independent. *PTPRN2* locus contains multiple variants affecting risk for several complex human traits and diseases, suggesting that they have pleiotropic effects.

Sortilin belongs to the Vps10p domain receptor family and functions as a sorting receptor in the Golgi compartment. It is involved in a number of important biological processes such as the formation of glucose transport 4 (GLUT-4) storage vesicles in response to insulin during adipocyte differentiation [63]. In the brain it is part of a signaling complex that regulates cell survival [64]. Our study identified one SNP (rs646776), which was previously associated with plasma progranulin and lipid levels [28,29], significantly influencing the plasma sortilin level. Rs646776 is located downstream of the *CELSR2* gene. Bioinformatic analyses indicate that *sortilin* and *CELSR2* are in the same linkage disequilibrium block and that rs646776 is an eQTL for *CELSR2* and is predicted to affect transcription factors binding sites. This suggests that the *CELSR2* variant could regulate the expression of the *Sortilin* gene.

Haptoglobin (HP) is an acute phase inflammatory marker and its main function is to bind hemoglobin released from erythrocytes to aid in its elimination [65]. HP levels have been repeatedly associated with a variety of inflammation-linked infectious and non-infectious diseases, including malaria, tuberculosis, human immunodeficiency virus, hepatitis C, diabetes, carotid atherosclerosis, and acute myocardial infarction [66-75]. Previous studies reported the association of *haptoglobin* variant (rs2000999) with HP levels [26]. Our study identified rs72787038, 16q22.2; significantly influencing the plasma HP levels in the present cohorts. This was not in LD with rs2000999 ($r^2$=0.05) and none of these associations have been previously reported.

Interleukin (IL)-13 is an immunoregulatory cytokine secreted by numerous immune cells [76]. IL-13 levels have been described as elevated in patients with septic shock, bronchial asthma and systemic inflammatory response syndrome [77-79]. We found that a variant (rs7433647) near *UBE2E2* was associated with plasma IL-13 levels. *UBE2E2*, located at 3p24.2, encodes the ubiquitin-conjugating enzyme E2E2, which is reported to be expressed in human pancreas, liver, muscle and adipose tissue, as well as in a cultured insulin-secreting cell line [80]. Previous studies identified the association of *UBE2E2* variants with type 2 diabetes and atypical

psychosis [81,82]. The association of *UBE2E2* variants with IL-13 levels indicates potential novel pathophysiology for type 2 diabetes and atypical psychosis.

Leptin is a protein hormone produced predominantly in the adipocytes which plays an important role in energy homeostasis, obesity and the regulation of body weight [83]. Leptin levels have been associated with obesity, blood pressure elevation, insulin resistance, metabolic syndromes, type 2 diabetes mellitus, coronary heart disease and stroke [84-86]. Our study identified one SNP (rs2031468) near the *GLRX3* gene related to plasma leptin level in the dataset. Previous studies reported the association of *GLRX3* variant with HIV-1 susceptibility [87]. The significant effect of rs2031468 on the plasma levels of leptin has not been previously reported.

Matrix Metalloproteinase-7 (MMP-7) is a secreted matrix metalloproteinase that is overexpressed in many cancers and contributes to angiogenesis by breaking down basement membranes [88]. Elevated serum MMP-7 levels were found in patients with systemic sclerosis, lung fibrosis, sarcoidosis and a variety of cancers [89-92]. We found that a variant (rs9753755) in *MACROD2* was associated with plasma MMP-7 levels. The function of MACROD2 is largely unknown. The protein contains a MACRO domain which is a high-affinity ADP-ribose-binding domain that is important in multiple biological processes. Previous studies reported that *MACROD2* locus also harbors variants that influence several human traits, including eating disorders, brain connectivity, antiphospholipid antibodies, hypertension, obesity-related traits, non-alcoholic fatty liver disease histology and autismin [22,93-98] and these variants were not in LD with the top SNP in our study.
Vascular Cell Adhesion Molecule-1 (VCAM-1) supports the adhesion of lymphocytes, monocytes, natural killer cells, eosinophils, and basophils through its interaction with leukocyte very late antigen-4 (VLA-4) [99]. Increased levels of sVCAM-1 can be detected in many inflammatory diseases [100-106]. We identified one SNP (rs13027473) near the *RAMP1* gene, associated with VCAM-1 levels. Previous studies investigated the role of polymorphism (rs10185142) near *RAMP1* gene in relation to inherited obesity-related traits [22]. These two SNPs are not in LD ($r^2$=0.003) and the role of rs13027473 on plasma level of VCAM-1 has not been previously reported.

Macrophage Inflammatory Protein-1 beta (MIP1b) is a monokine with inflammatory and chemokinetic properties. It is involved in the cell activation of rodent granulocytes (neutrophils, eosinophils, and basophils) and appears to be involved in acute neutrophilic inflammation [107,108]. Our study identified two independent SNPs (rs145617407, *CCR3*; rs4796217,*CCL4L2*), significantly influencing the plasma MIP1b level in our dataset. A previous study reported the association between rs4796217 and MIP1b levels [11], but rs145617407 has not previously been reported to be associated with MIP1b. Previous studies reported association of *CCR3* variants with MCP1 levels, obesity-related traits, monocyte chemoattractant protein-1levels and celiac disease [12,22,109,110].

Iron is essential for biochemical functions such as oxygen transport and oxidative

phosphorylation. Excessive iron can cause iron-overload-related disease, whereas iron deficiency can lead to anemia. Iron status can be assessed by measuring the levels of serum iron, serum TF, TF saturation with iron, and serum ferritin [30,111-113]. We identified one *TF* variant (rs6762415) associated with plasma TF levels. In previous GWAS, variants in *TF* and *hemochromatosis* genes have been associated with serum TF levels and explained ~40% of genetic variation in serum TF [30]. However, the top SNP from our study is in mild LD with the SNPs from the previous study, suggesting that the underlying signal in our study is independent.

**REFERENCES**


1. Hancock DB, Artigas MS, Gharib SA, Henry A, Manichaikul A, et al. (2012) Genome-wide joint meta-analysis of SNP and SNP-by-smoking interaction identifies novel loci for pulmonary function. PLoS Genet 8: e1003098.
2. Hancock DB, Eijgelsheim M, Wilk JB, Gharib SA, Loehr LR, et al. (2010) Meta-analyses of genome-wide association studies identify multiple loci associated with pulmonary function. Nat Genet 42: 45-52.
3. Repapi E, Sayers I, Wain LV, Burton PR, Johnson T, et al. (2010) Genome-wide association study identifies five loci associated with lung function. Nat Genet 42: 36-44.
4. Kang P, Tian C, Jia C (2012) Association of RAGE gene polymorphisms with type 2 diabetes mellitus, diabetic retinopathy and diabetic nephropathy. Gene 500: 1-9.
5. Chung CM, Wang RY, Chen JW, Fann CS, Leu HB, et al. (2010) A genome-wide association study identifies new loci for ACE activity: potential implications for response to ACE inhibitor. Pharmacogenomics J 10: 537-544.
6. Qi L, Cornelis MC, Kraft P, Jensen M, van Dam RM, et al. (2010) Genetic variants in ABO blood group region, plasma soluble E-selectin levels and risk of type 2 diabetes. Hum Mol Genet 19: 1856-1862.
7. Tang W, Schwienbacher C, Lopez LM, Ben-Shlomo Y, Oudot-Mellakh T, et al. (2012) Genetic associations for activated partial thromboplastin time and prothrombin time, their gene expression profiles, and risk of coronary artery disease. Am J Hum Genet 91: 152-162.
8. Yang Q, Kathiresan S, Lin JP, Tofler GH, O'Donnell CJ (2007) Genome-wide association and linkage analyses of hemostatic factors and hematological phenotypes in the Framingham Heart Study. BMC Med Genet 8 Suppl 1: S12.
9. Kim S, Swaminathan S, Inlow M, Risacher SL, Nho K, et al. (2013) Influence of genetic variation on plasma protein levels in older adults using a multi-analyte panel. PLoS One 8: e70269.
10. Thun GA, Imboden M, Ferrarotti I, Kumar A, Obeidat M, et al. (2013) Causal and synthetic associations of variants in the SERPINA gene cluster with alpha1-antitrypsin serum levels. PLoS Genet 9: e1003585.
11. Melzer D, Perry JR, Hernandez D, Corsi AM, Stevens K, Rafferty I, Lauretani F, Murray A, Gibbs JR, Paolissao G, et al. (2008) A genome-wide association study identified protein quantitative trait loci (pQTLs). PLoS Genet. 4,e1000072.
12. Voruganti VS, Laston S, Haack K, Mehta NR, Smith CW, et al. (2012) Genome-wide association replicates the association of Duffy antigen receptor for chemokines (DARC) polymorphisms with serum monocyte chemoattractant protein-1 (MCP-1) levels in Hispanic children. Cytokine 60: 634-638.
13. Athanasiadis G, Sabater-Lleal M, Buil A, Souto JC, Borrell M, et al. (2013) Genetic determinants of plasma beta(2)-glycoprotein I levels: a genome-wide association study in extended pedigrees from Spain. J Thromb Haemost 11: 521-528.
14. He M, Wu C, Xu J, Guo H, Yang H, et al. (2014) A genome wide association study of genetic loci that influence tumour biomarkers cancer antigen 19-9, carcinoembryonic antigen and alpha fetoprotein and their associations with cancer risk. Gut 63: 143-151.

15. Seppala I, Kleber ME, Lyytikainen LP, Hernesniemi JA, Makela KM, et al. (2014) Genome-wide association study on dimethylarginines reveals novel AGXT2 variants associated with heart rate variability but not with overall mortality. Eur Heart J 35: 524-531.
16. Ober C, Nord AS, Thompson EE, Pan L, Tan Z, et al. (2009) Genome-wide association study of plasma lipoprotein(a) levels identifies multiple genes on chromosome 6q. J Lipid Res 50: 798-806.
17. Hagg DA, Olson FJ, Kjelldahl J, Jernas M, Thelle DS, et al. (2009) Expression of chemokine (C-C motif) ligand 18 in human macrophages and atherosclerotic plaques. Atherosclerosis 204: e15-20.
18. van Dongen J, Jansen R, Smit D, Hottenga JJ, Mbarek H, et al. (2014) The contribution of the functional IL6R polymorphism rs2228145, eQTLs and other genome-wide SNPs to the heritability of plasma sIL-6R levels. Behav Genet 44: 368-382.
19. Fisher E, Stefan N, Saar K, Drogan D, Schulze MB, et al. (2009) Association of AHSG gene polymorphisms with fetuin-A plasma levels and cardiovascular diseases in the EPIC-Potsdam study. Circ Cardiovasc Genet 2: 607-613.
20. Ramanan VK, Risacher SL, Nho K, Kim S, Swaminathan S, et al. (2014) APOE and BCHE as modulators of cerebral amyloid deposition: a florbetapir PET genome-wide association study. Mol Psychiatry 19: 351-357.
21. Holliday EG, Smith AV, Cornes BK, Buitendijk GH, Jensen RA, et al. (2013) Insights into the genetic architecture of early stage age-related macular degeneration: a genome-wide association study meta-analysis. PLoS One 8: e53830.
22. Comuzzie AG, Cole SA, Laston SL, Voruganti VS, Haack K, et al. (2012) Novel genetic loci identified for the pathophysiology of childhood obesity in the Hispanic population. PLoS One 7: e51954.
23. Meng W, Butterworth J, Bradley DT, Hughes AE, Soler V, et al. (2012) A genome-wide association study provides evidence for association of chromosome 8p23 (MYP10) and 10q21.1 (MYP15) with high myopia in the French Population. Invest Ophthalmol Vis Sci 53: 7983-7988.
24. Hart AB, Engelhardt BE, Wardle MC, Sokoloff G, Stephens M, et al. (2012) Genome-wide association study of d-amphetamine response in healthy volunteers identifies putative associations, including cadherin 13 (CDH13). PLoS One 7: e42646.
25. Curtis D, Vine AE, McQuillin A, Bass NJ, Pereira A, et al. (2011) Case-case genome-wide association analysis shows markers differentially associated with schizophrenia and bipolar disorder and implicates calcium channel genes. Psychiatr Genet 21: 1-4.
26. Froguel P, Ndiaye NC, Bonnefond A, Bouatia-Naji N, Dechaume A, et al. (2012) A genome-wide association study identifies rs2000999 as a strong genetic determinant of circulating haptoglobin levels. PLoS One 7: e32327.
27. Low SK, Chung S, Takahashi A, Zembutsu H, Mushiroda T, et al. (2013) Genome-wide association study of chemotherapeutic agent-induced severe neutropenia/leucopenia for patients in Biobank Japan. Cancer Sci 104: 1074-1082.
28. Carrasquillo MM, Nicholson AM, Finch N, Gibbs JR, Baker M, et al. (2010) Genome-wide screen identifies rs646776 near sortilin as a regulator of progranulin levels in human plasma. Am J Hum Genet 87: 890-897.

29. Chasman DI, Pare G, Mora S, Hopewell JC, Peloso G, et al. (2009) Forty-three loci associated with plasma lipoprotein size, concentration, and cholesterol content in genome-wide analysis. PLoS Genet 5: e1000730.
30. Benyamin B, McRae AF, Zhu G, Gordon S, Henders AK, et al. (2009) Variants in TF and HFE explain approximately 40% of genetic variation in serum-transferrin levels. Am J Hum Genet 84: 60-65.
31. He J, Kelly TN, Zhao Q, Li H, Huang J, et al. (2013) Genome-wide association study identifies 8 novel loci associated with blood pressure responses to interventions in Han Chinese. Circ Cardiovasc Genet 6: 598-607.
32. Oh J, Teoh H, Leiter LA (2011) Should C-reactive protein be a target of therapy? Diabetes Care 34 Suppl 2: S155-160.
33. Buckley DI, Fu R, Freeman M, Rogers K, Helfand M (2009) C-reactive protein as a risk factor for coronary heart disease: a systematic review and meta-analyses for the U.S. Preventive Services Task Force. Ann Intern Med 151: 483-495.
34. Makita S, Nakamura M, Satoh K, Tanaka F, Onoda T, et al. (2009) Serum C-reactive protein levels can be used to predict future ischemic stroke and mortality in Japanese men from the general population. Atherosclerosis 204: 234-238.
35. Saraheimo M, Teppo AM, Forsblom C, Fagerudd J, Groop PH (2003) Diabetic nephropathy is associated with low-grade inflammation in Type 1 diabetic patients. Diabetologia 46: 1402-1407.
36. Fox ER, Benjamin EJ, Sarpong DF, Nagarajarao H, Taylor JK, et al. (2010) The relation of C--reactive protein to chronic kidney disease in African Americans: the Jackson Heart Study. BMC Nephrol 11: 1.
37. Dorajoo R, Li R, Ikram MK, Liu J, Froguel P, et al. (2013) Are C-reactive protein associated genetic variants associated with serum levels and retinal markers of microvascular pathology in Asian populations from Singapore? PLoS One 8: e67650.
38. Reiner AP, Beleza S, Franceschini N, Auer PL, Robinson JG, et al. (2012) Genome-wide association and population genetic analysis of C-reactive protein in African American and Hispanic American women. Am J Hum Genet 91: 502-512.
39. Dehghan A, Dupuis J, Barbalic M, Bis JC, Eiriksdottir G, et al. (2011) Meta-analysis of genome-wide association studies in >80 000 subjects identifies multiple loci for C-reactive protein levels. Circulation 123: 731-738.
40. Okada Y, Takahashi A, Ohmiya H, Kumasaka N, Kamatani Y, et al. (2011) Genome-wide association study for C-reactive protein levels identified pleiotropic associations in the IL6 locus. Hum Mol Genet 20: 1224-1231.
41. Elliott P, Chambers JC, Zhang W, Clarke R, Hopewell JC, et al. (2009) Genetic Loci associated with C-reactive protein levels and risk of coronary heart disease. Jama 302: 37-48.
42. Kim YJ, Go MJ, Hu C, Hong CB, Kim YK, et al. (2011) Large-scale genome-wide association studies in East Asians identify new genetic loci influencing metabolic traits. Nat Genet 43: 990-995.
43. Morillo-Bernal J, Fernandez-Santos JM, Utrilla JC, de Miguel M, Garcia-Marin R, et al. (2009) Functional expression of the thyrotropin receptor in C cells: new insights into their involvement in the hypothalamic-pituitary-thyroid axis. J Anat 215: 150-158.

44. Foa P, Ortolani S, Pogliani EM, Iurlo A, Gualdoni A, et al. (1990) Immunoreactive calcitonin: a tumor marker for myelogenous leukemias. Int J Biol Markers 5: 27-30.
45. Ihalainen J, Juvonen E, Savolainen ER, Ruutu T, Palotie A (1994) Calcitonin gene methylation in chronic myeloproliferative disorders. Leukemia 8: 230-235.
46. Teslovich TM, Musunuru K, Smith AV, Edmondson AC, Stylianou IM, et al. (2010) Biological, clinical and population relevance of 95 loci for blood lipids. Nature 466: 707-713.
47. Kathiresan S, Willer CJ, Peloso GM, Demissie S, Musunuru K, et al. (2009) Common variants at 30 loci contribute to polygenic dyslipidemia. Nat Genet 41: 56-65.
48. Liu C, Batliwalla F, Li W, Lee A, Roubenoff R, et al. (2008) Genome-wide association scan identifies candidate polymorphisms associated with differential response to anti-TNF treatment in rheumatoid arthritis. Mol Med 14: 575-581.
49. Gunn MD, Ngo VN, Ansel KM, Ekland EH, Cyster JG, et al. (1998) A B-cell-homing chemokine made in lymphoid follicles activates Burkitt's lymphoma receptor-1. Nature 391: 799-803.
50. Legler DF, Loetscher M, Roos RS, Clark-Lewis I, Baggiolini M, et al. (1998) B cell-attracting chemokine 1, a human CXC chemokine expressed in lymphoid tissues, selectively attracts B lymphocytes via BLR1/CXCR5. J Exp Med 187: 655-660.
51. Sansonno D, Tucci FA, Troiani L, Lauletta G, Montrone M, et al. (2008) Increased serum levels of the chemokine CXCL13 and up-regulation of its gene expression are distinctive features of HCV-related cryoglobulinemia and correlate with active cutaneous vasculitis. Blood 112: 1620-1627.
52. Monach PA (2014) Biomarkers in vasculitis. Curr Opin Rheumatol 26: 24-30.
53. Sawcer S, Hellenthal G, Pirinen M, Spencer CC, Patsopoulos NA, et al. (2011) Genetic risk and a primary role for cell-mediated immune mechanisms in multiple sclerosis. Nature 476: 214-219.
54. Walz A, Schmutz P, Mueller C, Schnyder-Candrian S (1997) Regulation and function of the CXC chemokine ENA-78 in monocytes and its role in disease. J Leukoc Biol 62: 604-611.
55. Z'Graggen K, Walz A, Mazzucchelli L, Strieter RM, Mueller C (1997) The C-X-C chemokine ENA-78 is preferentially expressed in intestinal epithelium in inflammatory bowel disease. Gastroenterology 113: 808-816.
56. Jostins L, Ripke S, Weersma RK, Duerr RH, McGovern DP, et al. (2012) Host-microbe interactions have shaped the genetic architecture of inflammatory bowel disease. Nature 491: 119-124.
57. Inouye M, Ripatti S, Kettunen J, Lyytikainen LP, Oksala N, et al. (2012) Novel Loci for metabolic networks and multi-tissue expression studies reveal genes for atherosclerosis. PLoS Genet 8: e1002907.
58. Devalaraja RM, Nanney LB, Du J, Qian Q, Yu Y, et al. (2000) Delayed wound healing in CXCR2 knockout mice. J Invest Dermatol 115: 234-244.
59. Haghnegahdar H, Du J, Wang D, Strieter RM, Burdick MD, et al. (2000) The tumorigenic and angiogenic effects of MGSA/GRO proteins in melanoma. J Leukoc Biol 67: 53-62.
60. Owen JD, Strieter R, Burdick M, Haghnegahdar H, Nanney L, et al. (1997) Enhanced tumor-forming capacity for immortalized melanocytes expressing melanoma growth stimulatory activity/growth-regulated cytokine beta and gamma proteins. Int J Cancer 73: 94-103.

61. Wasmeier C, Hutton JC (1996) Molecular cloning of phogrin, a protein-tyrosine phosphatase homologue localized to insulin secretory granule membranes. J Biol Chem 271: 18161-18170.
62. Yoshida T, Kato K, Yokoi K, Oguri M, Watanabe S, et al. (2010) Association of genetic variants with chronic kidney disease in Japanese individuals with or without hypertension or diabetes mellitus. Exp Ther Med 1: 137-145.
63. Shi J, Kandror KV (2005) Sortilin is essential and sufficient for the formation of Glut4 storage vesicles in 3T3-L1 adipocytes. Dev Cell 9: 99-108.
64. Vaegter CB, Jansen P, Fjorback AW, Glerup S, Skeldal S, et al. (2011) Sortilin associates with Trk receptors to enhance anterograde transport and neurotrophin signaling. Nat Neurosci 14: 54-61.
65. Gabay C, Kushner I (1999) Acute-phase proteins and other systemic responses to inflammation. N Engl J Med 340: 448-454.
66. Kasvosve I, Speeckaert MM, Speeckaert R, Masukume G, Delanghe JR (2010) Haptoglobin polymorphism and infection. Adv Clin Chem 50: 23-46.
67. Langlois MR, Delanghe JR (1996) Biological and clinical significance of haptoglobin polymorphism in humans. Clin Chem 42: 1589-1600.
68. Asleh R, Marsh S, Shilkrut M, Binah O, Guetta J, et al. (2003) Genetically determined heterogeneity in hemoglobin scavenging and susceptibility to diabetic cardiovascular disease. Circ Res 92: 1193-1200.
69. Mohapatra MK, Mohanty S, Mohanty BK, Sahu GN (1999) Hypohaptoglobinaemia as a biochemical and epidemiological marker of falciparum malaria. J Assoc Physicians India 47: 874-877.
70. Eisaev BA (1995) [Results of the treatment of patients with recurrence of pulmonary tuberculosis with different types of haptoglobin]. Probl Tuberk: 20-22.
71. Delanghe JR, Langlois MR, Boelaert JR, Van Acker J, Van Wanzeele F, et al. (1998) Haptoglobin polymorphism, iron metabolism and mortality in HIV infection. Aids 12: 1027-1032.
72. Bacq Y, Schillio Y, Brechot JF, De Muret A, Dubois F, et al. (1993) [Decrease of haptoglobin serum level in patients with chronic viral hepatitis C]. Gastroenterol Clin Biol 17: 364-369.
73. Levy AP, Roguin A, Hochberg I, Herer P, Marsh S, et al. (2000) Haptoglobin phenotype and vascular complications in patients with diabetes. N Engl J Med 343: 969-970.
74. Ryndel M, Behre CJ, Brohall G, Prahl U, Schmidt C, et al. (2010) The haptoglobin 2-2 genotype is associated with carotid atherosclerosis in 64-year old women with established diabetes. Clin Chim Acta 411: 500-504.
75. Holme I, Aastveit AH, Hammar N, Jungner I, Walldius G (2009) Haptoglobin and risk of myocardial infarction, stroke, and congestive heart failure in 342,125 men and women in the Apolipoprotein MOrtality RISk study (AMORIS). Ann Med 41: 522-532.
76. Wynn TA (2003) IL-13 effector functions. Annu Rev Immunol 21: 425-456.
77. Collighan N, Giannoudis PV, Kourgeraki O, Perry SL, Guillou PJ, et al. (2004) Interleukin 13 and inflammatory markers in human sepsis. Br J Surg 91: 762-768.

78. Socha LA, Gowardman J, Silva D, Correcha M, Petrosky N (2006) Elevation in interleukin 13 levels in patients diagnosed with systemic inflammatory response syndrome. Intensive Care Med 32: 244-250.
79. Shehata IH, Mostafa MM, Ziada KW, Saeed AM (2007) Role of IL-13 in the pathogenesis of bronchial asthma. Egypt J Immunol 14: 19-27.
80. Kimura M, Hattori T, Matsuda Y, Yoshioka T, Sumi N, et al. (1997) cDNA cloning, characterization, and chromosome mapping of UBE2E2 encoding a human ubiquitin-conjugating E2 enzyme. Cytogenet Cell Genet 78: 107-111.
81. Hara K, Fujita H, Johnson TA, Yamauchi T, Yasuda K, et al. (2014) Genome-wide association study identifies three novel loci for type 2 diabetes. Hum Mol Genet 23: 239-246.
82. Kanazawa T, Ikeda M, Glatt SJ, Tsutsumi A, Kikuyama H, et al. (2013) Genome-wide association study of atypical psychosis. Am J Med Genet B Neuropsychiatr Genet 162b: 679-686.
83. DePaoli A (2014) Leptin in common obesity and associated disorders of metabolism. J Endocrinol.
84. Wang B, Chandrasekera PC, Pippin JJ (2014) Leptin- and leptin receptor-deficient rodent models: relevance for human type 2 diabetes. Curr Diabetes Rev 10: 131-145.
85. Gonzaga NC, Medeiros CC, de Carvalho DF, Alves JG (2014) Leptin and cardiometabolic risk factors in obese children and adolescents. J Paediatr Child Health 50: 707-712.
86. Kerimkulova AS, Lunegova OS, Mirrakhimov AE, Alibaeva NT, Neronova KV, et al. (2014) [Association of leptin with obesity and hypertension in an ethnic Kyrgyz group]. Ter Arkh 86: 49-53.
87. Petrovski S, Fellay J, Shianna KV, Carpenetti N, Kumwenda J, et al. (2011) Common human genetic variants and HIV-1 susceptibility: a genome-wide survey in a homogeneous African population. Aids 25: 513-518.
88. Pryczynicz A, Gryko M, Niewiarowska K, Dymicka-Piekarska V, Ustymowicz M, et al. (2013) Immunohistochemical expression of MMP-7 protein and its serum level in colorectal cancer. Folia Histochem Cytobiol 51: 206-212.
89. Moinzadeh P, Krieg T, Hellmich M, Brinckmann J, Neumann E, et al. (2011) Elevated MMP-7 levels in patients with systemic sclerosis: correlation with pulmonary involvement. Exp Dermatol 20: 770-773.
90. Nastase A, Paslaru L, Niculescu AM, Ionescu M, Dumitrascu T, et al. (2011) Prognostic and predictive potential molecular biomarkers in colon cancer. Chirurgia (Bucur) 106: 177-185.
91. Zhou LF, Jiang L, Li ZH, Kang J (2010) [Change of matrix metalloproteinase-1 and matrix metalloproteinase-7 in serum and bronchoalveolar lavage fluid of patients with idiopathic pulmonary fibrosis and sarcoidosis]. Zhonghua Jie He He Hu Xi Za Zhi 33: 441-444.
92. Leelawat K, Narong S, Wannaprasert J, Ratanashu-ek T (2010) Prospective study of MMP7 serum levels in the diagnosis of cholangiocarcinoma. World J Gastroenterol 16: 4697-4703.
93. Wade TD, Gordon S, Medland S, Bulik CM, Heath AC, et al. (2013) Genetic variants associated with disordered eating. Int J Eat Disord 46: 594-608.

94. Jahanshad N, Rajagopalan P, Hua X, Hibar DP, Nir TM, et al. (2013) Genome-wide scan of healthy human connectome discovers SPON1 gene variant influencing dementia severity. Proc Natl Acad Sci U S A 110: 4768-4773.
95. Kamboh MI, Wang X, Kao AH, Barmada MM, Clarke A, et al. (2013) Genome-wide association study of antiphospholipid antibodies. Autoimmune Dis 2013: 761046.
96. Slavin TP, Feng T, Schnell A, Zhu X, Elston RC (2011) Two-marker association tests yield new disease associations for coronary artery disease and hypertension. Hum Genet 130: 725-733.
97. Chalasani N, Guo X, Loomba R, Goodarzi MO, Haritunians T, et al. (2010) Genome-wide association study identifies variants associated with histologic features of nonalcoholic Fatty liver disease. Gastroenterology 139: 1567-1576, 1576.e1561-1566.
98. Anney R, Klei L, Pinto D, Regan R, Conroy J, et al. (2010) A genome-wide scan for common alleles affecting risk for autism. Hum Mol Genet 19: 4072-4082.
99. Jakubowski A, Rosa MD, Bixler S, Lobb R, Burkly LC (1995) Vascular cell adhesion molecule (VCAM)-Ig fusion protein defines distinct affinity states of the very late antigen-4 (VLA-4) receptor. Cell Adhes Commun 3: 131-142.
100. Ballantyne CM, Entman ML (2002) Soluble adhesion molecules and the search for biomarkers for atherosclerosis. Circulation 106: 766-767.
101. Hwang SJ, Ballantyne CM, Sharrett AR, Smith LC, Davis CE, et al. (1997) Circulating adhesion molecules VCAM-1, ICAM-1, and E-selectin in carotid atherosclerosis and incident coronary heart disease cases: the Atherosclerosis Risk In Communities (ARIC) study. Circulation 96: 4219-4225.
102. Littler AJ, Buckley CD, Wordsworth P, Collins I, Martinson J, et al. (1997) A distinct profile of six soluble adhesion molecules (ICAM-1, ICAM-3, VCAM-1, E-selectin, L-selectin and P-selectin) in rheumatoid arthritis. Br J Rheumatol 36: 164-169.
103. Ohga E, Nagase T, Tomita T, Teramoto S, Matsuse T, et al. (1999) Increased levels of circulating ICAM-1, VCAM-1, and L-selectin in obstructive sleep apnea syndrome. J Appl Physiol (1985) 87: 10-14.
104. Papayianni A, Alexopoulos E, Giamalis P, Gionanlis L, Belechri AM, et al. (2002) Circulating levels of ICAM-1, VCAM-1, and MCP-1 are increased in haemodialysis patients: association with inflammation, dyslipidaemia, and vascular events. Nephrol Dial Transplant 17: 435-441.
105. Schmidt AM, Crandall J, Hori O, Cao R, Lakatta E (1996) Elevated plasma levels of vascular cell adhesion molecule-1 (VCAM-1) in diabetic patients with microalbuminuria: a marker of vascular dysfunction and progressive vascular disease. Br J Haematol 92: 747-750.
106. Spronk PE, Bootsma H, Huitema MG, Limburg PC, Kallenberg CG (1994) Levels of soluble VCAM-1, soluble ICAM-1, and soluble E-selectin during disease exacerbations in patients with systemic lupus erythematosus (SLE); a long term prospective study. Clin Exp Immunol 97: 439-444.
107. Fahey TJ, 3rd, Tracey KJ, Tekamp-Olson P, Cousens LS, Jones WG, et al. (1992) Macrophage inflammatory protein 1 modulates macrophage function. J Immunol 148: 2764-2769.
108. Menten P, Wuyts A, Van Damme J (2002) Macrophage inflammatory protein-1. Cytokine Growth Factor Rev 13: 455-481.

109. Dubois PC, Trynka G, Franke L, Hunt KA, Romanos J, et al. (2010) Multiple common variants for celiac disease influencing immune gene expression. Nat Genet 42: 295-302.
110. Hunt KA, Zhernakova A, Turner G, Heap GA, Franke L, et al. (2008) Newly identified genetic risk variants for celiac disease related to the immune response. Nat Genet 40: 395-402.
111. Njajou OT, Alizadeh BZ, Aulchenko Y, Zillikens MC, Pols HA, et al. (2006) Heritability of serum iron, ferritin and transferrin saturation in a genetically isolated population, the Erasmus Rucphen Family (ERF) Study. Hum Hered 61: 222-228.
112. Yeap BB, Divitini ML, Gunton JE, Olynyk JK, Beilby JP, et al. (2014) Higher ferritin levels, but not serum iron or transferrin saturation, are associated with Type 2 diabetes mellitus in adult men and women free of genetic haemochromatosis. Clin Endocrinol (Oxf).
113. Mainous AG, 3rd, Gill JM, Carek PJ (2004) Elevated serum transferrin saturation and mortality. Ann Fam Med 2: 133-138.
